# Vinyl Azides as Radical Acceptors in the Vitamin B_12_-Catalyzed Synthesis of Unsymmetrical Ketones

**DOI:** 10.1021/acs.orglett.1c03321

**Published:** 2021-11-16

**Authors:** Krzysztof R. Dworakowski, Sabina Pisarek, Sidra Hassan, Dorota Gryko

**Affiliations:** Institute of Organic Chemistry, Polish Academy of Sciences, Kasprzaka 44/52, 01-224 Warsaw, Poland

## Abstract

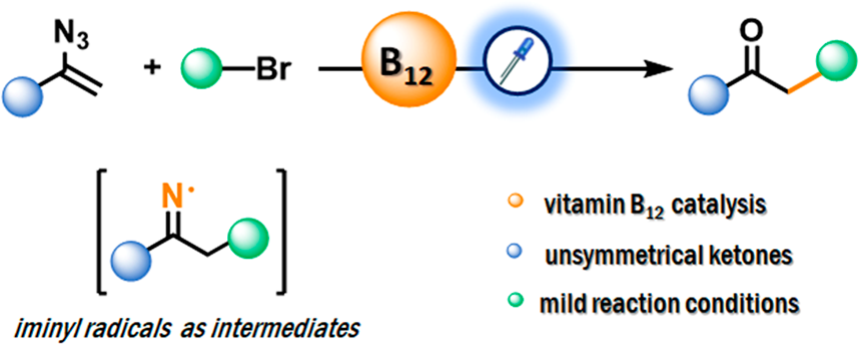

Vinyl azides are very reactive species and as such are useful building blocks, in particular, in the synthesis of N-heterocycles. They can also serve as precursors of ketones. These form in reactions of vinyl azides with nucleophiles or radicals. We have found, however, that under light irradiation vitamin B_12_ catalyzes the reaction of vinyl azides with electrophiles to afford unsymmetrical carbonyl compounds in decent yields. Mechanistic studies revealed that alkyl radicals are key intermediates in this transformation.

Vinyl azides, a conjugated system of alkene and azide moieties, are very reactive species exhibiting multifaceted reactivity.^1^ They react not only as azides but also as nucleophiles, electrophiles, and radical acceptors (Scheme 1). Many of these transformations involve 2*H*-azirines as intermediates that are generated from vinyl azides under thermal, acidic, or photocatalytic conditions.^2^ These reactive species are particularly important in the synthesis of nitrogen-containing heterocycles.^3^

**Scheme 1:**
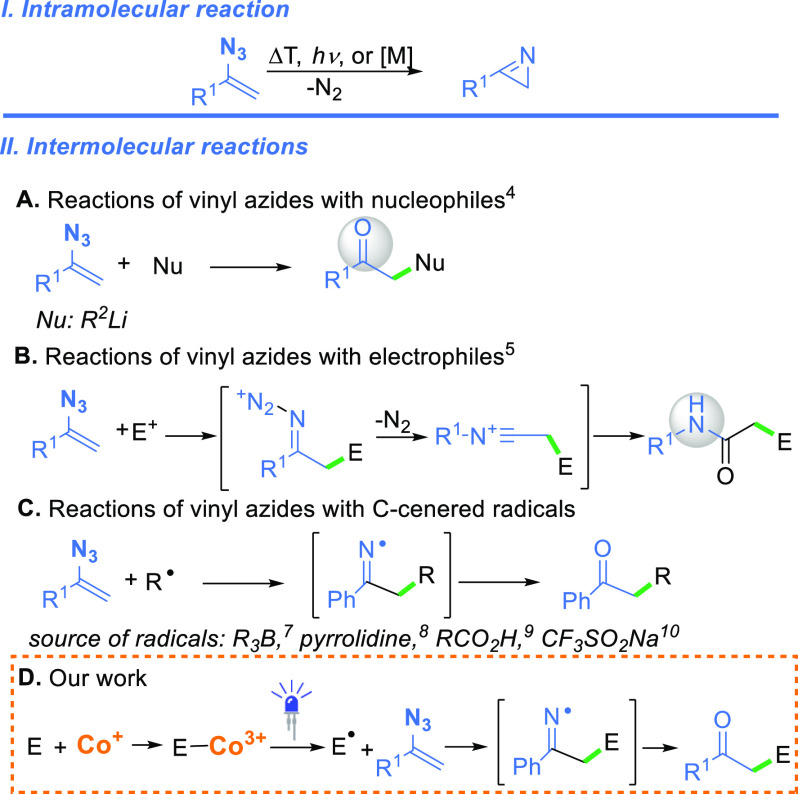
Reactivity of Vinyl Azides

Vinyl azides react with nucleophiles affording iminyl species, which upon hydrolysis generate the corresponding ketone (Scheme 1A).^4^ Iminyl radicals are also intermediates in the synthesis of β-substituted enamines from azides.^5^ On the contrary, their most common reaction with electrophiles leads to amides in which the nitrogen atom originates from the azide moiety (Scheme 1B).^6^ A key intermediate in this transformation, the iminodiazonium ion, forms when an electrophile attacks the β-carbon of vinyl azide. The subsequent Schmidt-type rearrangement furnishes the desired amide. In 1975, Suzuki reported the reaction of vinyl azides with trialkyl boranes affording alkyl ketones (Scheme 1C).^7^ This process is radical in nature and proceeds via a chain mechanism involving iminyl radicals as intermediates. Other radical sources have been shown to be suitable for reaction with vinyl azides; these include pyrrolidines,^8^ carboxylic acids,^9^ trifluoromethanesulfonates,^10^ and thiols.^11^

To expand the synthetic toolbox of chemical transformations of vinyl azides, we wondered whether vitamin B_12_-catalysis would enable their reaction with electrophiles (Scheme 1D). Vitamin B_12_ [**1**, cobalamin (Figure 1)] has been recognized as an efficient Co catalyst that not only mimics natural processes but also promotes chemical reactions unprecedented in living systems.^12^ Its catalytic activity originates from the redox properties of the central Co ion. After reduction, the Co(I) complex, as a supernucleophile, easily reacts with electrophiles giving alkyl-cobalamins (Scheme 2). These, at higher temperatures or higher levels of light irradiation, are prone to homolytic cleavage generating radicals. Thus, vitamin B_12_ catalysis enables formation of radicals from various electrophilic precursors; these include organic halides,^13,14^ epoxides,^15^ diazo compounds,^16^ and strained molecules.^17^

**Figure 1:**
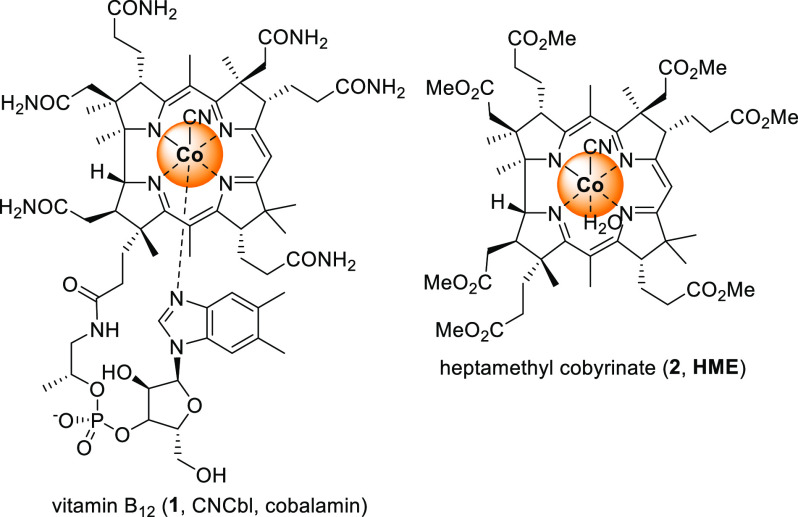
Structures of vitamin B_12_ and heptamethyl cobyrinate.

**Scheme 2:**
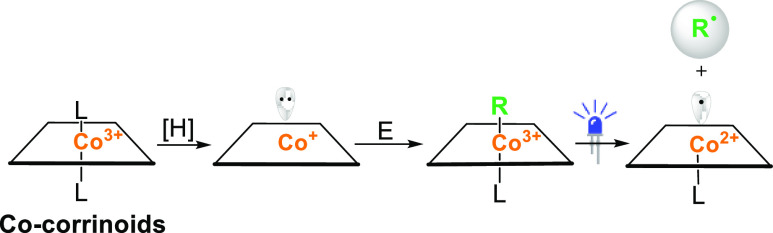
Catalytic Mode for Vitamin B_12_-Catalyzed Generation of Radicals

Vinyl azides have recently emerged as effective radical acceptors. Xu and co-workers prepared β-amino-ketones from *N*-Ph-pyrrolidines and vinyl azides.^8^ The Nevado group reported a Ag(I)-promoted synthesis of cyclic ketones involving alkyl radicals generated from carboxylic acids.^18^ While investigating the mechanism of azidoalkylation of alkenes with diazoacetate, the Doyle group proposed that the addition of α-ester radicals to vinyl azides, followed by denitrogenative fragmentation and hydrolysis, afforded ketones in 47% yield.^19^ The synthesis of unsymmetrical, linear ketones, however, remained elusive. On the basis of this reactivity mode and the fact that vitamin B_12_ generates radicals from electrophiles, we envisaged that they should react with vinyl azides to give iminyl species that in turn will transform into ketones.

In our initial experiment, α-phenyl vinyl azide (**3a**) was reacted with (3-bromopropyl)benzene (**4a**) in the presence of aqua(cyano)heptamethyl cobyrinate (**2**, HME) as the Co catalyst and the Zn/NH_4_Cl reducing system under blue light irradiation (Scheme 3). To ensure subsequent hydrolysis of an imine intermediate, water was added to the reaction mixture. Indeed, the desired product **5aa** was isolated in 16% yield. In contrast to our results, a similar reaction of vinyl azides with methyl 2-bromo-2-arylethanoate under visible light photoredox catalysis was shown to generate iminyl radical but following subsequent C–N bond-forming cyclization and aromatization yielded quinoline derivatives instead.^20^

**Scheme 3:**
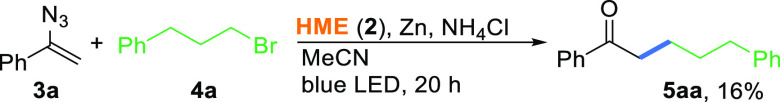
Model Reaction of α-Phenyl, Vinyl Azide (**3a**) with 3-Phenylpropyl Bromide (**4a**) Reaction conditions: vinyl azide **3a** (2.5 equiv), alkyl bromide **4a** (1 equiv), Zn (6 equiv), NH_4_Cl (3 equiv), MeCN (0.1 M), blue light (446 nm, 7 W), 20 h.

The reaction conditions were optimized with regard to solvent, additives, amount of reagents, time, and a source of light (for details, see the Supporting Information). Zhou and co-workers reported that the yield of the photocatalyzed reaction of vinyl azides with methyl 2-bromo-2-phenylethanoate, leading to quinolines, increased upon the addition of 18-crown-6 ether.^20^ The exact role of this reagent, however, was not explained. When we added 18-crown-6 ether to the model reaction mixture, an appreciable increase in the yield, to 52%, was also observed (Table 1, entry 1). For reactions performed in the dark at 60 °C, in an oil bath or under microwave irradiation, the yield decreased to 31% or 39%, respectively (entry 2 or 3, respectively). Consequently, the photocatalytic approach was further developed.

**Table 1:**
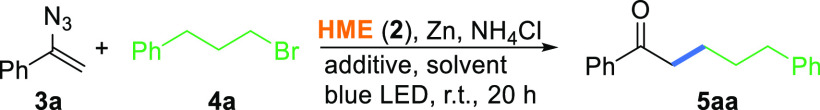
Optimization of the Reaction Conditions for the Alkylation of Vinyl Azide **3a** with Alkyl Bromide **4a**^a^

entry	solvent	changes	light (W)	yield (%)^c^
1	MeCN	–	7	52
2	MeCN	60 °C, oil bath	–	31
3	MeCN	60 °C, microwave	–	32
4	DMA	–	7	27
5	DMF	–	7	64
6	DMF	HME instead B_12_	7	26
7	DMF	–	3	28
8	DMF	30 min instead of 20 h	10	37
9^b^	DMF	H_2_O as an additive	7	82

a General conditions: vinyl azide **3a** (2.5 equiv), alkyl bromide **4a** (0.25 mmol, 1.0 equiv), HME (**2**, 5 mol %), NH_4_Cl (1.5 equiv), Zn (3.0 equiv), 18-crown-6 (1.5 equiv), H_2_O (3 equiv), and solvent (0.1 M), 20 h, blue light (450 nm).

b H_2_O (1.5 equiv).

c Yields based on HPLC measurements.

In the next step, other solvents were tested, and to control the amount of water added, anhydrous solvents were used (see the Supporting Information). Notably, the reaction efficacy increased in DMF (entry 5). Ketone **5aa** was obtained in 64% yield, and the results were highly reproducible. The replacement of cobyrinate **2** with parent vitamin B_12_ (**1**) resulted in a significant decrease in the yield to 26% (entry 6).

The optimum reaction yield was achieved after 20 h when the conversion of both vinyl azide **3a** and alkyl bromide **4a** was complete. Altering the amount of vinyl azide, alkyl bromide, zinc, or ammonium chloride did not improve the yield of ketone **5aa**. A very important factor was, however, the selection of the light power. Under irradiation with a single 3 W LED, the yield significantly decreased while with a 10 W LED full conversion was observed after only 30 min, but product **5aa** formed in only 37% yield (entries 7 and 8). Optimizing the amount of water in the reaction mixture facilitated a notable increase in the yield (for details, see the Supporting Information). Decreasing it to 1.5 equiv proved to be sufficient for the *in situ* hydrolysis of the imine intermediate and at the same time did not accelerate the decomposition of vinyl azide **3a** as the yield reached 82% (entry 9).

After optimization studies, the scope of the developed method was explored utilizing a broad spectrum of alkyl halides **4**, **6–8**, and vinyl azides **3** (Scheme 4; see pages S4–S24 of the Supporting Information). Following the general trend for vitamin B_12_-catalyzed reactions, alkyl chloride **6** and tosylate **7** remained unreactive while iodide **8** was less reactive than respective bromide **4a**. Consequently, as shown in Scheme 4, a broad range of alkyl bromides **4** reacted with vinyl azide **3** leading to unsymmetrical ketones **5** in decent yields (30–85%).

**Scheme 4:**
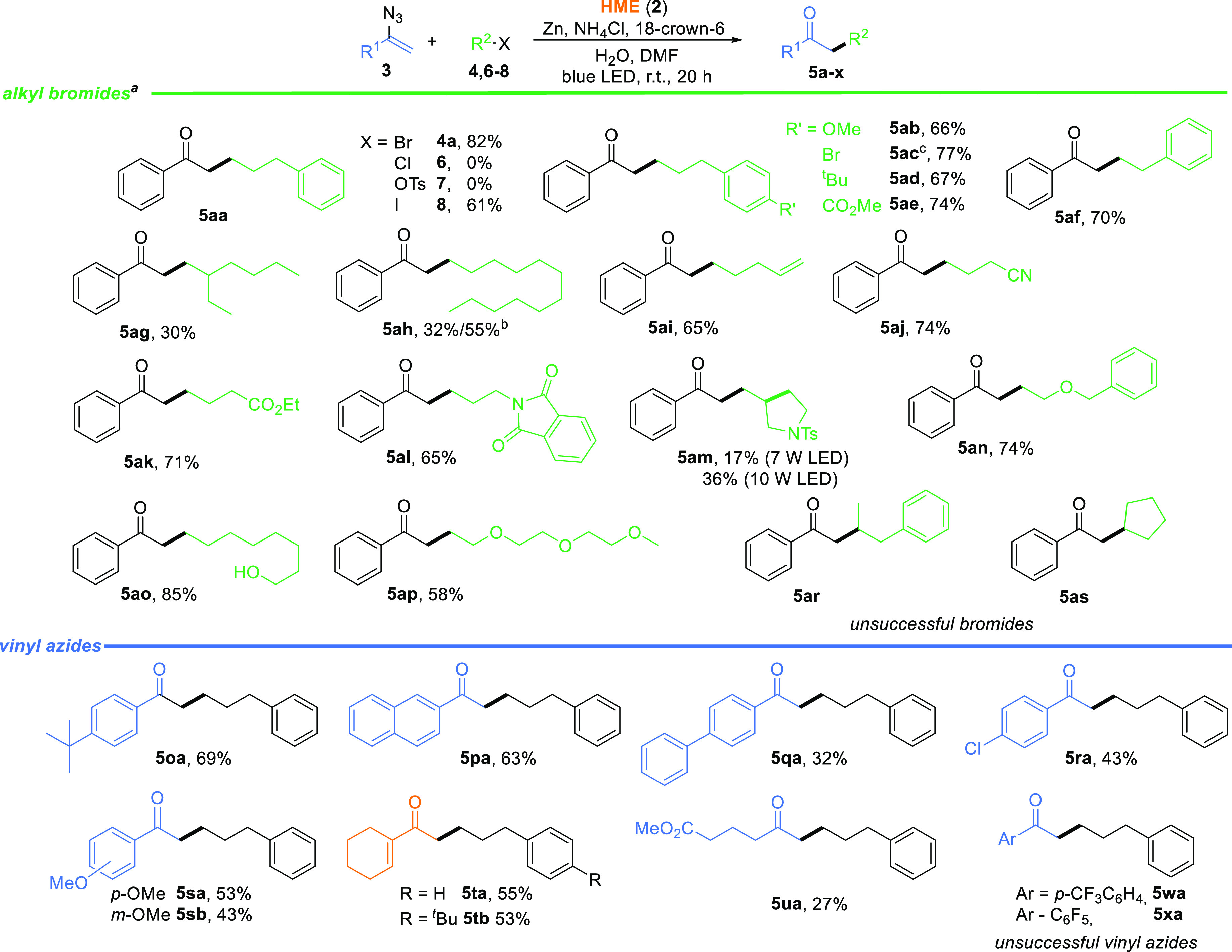
Scope and Limitations Reaction conditions: alkyl bromide **4** (0.25 mmol, 1.0 equiv), vinyl azide **3** (2.5 equiv), HME (**2**, 7.5 mol %), NH_4_Cl (1.5 equiv), Zn (3.0 equiv), 18-crown-6 (1.5 equiv), H_2_O (1.5 equiv), dry DMF (*c* = 0.1 M), blue LED (7 W), 20 h. Reaction in dry toluene (*c* = 0.1 M), 20 h. Contains 5% of **5aa**.

As expected, functional groups on the phenyl ring of the alkyl bromides, regardless if they were electron-donating or -withdrawing groups, did not affect the yield. These examples (**5ab–ae**) emphasize the compatibility of ester and alkoxy moieties with the developed conditions. Other functional groups, including alkene (**5ai**), cyano (**5aj**), carboxyl (**5ak**), protected amino (**5al** and **5am**), and hydroxyl (**5ao**) groups, are also well tolerated. Noticeably, a key factor influencing the yield of the developed transformation is the solubility of an alkyl bromide in DMF. Ketones with lipophilic alkyl chains (**5ag** and **5ah**) were obtained in low yields that significantly improved when the reaction was performed in toluene. Under the developed conditions, secondary bromides remained unreactive, most likely due to steric constraints.

In general, the reactivity of vinyl azides strongly depends on the α-substituent, typically aryl, alkyl, heteroatom, ester, or carbonyl groups.^1b,1c^ To gain insight into the effect on their alkylation with alkyl bromides, various vinyl azides **3** were screened. The developed conditions enabled the synthesis of aryl and alkyl ketones (**5oa–sa**), though phenyl vinyl azides **3wa** and **3xa** bearing electron-withdrawing groups at the aryl moiety, with diminished nucleophilic character, remained unreacted and were recovered from the reaction mixture. Furthermore, α,β-unsaturated vinyl azide **3t** was synthesized and exposed to the standard conditions. Desired products **5ta** and **5tb** formed in reasonable yields, and such behavior is quite rare for only α,β-unsaturated azides.^8,21^ Even alkyl vinyl azide **3u** exhibited reactivity under the developed conditions.

To gain some insight into the mechanism of the developed reaction, a series of control experiments were conducted (Scheme 5). In the first instance, background experiments revealed that all reagents, a catalyst, a reductant, and light are required for the efficient reaction; otherwise, the desired product was not formed

**Scheme 5:**
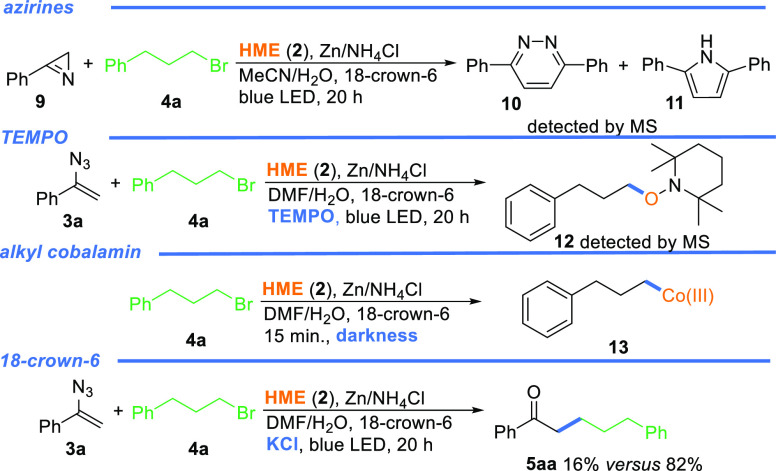
Mechanistic Studies

Under light irradiation or thermal conditions, the common feature of vinyl azides is their transformation into azirines. To verify their involvement in the catalytic cycle, we had prepared azirine **9** and subjected it to our standard conditions. The reaction did not lead to the desired product; instead, pyridazine **10** and pyrrole **11** were detected by GCMS (*m*/*z* 232.2 and 219.2, respectively). Therefore, azirines were excluded as intermediates. The addition of TEMPO diminished the reaction yield significantly, suggesting a radical mechanism. On the basis of our previous work, we assumed that alkyl-cobalamin **13** is generated and the homolytic cleavage of the Co–C bond generates alkyl radicals. Thus, alkyl bromide was reacted with HME (**2**) under the developed conditions in the dark. MS analysis showed a peak at 1155.7 Da, corroborating the formation of alkyl-cobalt(III) complex **13**.

The strong influence of 18-crown-6 as an additive on the reaction outcome was puzzling. Its role in the synthesis of quinolines from vinyl azides was also not explained by Zhou.^17^ We assumed that the complexation of reaction components, presumably an ammonium ion, could be involved. To disturb this process, we performed the model reaction with the addition of KCl as 18-crown-6 exhibits a particularly strong affinity for K^+^ (10^6^ M^–1^ MeOH). The diminished reaction yield corroborates our assumption, but the question of why remains open.

On the basis of the experiments described above, we propose a mechanism for the developed reaction, depicted in Scheme 6. The key steps involve the Co-catalyzed generation of alkyl radicals **III** and their reaction with vinyl azide **IV** yielding α-azido radical **V**. Denitrogenative fragmentation leads to iminyl radical **VI**, a reactive species proposed in reported radical reactions that after reduction to an anion^10^ presumably by zinc and subsequent protonation gives imine **VII**. Its hydrolysis affords the desired ketone **VIII**.

**Scheme 6:**
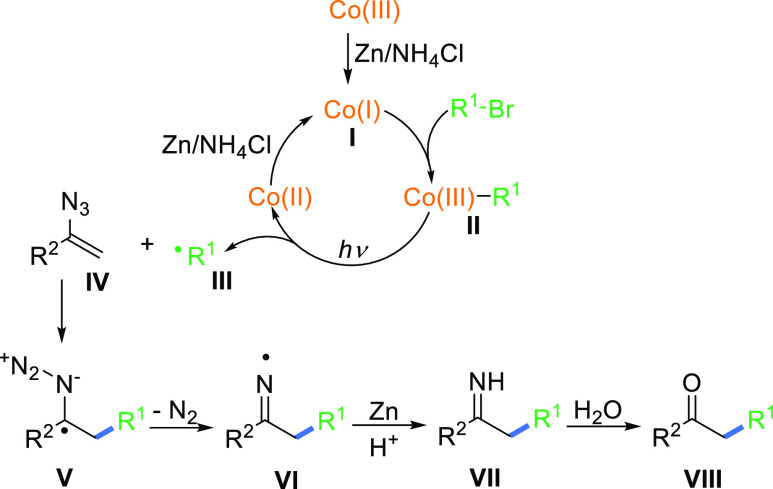
Plausibe Mechanism for the Reaction of Vinyl Azides with Alkyl Bromides

In conclusion, we have shown that vitamin B_12_ catalysis facilitates the reaction of vinyl azides with electrophiles leading to unsymmetrical ketones. Under the developed conditions, electrophilic alkyl bromides form C-centered nucleophilic radicals that react with electron rich alkenes exhibiting enamine-like nucleophilicity. This methodology expands the chemical toolbox of transformations for vinyl azides; now their reactions with both nucleophiles and electrophiles give access to ketones.

## Supporting Information Available

Experimental details and procedures, optimization studies, mechanistic experiments, and spectral data for all new compounds (PDF)
