# Supplementary material for: Vinyl Azides as Radical Acceptors in the Vitamin B12-Catalyzed Synthesis of Unsymmetrical Ketones
Source: Org Lett. 2021 Nov 16;23(23):9068–72. doi: 10.1021/acs.orglett.1c03321 (PMC8650103; doi:10.1021/acs.orglett.1c03321)

Supporting Information

Vinyl Azides as Radical Acceptors in the Vitamin B_{12}-Catalyzed Synthesis of Unsymmetrical Ketones

Krzysztof R. Dworakowski‡, Sabina Pisarek‡, Sidra Hassan, Dorota Gryko*

Institute of Organic Chemistry Polish Academy of Science

Kasprzaka 44/52, 01-224 Warsaw, Poland

e-mail: dorota.gryko@icho.edu.pl

Table of Contents

1. General information S3
2. Optimization studies S4
2.1. Background reactions S4
2.2. Optimization of the reaction time S5
2.3. Optimization of the light source S5
2.4. Optimization of the vinyl azide to alkyl bromide ratio S5
2.5. Optimization of the amount of the catalyst S6
2.6. Optimization of the amount of zinc and ammonium chloride S6
2.7. Optimization of the amount of water S6
3. General procedures S7
3.1. General procedures for ketone synthesis S7
3.2. Procedure for 1 mmol scale S7
3.3. Vinyl azide synthesis S8
4. Reaction setup S9
5. Mechanistic studies S10
5.1. Reaction with no light activation – mass spectrometry studies S10
5.2. Reaction with radical trap S11
5.3. Reaction of 3-phenyl-2H-azirine (**S6**) with organic bromide (**S2**) S11
6. Scope and characterization of new compounds S13
7. Literature S22
8. ^{1}H NMR and ^{13}C NMR spectra S23

1. General information

General Procedures. Unless otherwise noted, reactions were performed with the exclusion of air. All photochemical reactions were performed in 10 mL glass vials sealed with rubber septa. Reactions were monitored by gas chromatography (GC, specification below) or thin-layer chromatography (TLC) on Merck silica gel (GF254, 0.20 mm thickness), visualizing with UV-light or the dinitrophenylhydrazine (DNP) stain. Colum chromatography was performed using Merck silica gel 60 (230-400 mesh). GC yields were calculated based on using dodecane as an internal standard.

Materials. Commercial reagents and solvents were purchased from Sigma-Aldrich, Fluorochem, or TCI, and used as received unless otherwise noted. Deuterated chloroform ($CDCl_3$) was purchased from Eurisotop. All vinyl azides **3** were synthesized according to literature procedures.[1],[2] Chloride **6** and iodide **8** were purchased from Sigma-Aldrich. Bromides **4ab,**[3] **4ac,**[4] **4ad,**[5] **4ae,**[6] **4am**[7] and tosylate **7**[8] were synthesized according to literature procedures. Before the reaction, zinc was activated by the following method: a) washing with 10% HCl, b) grinding, c) washing with H_2O, acetone, MeOH, and Et_2O, d) drying in a vacuum.

Instrumentation.

• <u>NMR Spectroscopy:</u> ^{1}H and ^{13}C NMR spectra were recorded at 25 °C on a Bruker 400 MHz or 500 MHz instrument with TMS as an internal standard. NMR chemical shifts are reported in ppm and referenced to the residual solvent peak of $CDCl_3$ (7.26 ppm - ^{1}H NMR and 77.16 ppm - ^{13}C NMR). Multiplicities are indicated by singlet (s), doublet (d), triplet (t), quartet (q), multiplet (m) and broad (br). Coupling constants (*J*) are reported in Herz. All data analysis was performed using MestReNova software package.

• <u>GC/MS Chromatography:</u> GC/MS analyses were performed using Shimadzu GCMS-QP2010 SE gas chromatograph with FID detector and Zebron ZB 5Msi column.

• <u>Elemental Analysis:</u> Elemental analysis (N, H, C, S) were performed on PERKIN-ELMER 240 Elemental Analyzer.

• <u>High Resolution Mass Spectrometry:</u> High-resolution mass spectra (HRMS) were recorded on a Waters AutoSpec Premier instrument using electron ionization (EI) or a Waters SYNAPT G2-S HDMS instrument using electrospray ionization (ESI) with time of flight detector (TOF).

2. Optimization details

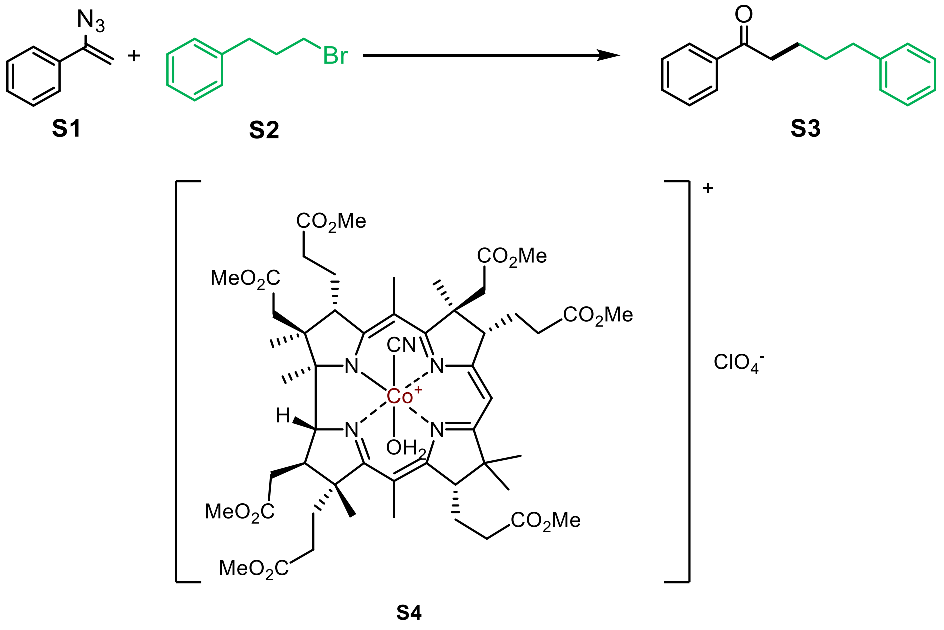

Scheme S1. Model substrates and the catalyst structure

2.1. Table S1. Background reactions[a]

No	*Deviation from reaction conditions*	*Yield of S3 [%]*
1	no HME	0
2	no NH_4Cl	28
3	no H_2O	26
4	no 18-crown-6	14
5	no Zn	0
6	no light	11
7	Mn as reductant	13
8	no degassing	25
9	microwave irradiation 60 °C, 1 h	39
10	60 °C	31

[a]Reaction conditions: 1-bromo-3-phenylpropane (**S2**, 0.25 mmol, 1 equiv), phenyl vinyl azide (**S1**, 2.5 equiv), HME (**S4,** 5 mol%), NH_4Cl (3 equiv), Zn (6 equiv), 18-crown-6 (1.5 equiv), H_2O (3 equiv) and dry DMF(c = 0.1 M), 16 h, 7 W single blue LED.

2.2. Table S2. Optimization of the reaction time[a]

No	*Reaction time [h]*	*Yield of S3 [%]*
1	0	1
2	0.5	5
3	1	8
4	2	17
5	4	35
6	8	51
**7**	**20**	**64**
8	48	67

[a]Reaction conditions: 1-bromo-3-phenylpropane (**S2,** 0.25 mmol, 1 equiv), phenyl vinyl azide (**S1,** 2.5 equiv), HME (**S4,** 5 mol%), NH_4Cl (3 equiv), Zn (6 equiv), 18-crown-6 (1.5 equiv), H_2O (3 equiv) and dry DMF(c = 0.1 M), 7 W single blue LED.

2.3. Table S3. Optimization of the light source[a]

No	*Light source*	*Yield of S3 [%]*
1[b]	10 W single blue LED	37
2	3 W single blue LED	28
3	white LED stripe	14
4	purple LED stripe	23
5	blue LED stripe	15
6	green LED stripe	16

[a]Reaction conditions: 1-bromo-3-phenylpropane (**S1,** 0.25 mmol, 1 equiv), phenyl vinyl azide (**S2,** 2.5 equiv), HME (**S4,** 5 mol%), NH_4Cl (3 equiv), Zn (6 equiv), 18-crown-6 (1.5 equiv), H_2O (3 equiv) and dry DMF(c = 0.1 M), 20 h. [b]Reaction conducted for 30 min.

2.4. Table S4. Optimization of the vinyl azide S1 to alkyl bromide S2 ratio[a]

No	*S1 [eqiuv.]*	*S2 [eqiuv.]*	*Yield of S3 [%]*
1	1	1	13
2	1.5	1	42
3	2	1	58
**4**	**2.5**	**1**	**64**
5	3.5	1	60
6	1	2	26

[a]Reaction conditions: HME (**S4,** 5 mol%), NH_4Cl (3 equiv), Zn (6 equiv), 18-crown-6 (1.5 equiv), H_2O (3 equiv) and dry DMF (c = 0.1 M), 20 h, 7 W single blue LED.

2.5. Table S5. Optimization of the catalyst (HME, S4) amount[a]

No	*Amount of HME [mol%]*	*Yield of S3 [%]*
1	2.5	47
2	5	64
**3**	**7.5**	**65**
4	10	69
5	12.5	53
6	15	48

[a]Reaction conditions: 1-bromo-3-phenylpropane (**S2,** 0.25 mmol, 1 equiv), phenyl vinyl azide (**S1,** 2.5 equiv), NH_4Cl (3 equiv), Zn (6 equiv), 18-crown-6 (1.5 equiv), H_2O (3 equiv) and dry DMF (c = 0.1 M), 20 h, 7 W single blue LED.

2.6. Table S6. Optimization of the amount of zinc and ammonium chloride[a]

No	*Zn [eqiuv.]*	*NH_4Cl [eqiuv.]*	*Yield of S3 [%]*
1	1.5	3	9
**2**	**3**	**1.5**	**71**
3	3	3	65
4	3	4.5	64
5	4.5	3	66
6	6	3	69
7	9	3	64

[a]Reaction conditions: 1-bromo-3-phenylpropane (**S2,** 0.25 mmol, 1.0 equiv), phenyl vinyl azide (**S1,** 2.5 equiv), HME (**S4,** 7.5 mol%), 18-crown-6 (1.5 equiv), H_2O (3 equiv) and dry DMF (c = 0.1 M), 20 h, 7 W single blue LED.

2.7. Table S7. Optimization of the amount of water[a]

No	*H_2O [eqiuv.]*	*Yield of S3 [%]*
1	1	55
**2**	**1.5**	**80**
3	3	69
4	4.5	69
5	6.75	68
6	3[b]	26

[a]Reaction conditions: 1-bromo-3-phenylpropane (**S2,** 0.25 mmol, 1 equiv), phenyl vinyl azide (**S1,** 2.5 equiv), HME (**S4,** 7.5 mol%), NH_4Cl (1.5 equiv), Zn (3 equiv), 18-crown-6 (1.5 equiv), and dry DMF (c = 0.1 M), 20 h, 7 W single blue LED. [b]Addition of H_2O (3 equiv) after the reaction was compleated.

3. General procedures

3.1. *General procedure for ketone synthesis*

A glass vial (10 mL) equipped with a cross-shaped stirring bar and sealed with a septum was charged with catalyst **S4** (23 mg, 19 µmol, 7.5 mol%), vacuum dried ammonium chloride (20 mg, 0.37 mmol, 1.5 equiv), freshly melted and vacuum dried 18-crown-6 (90 mg, 0.34 mmol, 1.4 equiv), next dry DMF (2.5 ml, 0.1 mol/dm^3) and water (7 µl, 0.4 mmol, 1.4 equiv) were added. The reaction mixture was stirred for one minute to dissolve all reagents, then activated zinc (50 mg, 0.76 mmol, 3 equiv) was added, and resulting mixture was degassed by purging with argon and sonication for 20 min. The color of the solution should change, from red to dark green, if not, after degassing the reaction mixture must be stirred on magnetic stirrer until the color change to dark green (around 5 min.). Subsequently alkyl bromide **S2** (50 mg, 0.25 mmol, 1.0 equiv) and vinyl azide **S1** (90 mg, 0.63 mmol, 2.5 equiv) were added. Then the reaction mixture was irradiated with 7 W single LED light for 20 h. Products were purified by column chromatography (SiO_2, hexane:diethyl ether, 0-20%).

3.2. *Procedure for 1 mmol scale*

A glass vial (10 mL) equipped with a cross-shaped stirring bar and sealed with a septum was charged with catalyst **S4** (92 mg, 76 µmol, 7.5 mol%), vacuum dried ammonium chloride (80 mg, 1.5 mmol, 1.5 equiv), freshly melted and vacuum dried 18-crown-6 (360 mg, 1.4 mmol, 1.4 equiv), next dry DMF (7.5 ml, 0.133 mol/dm^3) and water (28 µl, 1.5 equiv) were added. The reaction mixture was stirred for one minute to dissolve all reagents, then activated zinc (200 mg, 3.0 mmol, 3.0 equiv) was added, and resulting mixture was degassed by purging with argon and sonication for 20 min. The color of the solution changed from red to dark green. Subsequently alkyl bromide **S2** (200 mg, 1.0 mmol, 1.0 equiv) and vinyl azide **S1** (360 mg, 2.5 mmol, 2.5 equiv) were added. Then the reaction mixture was irradiated with 40 W single LED light for 3.5 h and after work-up and purification yielded the desired product in 104 mg, 44% yield.

3.3. *Vinyl azide synthesis*

Method A:

Synthesis of styrene dibromides precursors: To a stirred mixture of olefin (10 mmol), and, LiBr (12 mmol, 1.2 equiv.), in acetic acid (15 ml) and $NaIO_4$ (25 mol%) was added portion wise. The reaction was monitored by TLC. After completion of the reaction, it was diluted with water and extracted with CH_2Cl_2 (25 ml x 3). The organic layers were washed with dilute solution of $NaHCO_3$, Na_2SO_3 and brine and dried over anhydrous Na_2SO_4. After removal of the solvent, the crude residue was purified with column chromatography using (silica gel; hexane, ethyl acetate) and concentrated in vacuo to afford styrene dibromide products.

To a solution of styrene dibromide (6.5 mmol) in dry DMF (25 mL) was added NaN_3 (19.5 mmol). After the reaction mixture was stirred for 24 h at room temperature and then diluted with water, the product was extracted with diethyl ether. The combined organic layers were washed with water (3 × 10 mL) and dried with anhydrous Na_2SO_4. After removal of the solvent, the crude residue was purified with column chromatography using (silica gel; hexane) and concentrated in vacuo to afford vinyl azide products.

Method B (for azides 3sa, 3t, 3ua):

To a solution of alkyne substrate (0.5 mmol), $TMSN_3$ (1.0 mmol) and H_2O (1.0 mmol) in DMSO (2 mL) at 80°C, Ag_2CO_3 (0.05 mmol) was added. The mixture was then stirred for 1.5-2.0 h until substrate was consumed as indicated by TLC. The resulting mixture was concentrated and taken up by dichloromethane (3 × 15 mL). The organic layer was washed with brine (3 × 40 mL), dried over $MgSO_4$ and concentrated under reduced pressure. Crude product was purified with column chromatography (silica gel; hexane) and concentrated in vacuo to afford vinyl azide products.

4. Reaction setup

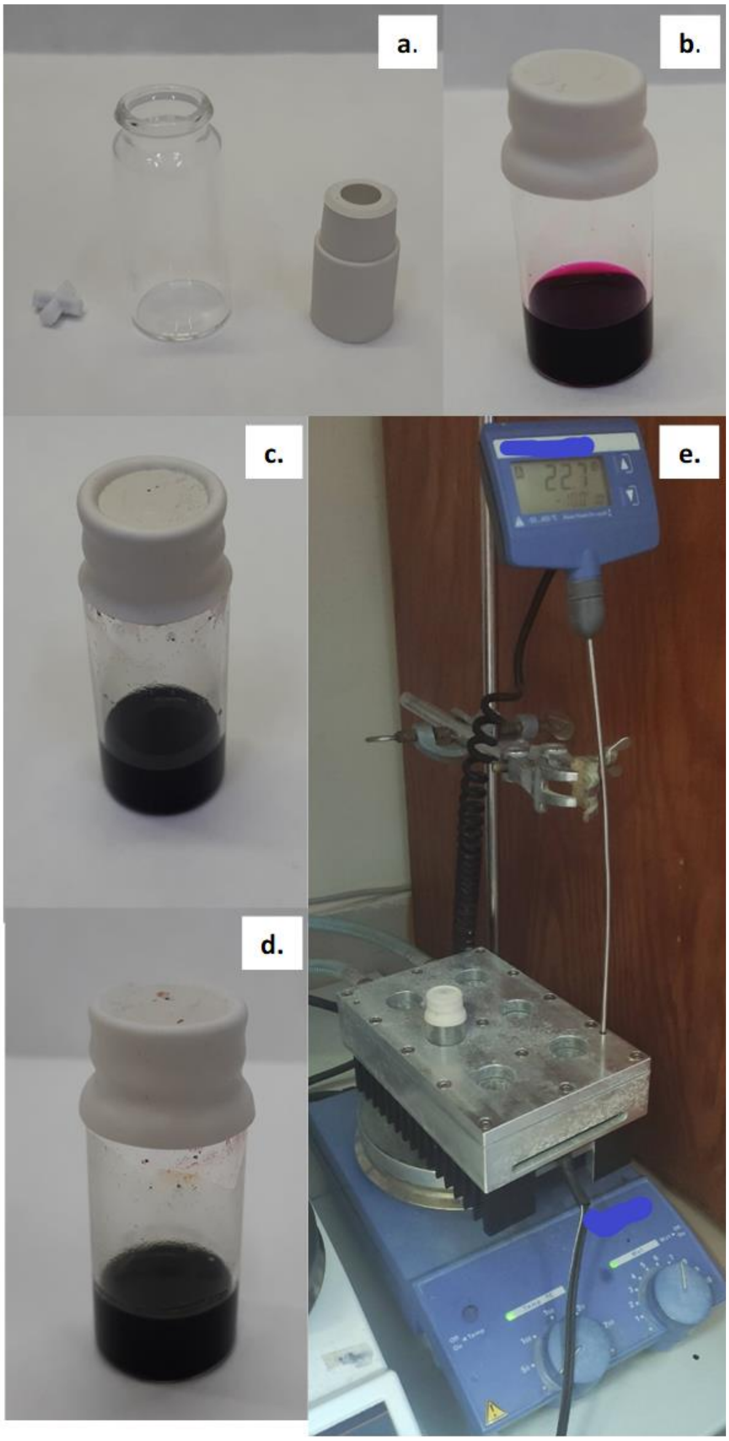

Fig. S1 a) glass vial, rubber septum and cross-shaped stirring bar, **b)** the reaction mixture before degassing, **c)** the reaction mixture after degassing, **d)** the reaction mixture at the end, **e)** photoreactor setup, from top to bottom: thermocouple, cooling block, radiator with LEDs and magnetic stirrer.

Reactions were carried out under blue light irradiation on a single diode (LT-2855 royal blue, λ_{max}: 446 nm, 7W), distance from the reaction vessel: 6 mm.

5. Mechanistic studies

5.1. Reaction with no light activation – mass spectrometry studies

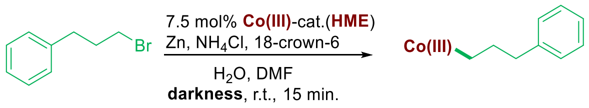

Scheme S2. Formation of cobalt-alkyl complex

The reaction was performed in the dark (vial was covered with alumina foil) according to the general procedure. After 15 min. of stirring in darkness a sample of crude reaction mixture was taken, and its composition was studied by LRMS ESI(+). Signal at 1155,7 corresponds to the alkyl complex.

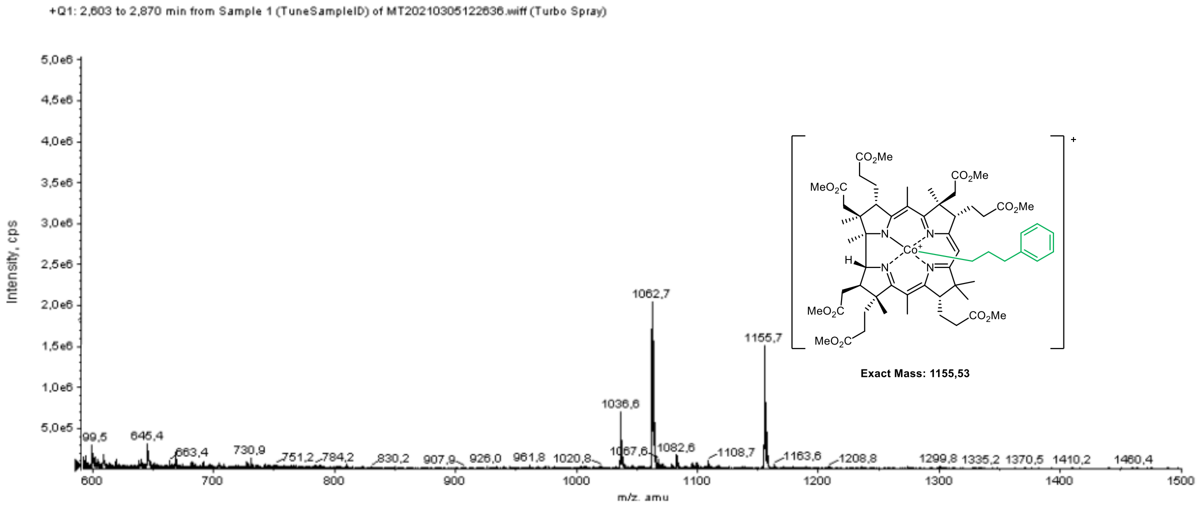

Fig. S2 LRMS ESI(+) spectrum of crude reaction mixture in darkness.

5.2. Reaction with radical trap

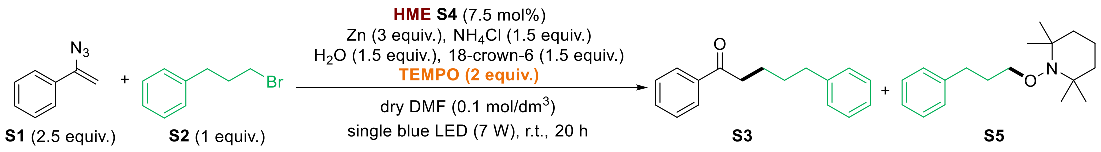

Scheme S3. Reaction in the presence of TEMPO

The reaction was performed according to the general procedure with addition of 2 equiv of TEMPO. Reaction was monitored by GC/MS. After 20 hours product **S3** was obtained in 16% yield. The peak at 7.24 min. corresponds to adduct **S5**.

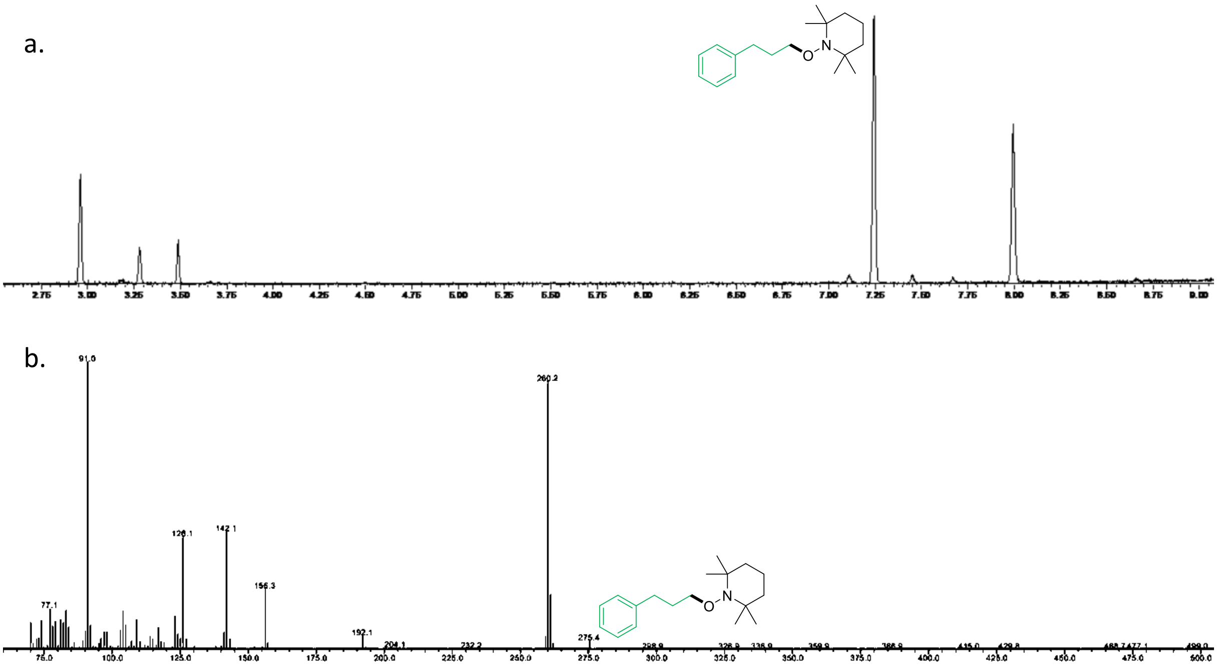

Fig. S3 Analysis of reaction with addition of TEMPO: **a)** GC chromatogram of the crude reaction mixture, **b)** LRMS (EI) spectrum of peak at 7.24 min.

5.3. Reaction of 3-phenyl-2H-azirine (S6) with organic bromide (S2)

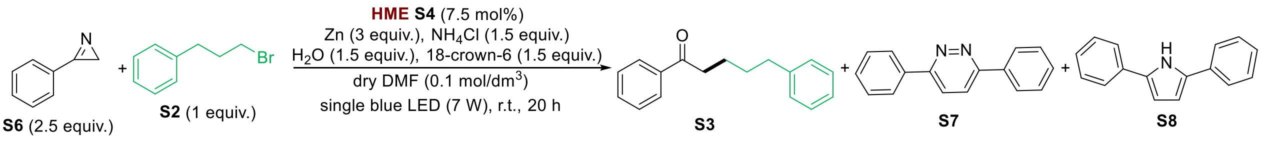

Scheme S4. Reaction with azirine instead of vinyl azide

The reaction was performed according to the general procedure with 3-phenyl-*2H*-azirine (**S6**) instead of 1-azidostyrene (**S1**). The reaction was monitored by GC/MS. After 20 hours product **S3** was not detected. Only products resulting from 3-phenyl-*2H*-azirine decomposition were observed, at 8.92 min. 3,6-diphenylpyridazine, and at 9.13 min. 2,5-diphenylpyrrole.

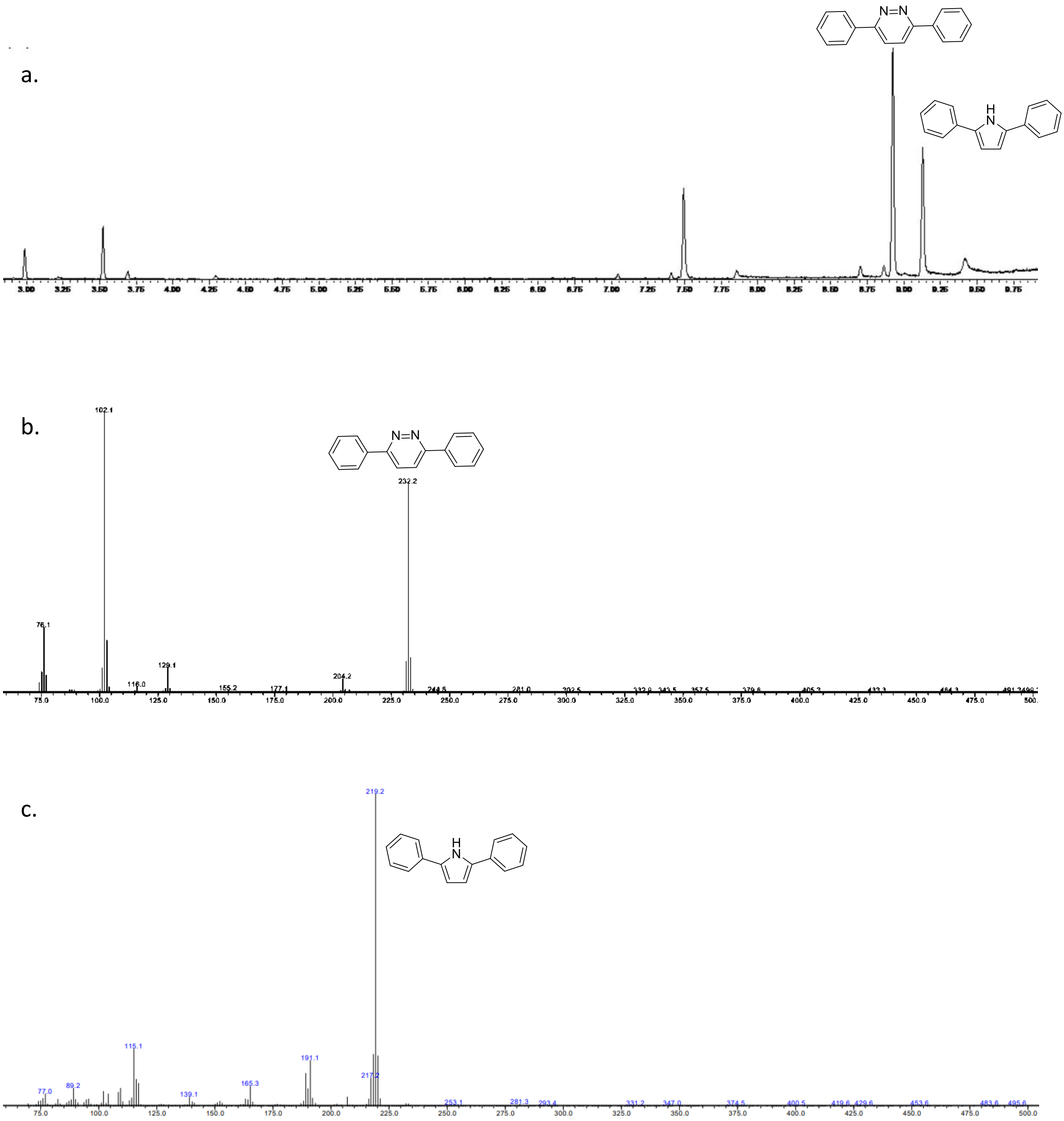

Fig. S4 Analysis of reaction with 3-phenyl-*2H*-azirine (**S5**): **a)** GC chromatogram of crude reaction mixture, **b)** LRMS (EI) spectrum of peak at 8.92 min., **c)** LRMS (EI) spectrum of peak at 9.13 min.

6. Scope and characterization of new products

1,5-Diphenylpenan-1-one (5aa)[9]

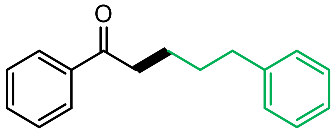

Following the general procedure compound **5aa** was obtained from 1-bromo-3-phenylopropane (**S2,** 50 mg, 0.25 mmol) and 1-azidostyrene (**S1,** 91 mg, 0.625 mmol). The crude product was purified by column chromatography (SiO_2, hexane:diethyl ether, 0-20%) to afford 48 mg, yield = 81%, as colorless oil.

^{1}H NMR (400 MHz, $CDCl_3$): δ 8.0-7.9 (m, 2H), 7.6-7.5 (m, 1H), 7.5-7.4 (m, 2H), 7.3-7.2 (m, 2H), 7.3-7.1 (m, 3H), 2.99 (t, *J* = 7.1 Hz, 2H), 2.68 (t, *J* = 7.4 Hz, 2H), 1.9-1.6 (m, 4H).

^{13}C NMR (101 MHz, $CDCl_3$): δ 200.2, 142.2, 137.1, 132.9, 128.6, 128.4, 128.3, 128.0, 125.7, 38.4, 35.8, 31.1, 24.0 ppm.

5-(4-Methoxyphenyl)-1-phenylpentan-1-one (5ab)[9]

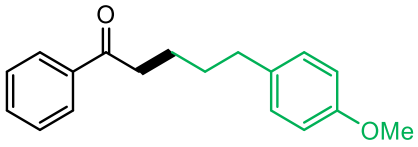

Following the general procedure compound **5ab** was obtained from 3-(4-methoxyphenyl)propyl bromide (57 mg, 0.25 mmol) and 1-azidostyrene (**S1,** 91 mg, 0.625 mmol). The crude product was purified by column chromatography (SiO_2, hexane:diethyl ether, 0-20%) to afford 44 mg, yield = 66%, as colorless solid.

^{1}H NMR (500 MHz, $CDCl_3$): δ 8.0-7.9 (m, 2H), 7.55-7.52 (m, 1H), 7.5-7.4 (m, 2H), 7.09 (d, *J* = 8.6 Hz, 2H), 6.81 (d, *J* = 8.6 Hz, 2H), 3.77 (3 H, s), 2.97 (t, *J* = 7.2 Hz, 2H), 2.61 (t, *J* = 7.5 Hz, 2H), 1.9-1.8 (m, 2H), 1.8-1.6 (m, 2H) ppm. `

^{13}C NMR (126 MHz, $CDCl_3$): δ 200.3, 157.7, 137.1, 134.3, 132.9, 129.2, 128.5, 128.0, 113.7, 55.2, 38.4, 34.8, 31.3, 23.9 ppm.

5-(4-Bromophenyl)-1-phenylpentan-1-one (5ac)[9]

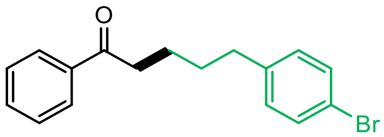

Following the general procedure compound **5ac** was obtained from 1-bromo-4-(3-bromopropyl)benzene (62 mg, 0.22 mmol) and 1-azidostyrene (**S1,** 91 mg, 0.625 mmol). The crude product was purified by column chromatography (SiO_2, hexane:diethyl ether, 0-20%) to afford 44 mg, yield = 77% (contains 5% of 5-phenylvalerophenone), as colorless oil.

^{1}H NMR (500 MHz, $CDCl_3$): δ 8.0-7.9 (m, 2H), 7.6-7.5 (m, 1H), 75-7.43 (m, 2H), 7.38 (d, *J* = 8.3 Hz, 2H), 7.05 (d, *J* = 8.2 Hz, 2H), 2.98 (t, *J* = 7.1, 2H), 2.62 (t, *J* = 7.5 Hz, 2H), 1.8-1.7 (m, 2H), 1.7-1.6 (m, 2H) ppm.

^{13}C NMR (126 MHz, CDCl$_3$): δ 200.1, 141.2, 137.0, 132.9, 131.3, 130.1, 128.6, 128.0, 119.5, 38.3, 35.2, 30.9, 23.8 ppm.

5-(4-*tert*-Butylphenyl)-1-phenylpentan-1-one (5ad)

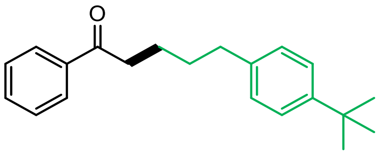

Following the general procedure compound **5ad** was obtained from 4-(3-bromopropyl)-1-(1,1-dimethylethyl)benzene (62 mg, 0.24 mmol) and 1-azidostyrene (**S1,** 91 mg, 0.625 mmol). The crude product was purified by column chromatography (SiO_2, hexane:diethyl ether, 0-20%) to afford 49 mg, yield = 68%, as colorless rigid oil.

^{1}H NMR (500 MHz, CDCl$_3$): δ 8.0-7.9 (m, 2H), 7.6-7.5 (m, 1H), 7.5-7.4 (m, 2H), 7.4-7.2 (m, 2H), 7.2-7.0 (m, 2H), 2.98 (t, J = 7.2 Hz, 2H), 2.64 (t, J = 7.5 Hz, 2H), 1.9-1.7 (m, 2H), 1.8-1.6 (m, 2H), 1.30 (s, 9H) ppm.

^{13}C NMR (126 MHz, CDCl$_3$): δ 200.3, 148.5, 139.14, 137.1, 132.9, 128.5, 128.0, 125.2, 38.4, 35.2, 34.3, 31.4, 31.0, 24.0 ppm.

HRMS (ESI) m/z: [M+Na]$^+$ Calcd for $C_{21}H_{26}ONa$ 317.1889; Found 317.1881.

Anal. Calcd for $C_{21}H_{26}O$: C, 85.67; H: 8.90. Found: C, 85.56; H, 9.07.

Methyl 4-(5-oxo-5-phenylpentyl)benzoate (5ae)[9]

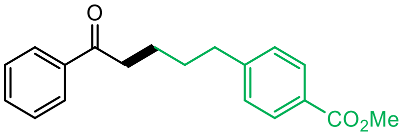

Following the general procedure compound **5ae** was obtained from 4-(3-bromopropyl)-benzoic acid methyl ester (60 mg, 0.23 mmol) and 1-azidostyrene (**S1,** 91 mg, 0.625 mmol). The crude product was purified by column chromatography (SiO_2, hexane:diethyl ether, 0-20%) to afford 51 mg, yield = 74%, as yellowish oil.

^{1}H NMR (500 MHz, CDCl$_3$): δ 8.1-7.8 (m, 4H), 7.6-7.5 (m, 1H), 7.5-7.4 (m, 2H), 7.3-7.2 (m, 2H), 3.89 (s, 3H), 2.98 (t, J = 7.0 Hz, 2H), 2.72 (t, J = 7.4 Hz, 2H), 1.9-1.7 (m, 4H) ppm.

^{13}C NMR (126 MHz, CDCl$_3$): δ 200.0, 167.1, 147.7, 137.0, 132.9, 129.7, 128.5, 128.4, 128.0, 127.8, 51.9, 38.2, 35.8, 30.6, 23.8 ppm.

1,4-Diphenylbutan-1-one (5af)[10]

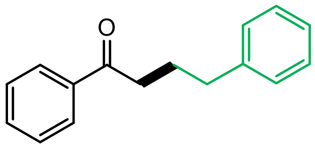

Following the general procedure compound **5af** was obtained from (2-bromoethyl)benzene (48 mg, 0.26 mmol) and 1-azidostyrene (**S1,** 91 mg, 0.625 mmol). The crude product was purified by column chromatography (SiO_2, hexane:diethyl ether, 0-20%) to afford 41 mg, yield = 70%, as colorless oil.

^{1}H NMR (500 MHz, $CDCl_3$): δ 8.0-7.8 (m, 2H), 7.6-7.5 (m, 1H), 7.5-7.4 (m, 2H), 7.30-7.2 (m, 2H), 7.20-7.1 (m, 3H), 2.97 (t, *J* = 7.3 Hz, 2H), 2.72 (t, *J* = 7.6 Hz, 2H), 2.1-2.0 (m, 2H) ppm.

^{13}C NMR (126 MHz, $CDCl_3$): δ 200.1, 141.7, 137.0, 132.9, 128.6, 128.5, 128.4, 128.0, 125.9, 37.7, 35.2, 25.7 ppm.

4-Ethyl-1-phenyloctan-1-one (5ag)[11]

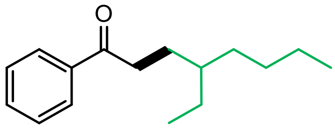

Following the general procedure compound **5ag** was obtained from 2-ethylhexyl bromide (52 mg, 0.27 mmol) and 1-azidostyrene (**S1,** 91 mg, 0.625 mmol). The crude product was purified by column chromatography (SiO_2, hexane:diethyl ether, 0-20%) to afford 17 mg, yield = 30%, as yellow oil.

^{1}H NMR (500 MHz, $CDCl_3$): 7.97 (d, *J* = 7.2 Hz, 2H), 7.56 (t, *J* = 7.4 Hz, 1H), 7.47 (t, *J*=7.6 Hz, 2H), 3.0-2.9 (m, 2H), 1.71 (dd, *J* = 12.8, 8.1 Hz, 2H), 1.4-1.2 (m, 9H), 1.1-0.8 (m, 6H).

^{13}C NMR (126 MHz, $CDCl_3$): δ 200.9, 137.2, 132.8, 128.5, 128.1, 38.7, 36.1, 32.7, 28.9, 27.7, 25.8, 23.09, 23.1, 14.1, 10.82.

1-Phenyl-tetradecan-1-one (5ah)[10]

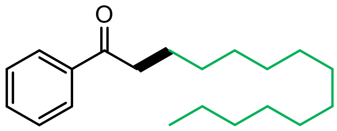

Following the general procedure compound **5ah** was obtained from 1-bromododecane (in DMF: 57 mg, 0.23 mmol; in toluene: 62 mg, 0.25 mmol) and 1-azidostyrene (**S1,** 91 mg, 0.625 mmol). The crude product was purified by column chromatography (SiO_2, hexane:diethyl ether, 0-20%) to afford in DMF 21 mg, yield = 32%; in toluene 39 mg, yield = 55%, as colourless oil.

^{1}H NMR (500 MHz, $CDCl_3$): δ 8.0-7.9 (m, 2H), 7.6-7.5 (m, 1H), 7.5-7.4 (m, 2H), 2.95 (t, *J* = 7.4 Hz, 2H), 1.8-1.6 (m, 2H), 1.5-1.1 (m, 20H), 0.88 (t, *J* = 6.9 Hz, 3H) ppm.

^{13}C NMR (126 MHz, $CDCl_3$): δ 200.6, 137.2, 132.8, 128.5, 128.0, 38.6, 31.9, 29.7, 29.6, 29.6, 29.6, 29.50, 29.5, 29.4, 29.3, 24.4, 22.7, 14.1 ppm.

1-Phenylhept-6-en-1-one (5ai)[12]

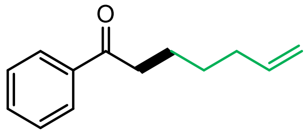

Following the general procedure compound **5ai** was obtained from 5-bromo-1-pentene (37 mg, 0.25 mmol) and 1-azidostyrene (**S1,** 91 mg, 0.625 mmol). The crude product was purified by column chromatography (SiO_2, hexane:diethyl ether, 0-20%) to afford 31 mg, yield = 65%, as yellowish oil.

^{1}H NMR (500 MHz, $CDCl_3$): δ 8.0-7.9 (m, 2H), 7.6-7.5 (m, 1H), 7.5-7.4 (m, 2H), 5.82 (ddt, *J* = 6.7, 10.2, 16.9 Hz, 1H), 5.1-4.9 (m, 2H), 2.97 (t, *J* = 7.4 Hz, 2H), 2.11 (q, *J* = 7.2 Hz, 2H), 1.8-1.7 (m, 2H), 1.5-1.4 (m, 2H) ppm.

^{13}C NMR (126 MHz, $CDCl_3$): δ 200.3, 138.5, 137.1, 132.9, 128.5, 128.0, 114.6, 38.4, 33.5, 28.6, 23.8 ppm.

6-Oxo-6-phenylhexanenitrile (5aj)[12]

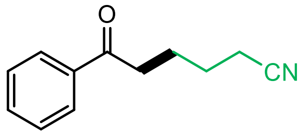

Following the general procedure compound **5aj** was obtained from 4-bromobutanenitrile (39 mg, 0.26 mmol) and 1-azidostyrene (**S1,** 91 mg, 0.625 mmol). The crude product was purified by column chromatography (SiO_2, hexane:diethyl ether, 0-20%) to afford 36 mg, yield = 74%, as colourless solid.

^{1}H NMR (500 MHz, $CDCl_3$): δ 8.0-7.9 (m, 2H), 7.6-7.5 (m, 1H), 7.5-7.4 (m, 2H), 3.04 (t, *J* = 7.0 Hz, 2H), 2.40 (t, *J* = 7.1 Hz, 2H), 2.0-1.8 (m, 2H), 1.8-1.7 (m, 2H) ppm.

^{13}C NMR (126 MHz, $CDCl_3$): δ 199.0, 136.7, 133.1, 128.6, 127.9, 119.4, 37.3, 25.0, 23.0, 17.1 ppm.

Ethyl 6-oxo-6-phenylhexanoate (5ak)[13]

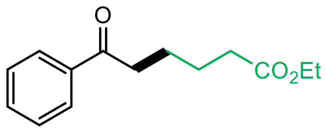

Following the general procedure compound **5ak** was obtained from ethyl 4-bromobutyrate (55 mg, 0.28 mmol) and 1-azidostyrene (**S1,** 91 mg, 0.625 mmol). The crude product was purified by column chromatography (SiO_2, hexane:diethyl ether, 0-20%) to afford 47 mg, yield = 71%, as yellowish oil.

^{1}H NMR (500 MHz, $CDCl_3$): δ 8.0-7.9 (m, 2H), 7.6-7.5 (m, 1H), 7.5-7.4 (m, 2H), 4.13 (q, *J* = 7.1 Hz, 2H), 3.00 (t, *J* = 7.1 Hz, 2H), 2.36 (t, *J* = 7.2 Hz, 2H), 1.8-1.7 (m, 4H), 1.25 (t, *J* = 7.1 Hz, 3H) ppm.

^{13}C NMR (126 MHz, $CDCl_3$): δ 199.8, 173.4, 136.9, 132.9, 128.5, 128.0, 60.2, 38.1, 34.1, 24.6, 23.6, 14.2 ppm.

2-(5-Oxo-5-phenylpentyl)isoindole-1,3-dione (5al)[14]

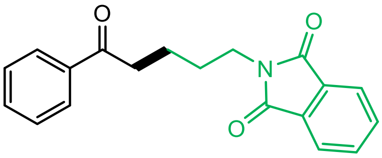

Following the general procedure compound **5al** was obtained from *N*-(3-bromopropyl)phthalimide (63 mg, 0.24 mmol) and 1-azidostyrene (**S1,** 91 mg, 0.625 mmol). The crude product was purified by column chromatography (SiO_2, hexane:diethyl ether, 0-20%) to afford 47 mg, yield = 65%, yellowish solid.

^{1}H NMR (500 MHz, $CDCl_3$): δ 8.0-7.9 (m, 2H), 7.9-7.8 (m, 2H), 7.8-7.6 (m, 2H), 7.6-7.5 (m, 1H), 7.4-7.4 (m, 2H), 3.8-3.7 (m, 2H), 3.1-3.0 (m, 2H), 1.8-1.7 (m, 4H) ppm.

^{13}C NMR (126 MHz, $CDCl_3$): δ 199.7, 168.4, 136.9, 133.9, 133.0, 132.1, 128.6, 128.0, 123.2, 37.7, 37.6, 28.1, 21.3 ppm.

1-Phenyl-3-(1-tosylpyrrolidin-3-yl)propan-1-one (5am)

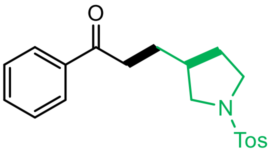

Following changed general procedure compound **5am** was obtained from *N*-(2-bromoethyl)-*N*-(2-propenyl)-4-methylbenzenesulfonamide (78 mg, 0.25 mmol) and 1-azidostyrene (**S1,** 91 mg, 0.625 mmol) by irradiating with 10 W single blue LED light for 30 min. The crude product was purified by column chromatography (SiO_2, hexane:diethyl ether, 0-20%) to afford 31 mg, yield = 36%, as yellowish powder.

^{1}H NMR (500 MHz, $CDCl_3$): δ 7.91 (d, *J* = 7.7 Hz, 2H), 7.70 (d, *J* = 7.9 Hz, 2H), 7.57 (t, *J* = 7.5 Hz, 1H), 7.46 (t, *J* = 7.7 Hz, 2H), 7.31 (s, 1H), 7.26 (s, 1H), 3.48 (dd, *J* = 9.8, 7.3 Hz, 1H), 3.36 (td, *J* = 9.1, 3.6 Hz, 1H), 3.21 (q, *J* = 8.6 Hz, 1H), 2.92 (t, *J* = 7.4 Hz, 2H), 2.84 (t, *J* = 8.9 Hz, 1H), 2.42 (s, 3H), 2.12 (s, 1H), 1.97 (q, *J* = 4.8 Hz, 1H), 1.70 (qq, *J* = 14.4, 7.3 Hz, 2H), 1.5-1.4 (m, 1H) ppm.

^{13}C NMR (126 MHz, $CDCl_3$): δ 199.2, 143.4, 136.7, 133.8, 133.2, 129.6, 128.7, 127.9, 127.5, 53.1, 47.5, 38.3, 36.6, 31.4, 27.2, 21.5 ppm.

HRMS (ESI) m/z: [M+Na]$^+$ Calcd for $C_{20}H_{23}NO_3SNa$ 380.1296; Found 380.1290.

Anal. Calcd for $C_{20}H_{23}NO_3S$: C, 67.20; H, 6.49; N, 3.92. Found: C, 67.21; H, 6.57; N, 4.03.

4-(Benzyloxy)-1-phenylbutan-1-one (5an)[15]

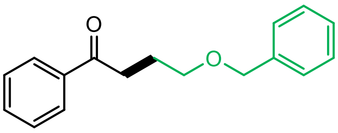

Following the general procedure compound **5an** was obtained from benzyl 2-bromoethyl ether (61 mg, 0.28 mmol) and 1-azidostyrene (**S1,** 91 mg, 0.625 mmol). The crude product was purified by column chromatography (SiO_2, hexane:diethyl ether, 0-20%) to afford 54 mg, yield = 74%, as yellowish oil.

^{1}H NMR (500 MHz, $CDCl_3$): δ 8.1-7.9 (m, 2H), 7.6-7.5 (m, 1H), 7.5-7.4 (m, 2H), 7.4-7.3 (m, 3H), 7.3-7.2 (m, 2H), 4.50 (s, 2H), 3.57 (t, *J* = 6.1 Hz, 2H), 3.10 (t, *J* = 7.2 Hz, 2H), 2.1-2.0 (m, 2H) ppm.

^{13}C NMR (126 MHz, $CDCl_3$): δ 199.9, 138.4, 137.0, 132.9, 128.5, 128.3, 128.0, 127.6, 127.5, 72.9, 69.4, 35.1, 24.3 ppm.

10-Hydroxydecanophenone (5ao)[16]

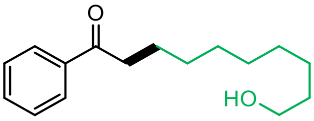

Following the general procedure compound **5ao** was obtained from 8-bromo-1-octanol (55 mg, 0.26 mmol) and 1-azidostyrene (**S1,** 91 mg, 0.625 mmol). The crude product was purified by column chromatography (SiO_2, hexane:diethyl ether, 0-20%) to afford 56 mg, yield = 85%, as colourless solid.

^{1}H NMR (500 MHz, $CDCl_3$): δ 7.96 (d, *J*=7.4 Hz, 2H), 7.6-7.5 (m, 1H), 7.5-7.4 (m, 2H), 3.7-3.6 (m, 2H), 2.96 (t, *J* = 7.4 Hz, 2H), 1.8-1.7 (m, 2H), 1.6-1.5 (m, 2H), 1.4-1.3 (m, 11 H) ppm.

^{13}C NMR (126 MHz, $CDCl_3$): δ 200.6, 137.1, 132.8, 128.5, 128.0, 63.0, 38.6, 32.8, 29.40, 29.36, 29.33, 29.32, 25.7, 24.3 ppm.

4-(2-(2-Methoxyethoxy)ethoxy)-1-phenylbutan-1-one (5ap)

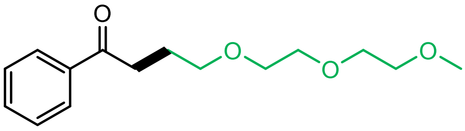

Following the general procedure compound **5ap** was obtained from 3,6,9-trioxadecyl bromide (64 mg, 0.28 mmol) and 1-azidostyrene (**S1,** 91 mg, 0.625 mmol). The crude product was purified by column chromatography (SiO_2, hexane:diethyl ether, 0-20%) to afford 43 mg, yield = 58%, as colourless oil.

^{1}H NMR (500 MHz, $CDCl_3$): δ 8.1-7.8 (m, 2H), 7.6-7.5 (m, 1H), 7.5-7.4 (m, 2H), 3.7-3.5 (m, 10H), 3.36 (s, 3H), 3.08 (t, *J* = 7.2Hz, 2H), 2.03 (p, *J* = 6.6 Hz, 2H) ppm.

^{13}C NMR (126 MHz, $CDCl_3$): δ 200.00, 137.1, 132.9, 128.5, 128.0, 72.0, 70.6, 70.5, 70.3, 70.0, 59.0, 35.1, 24.2 ppm.

HRMS (ESI) m/z: $[M+Na]^+$ calculated for $C_{15}H_{22}O_4Na$: 284.1418, found: 284.1416.

GC chromatogram (>98% purity)

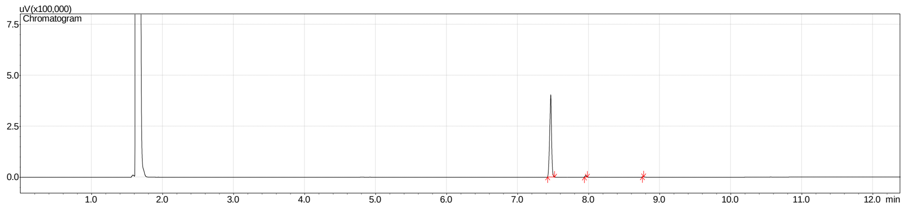

1-(4-(*tert*-Butyl)phenyl)-5-phenylpentan-1-one (5oa)[17]

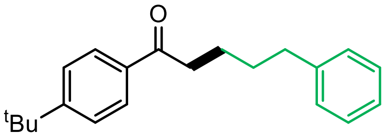

Following the general procedure compound **5oa** was obtained from 1-bromo-3-phenylpropane (**S2,** 55 mg, 0.28 mmol) and 1-(1-azidovinyl)-4-(tert-butyl)benzene (126 mg, 0.626 mmol). The crude product was purified by column chromatography (SiO_2, hexane:diethyl ether, 0-20%) to afford 55 mg, yield = 69%, as colourless rigid oil.

^{1}H NMR (500 MHz, $CDCl_3$): δ 8.0-7.8 (m, 2H), 7.5-7.4 (m, 2H), 7.3-7.2 (m, 2H), 7.2-7.1 (m, 3H), 2.97 (t, *J* = 7.2 Hz, 2H), 2.67 (t, *J* = 7.5 Hz, 2H), 1.9-1.7 (m, 4H), 1.35 (s, 9H) ppm.

^{13}C NMR (126 MHz, $CDCl_3$): δ 199.9, 156.6, 142.3, 134.5, 128.4, 128.3, 128.0, 125.7, 125.5, 38.3, 35.8, 35.1, 31.1, 31.08, 24.1 ppm.

1-(Naphthalen-2-yl)-5-phenylpentan-1-one (5pa)[18]

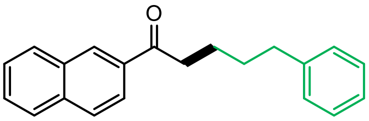

Following the general procedure compound **5pa** was obtained from 1-bromo-3-phenylpropane (**S2,** 52 mg, 0.26 mmol) and 2-(1-azidovinyl)naphthalene (122 mg, 0.625 mmol). The crude product was purified by column chromatography (SiO_2, hexane:diethyl ether, 0-20%) to afford 48 mg, yield = 63%, as colourless rigid oil.

^{1}H NMR (400 MHz, $CDCl_3$): δ 8.43 (s, 1H), 8.01 (dd, *J* = 8.6, 1.7 Hz, 1H), 8.0-7.9 (m, 1H), 7.9-7.8 (m, 2H), 7.6-7.5 (m, 2H), 7.3-7.1 (m, 4H), 3.10 (t, *J* = 7.2 Hz, 2H), 2.69 (t, *J* = 7.5 Hz, 2H), 1.9-1.7 (m, 4H) ppm.

^{13}C NMR (101 MHz, $CDCl_3$): δ 200.2, 142.3, 135.6, 134.4, 132.6, 129.6, 129.6, 128.4, 128.3, 127.8, 126.7, 125.8, 124.0, 38.5, 35.8, 31.1, 24.2 ppm.

1-((1,1'-Biphenyl)-4-yl)-5-phenylpentan-1-one (5qa)[18]

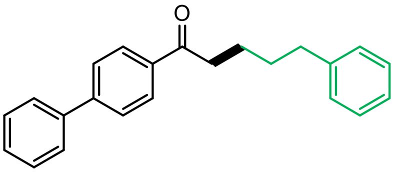

Following the general procedure compound **5qa** was obtained from 1-bromo-3-phenylpropane (**S2,** 55 mg, 0.28 mmol) and 4-(1-azidovinyl)-1,1'-biphenyl (138 mg, 0.624 mmol). The crude product was purified by column chromatography (SiO_2, hexane:diethyl ether, 0-20%) to afford 28 mg, yield = 32%, as yellowish solid

^{1}H NMR (500 MHz, $CDCl_3$): δ 8.01 (d, *J* = 8.3 Hz, 2H), 7.67 (d, *J* = 8.3 Hz, 2H), 7.62 (d, *J* = 7.4 Hz, 2H), 7.46 (t, *J* = 7.5 Hz, 2H), 7.39 (t, *J* = 7.3 Hz, 1H), 7.28 (t, *J* = 7. 68 (t, 5 Hz, 2H), 7.2-7.1 (m, 3H), 3.01 (t, *J* = 7.2 Hz, 2H), 2. *J* = 7.5 Hz, 2H), 1.9-1.8 (m, 2H), 1.8-1.7 (m, 2H) ppm.

^{13}C NMR (126 MHz, $CDCl_3$): δ 199.8, 145.6, 142.2, 139.9, 135.7, 128.9, 128.6, 128.4, 128.3, 128.2, 127.2, 127.2, 125.7, 38.5, 35.8, 31.1, 24.1 ppm.

1-(4-Chlorophenyl)-5-phenylpentan-1-one (5ra)

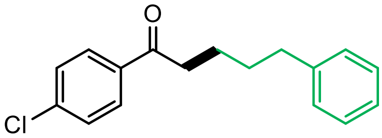

Following the general procedure compound **5ra** was obtained from 1-bromo-3-phenylpropane (**S2,** 52 mg, 0.26 mmol) and 1-(1-azidovinyl)-4-chlorobenzene (112 mg, 0.624 mmol). The crude product was purified by column chromatography (SiO_2, hexane:diethyl ether, 0-20%) to afford 31 mg, yield = 43%, as colourless oil.

^{1}H NMR (400 MHz, $CDCl_3$): δ 8.0-7.8 (m, 2H), 7.5-7.4 (m, 2H), 7.4-7.2 (m, 2H), 7.2-7.1 (m, 3H), 2.97 (t, *J* = 7.1 Hz, 2H), 2.69 (t, *J* = 7.4 Hz, 2H), 1.9-1.7 (m, 4H) ppm.

^{13}C NMR (101 MHz, $CDCl_3$): δ 198.9, 142.1, 139.3, 135.4, 129.5, 128.9, 128.4, 128.3, 125.8, 38.4, 35.8, 31.0, 23.9 ppm.

HRMS (EI+) m/z: $[M]^+$ Calcd for $C_{17}H_{17}ClO$ 272.0968; Found 272.0965.

Anal. Calcd for $C_{17}H_{17}ClO$: C, 74.86; H, 6.28. Found: C, 74.72; H, 6.29.

1-(4-Methoxyphenyl)-5-phenylpentan-1-one (5sa)[18]

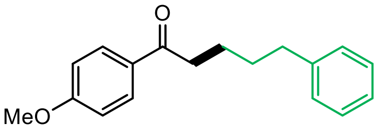

Following the general procedure compound **5sa** was obtained from 1-bromo-3-phenylpropane (**S2,** 53 mg, 0.27 mmol) and 1-(1-azidovinyl)-4-methoxybenzene (109 mg, 0.622 mmol). The crude product was purified by column chromatography (SiO_2, hexane:diethyl ether, 0-20%) to afford 39 mg, yield = 53%, as yellowish oil.

^{1}H NMR (500 MHz, $CDCl_3$): δ 8.0-7.9 (m, 2H), 7.3-7.2 (m, 2H), 7.2-7.1 (m, 3H), 7.0-6.9 (m, 2H), 3.85 (s, 3H), 2.92 (t, *J* = 7.2 Hz, 2H), 2.66 (t, *J* = 7.5 Hz, 2H), 1.8-1.6 (m, 4H) ppm.

^{13}C NMR (126 MHz, $CDCl_3$): δ 198.8, 163.3, 142.3, 130.3, 130.1, 128.4, 128.3, 125.7, 113.7, 55.4, 38.1, 35.8, 31.2, 24.2 ppm.

1-(3-Methoxyphenyl)-5-phenylpentan-1-one (5sb)

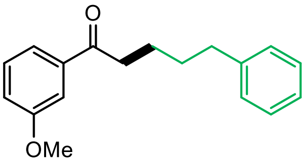

Following the general procedure compound **5sb** was obtained from 1-bromo-3-phenylpropane (50 mg, 0.25 mmol) and 1-(1-azidovinyl)-3-methoxybenzene (109 mg, 0.622 mmol). The crude product was purified by column chromatography (SiO_2, hexane:diethyl ether, 0-20%) to afford 33 mg, yield = 43%, as colourless rigid oil.

^{1}H NMR (500 MHz, $CDCl_3$): δ 7.6-7.5 (m, 2H), 7.38 (t, *J* = 7.9 Hz, 1H), 7.3-7.2 (m, 2H), 7.2-7.1 (m, 2H), 7.12 (ddd, *J*= 8.2, 2.6, 0.8 Hz, 1H), 3.88 (s, 3H), 2.99 (t, *J* = 7.1 Hz, 2H), 2.70 (t, *J* = 7.4 Hz, 2H), 1.9-1.7 (m, 4H) ppm.

^{13}C NMR (126 MHz, CDCl$_3$): δ 200.0, 159.9, 142.2, 138.5, 129.5, 128.4, 128.3, 125.7, 120.7, 119.3, 112.36, 55.4, 38.5, 35.8, 31.1, 24.1 ppm.

HRMS (ESI) m/z: [M+Na]$^+$ Calcd for $C_{18}H_{20}O_2Na$ 291.1361; Found 291.1357.

Anal. Calcd for $C_{18}H_{20}O_2$: C, 80.56; H, 7.51. Found: C, 80.51; H, 7.55.

1-Cyclohexenyl-5-phenylpentan-1-one (5ta)

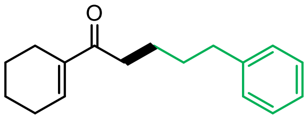

Following the general procedure compound **5ta** was obtained from 1-bromo-3-phenylpropane (**S2,** 50 mg, 0.25 mmol) and 1-(1-azidovinyl)cyclohex-1-ene (93 mg, 0.623 mmol). The crude product was purified by column chromatography (SiO_2, hexane:diethyl ether, 0-20%) to afford 33 mg, yield = 55%, as colourless oil.

^{1}H NMR (400 MHz, CDCl$_3$): δ 7.3-7.2 (m, 2H), 7.2-7.1 (m, 3H), 6.9-6.8 (m, 1H), 2.7-2.6 (m, 4H), 2.3-2.2 (m, 4H), 1.7-1.6 (m, 8H) ppm.

^{13}C NMR (101 MHz, CDCl$_3$): δ 201.4, 142.4, 139.5, 139.3, 128.4, 128.3, 125.7, 36.8, 35.8, 31.2, 26.0, 24.5, 23.2, 22.0, 21.6 ppm.

HRMS (ESI) m/z: [M+Na]$^+$ Calcd for $C_{17}H_{22}ONa$ 265.1568; Found 265.1555.

Anal. Calcd for $C_{17}H_{22}O$: C, 84.25; H, 9.15. Found: C, 84.05; H, 9.23..

1-Cyclohexenyl-5-(4-(tert-butyl)phenyl)pentan-1-one (5tb)

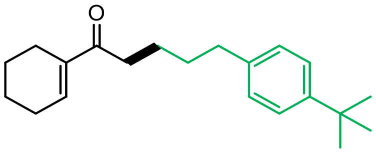

Following the general procedure compound **5tb** was obtained from 4-(3-bromopropyl)-1-(1,1-dimethylethyl)benzene (64 mg, 0.25 mmol) and 1-(1-azidovinyl)cyclohex-1-ene (93 mg, 0.623 mmol). The crude product was purified by column chromatography (SiO_2, hexane:diethyl ether, 0-20%) to afford 40 mg, yield = 53%, as colourless oil.

^{1}H NMR (400 MHz, CDCl$_3$): δ 7.29 (d, J = 3.8 Hz, 2H), 7.11 (d, J = 3.8 Hz, 2H), 6.9-6.8 (m, 1H), 2.7-2.6 (m, 4H), 2.3-2.2 (m, 4H), 1.7-1.6 (m, 8H), 1.31 (s, 9H) ppm.

^{13}C NMR (101 MHz, CDCl$_3$): δ 201.5, 148.4, 139.4, 139.3, 128.0, 125.4, 125.1, 36.9, 35.2, 34.3, 31.4, 31.1, 26.0, 24.6, 23.2, 22.0, 21.6 ppm.

HRMS (ESI) m/z: [M+Na]$^+$ Calcd for $C_{21}H_{30}ONa$ 321.2187; Found 321.2194.

Anal. Calcd for $C_{21}H_{30}O$: C, 84.51; H, 10.13. Found: C, 84.34; H, 10.13.

Methyl 5-oxo-9-phenyl-nonanoate (5ua)

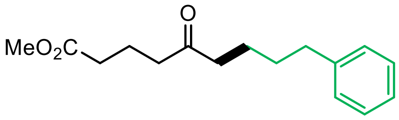

Following the general procedure compound **5ua** was obtained from 1-bromo-3-phenylpropane (**S2,** 50 mg, 0.25 mmol) and methyl 5-azidohex-5-enoate (106 mg, 0.627 mmol). The crude product was purified by column chromatography (SiO_2, hexane:diethyl ether, 0-20%) to afford 18 mg, yield = 27%, as colourless oil.

^{1}H NMR (400 MHz, $CDCl_3$): δ 7.3-7.2 (m, 2H), 7.2-7.1 (m, 3H), 3.66 (s, 3H), 2.7-2.6 (m, 2H), 2.5-2.4 (m, 4H), 2.4-2.3 (m, 2H), 1.9-1.8 (m, 2H), 1.7-1.6 (m, 4H) ppm.

^{13}C NMR (101 MHz, $CDCl_3$): δ 210.0, 173.6, 142.2, 128.4, 128.3, 125.7, 51.5, 42.6, 41.5, 35.7, 33.1, 31.0, 23.4, 18.9 ppm.

HRMS (ESI) m/z: $[M+Na]^+$ Calcd for $C_{16}H_{23}ONa$ 285.1467; Found 285.1458.

Anal. Calcd for $C_{16}H_{23}O$: C, 73.25; H, 8.45. Found: C, 73.04; H, 8.45.

7. Literature

1. Donthiri, R. R., Pappula, V., Reddy, N. N. K., Bairagi, D. Adimurthy, S. *J. Org. Chem.* **2014**, 79, 22, 11277–11284
2. Liu, Z., Liao, P., Bi, X. *Org. Lett.* **2014**, 16, 14, 3668–3671
3. Do, Q., Nguyen, G. T., Phillips, R. S. *Amino Acids* **2016, **48**,** 2243–2251
4. Molander, G. A., Argintaru, O. A., Aron, I., Dreher, S. D. *Org. Lett.* **2010**, 12, 24, 5783–5785
5. Herold, S., Bafaluy, D., Muñiz, K. *Green Chem*. **2018**, 20, 3191-3196
6. Varney, M. D., Palmer, C. L., Romines, W. H., Boritzki, T., Margosiak, S. A., Almassy, R., Janson, C. A., Bartlett, C., Howland, E. J., Ferre, R. *J. Med. Chem.* **1997**, 40, 16, 2502–2524
7. Smoleń, S., Wincenciuk, A., Drapała, O., Gryko, D. *Synthesis* **2021**, 53(09), 1645-1653
8. Sergeev, M. E., Morgia, F., Lazari, M., Wang Jr. C., van Dam, R. M. *J. Am. Chem. Soc*. **2015**, 137, 17, 5686–5694
9. Sakurai, S., Tsuzuki, S., Sakamoto, R., Maruoka, K. *J. Org. Chem.* **2020**, *85, 5, 3973–3980*
10. Wang, J., Pang, Y., Tao, N., Zeng, R., Zhao, Y. *J. Org. Chem.* **2019**, 84, 23, 15315–15322
11. Wang, D., Zhao, K., Ma, P., Xu, C., Ding, Y. *Tet. Let.* **2014**, 55, 7233–7235
12. Ren, R., Wu, Z., Xu, Y., Zhu, C. *Angew. Chem. Int. Ed.* **2016**, *55*, 2866–2869
13. Xie, J., Li, J., Weingand, V., Rudolph, M., Hashmi, A. S. K. *Chem. Eur. J.* **2016**, *22*, 12646
14. Kanai, Y., Nagamori, S., Kitaura, Y., Neya, M., Matsushita, N. *US2015/336876*, **2015**, A1
15. Dhokte, U. P., Rao, A. S. *Org. Prep. Proced. Int.*, **1992**, 24:1, 13-20
16. Sui, G., Lv, Q., Song, X., Guo, H., Dai, J., Ren, L., Lee, C., Zhou, W., Hao, H. *New J. Chem.*, **2019**, 43, 15793-15796
17. Xia, Z., Zhang, C., Gao, Z., Ye, S. *Org. Lett.* **2018**, 20, 12, 3496–3499
18. Zheng, Y., Xie, P., Daneshfar, O., Houk, K. N., Hong, X., Newman, S. G. *Angew.Chem. Int. Ed.* **2021**, 60, 13476–13483

8. ^{1}H NMR and ^{13}C NMR spectra

1,5-diphenylpentan-1-one (5aa) ^{1}H NMR (400 MHz, $CDCl_3$) and ^{13}C NMR (100 MHz, $CDCl_3$)

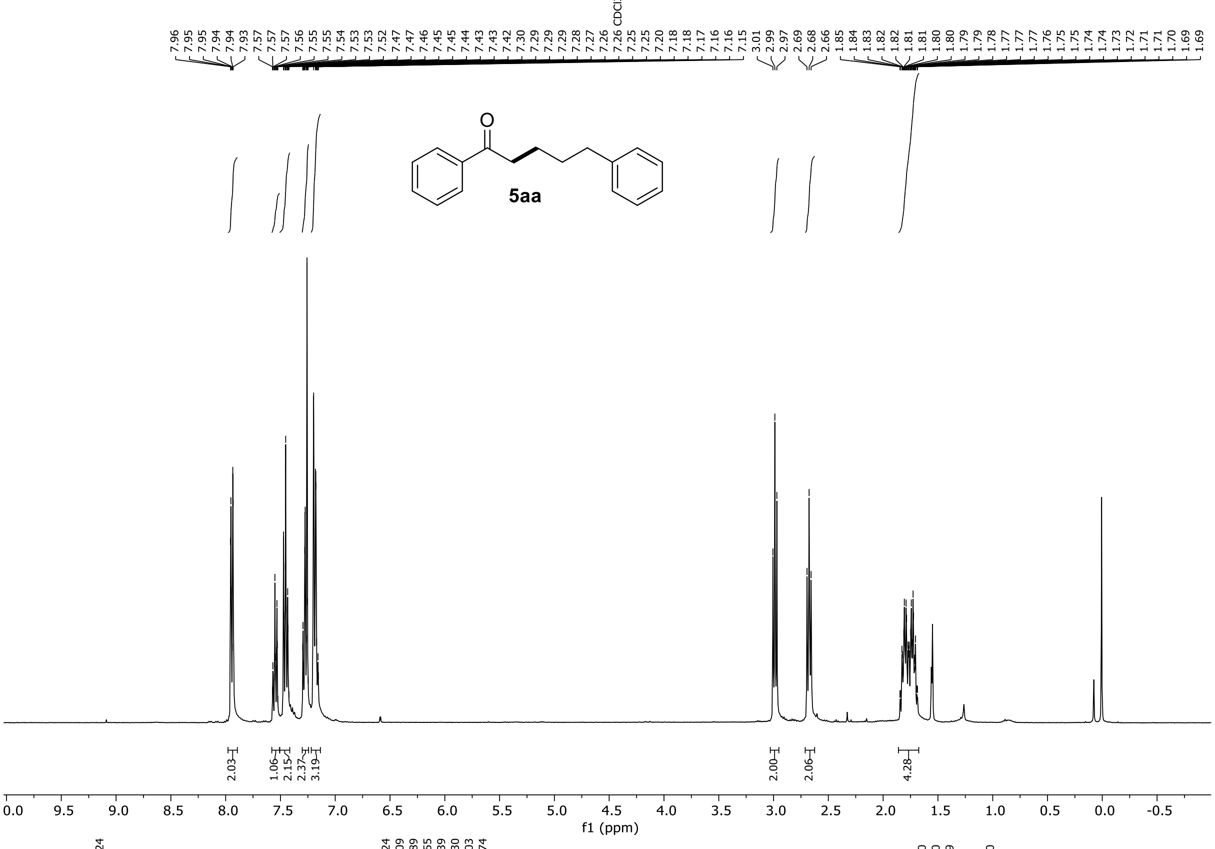

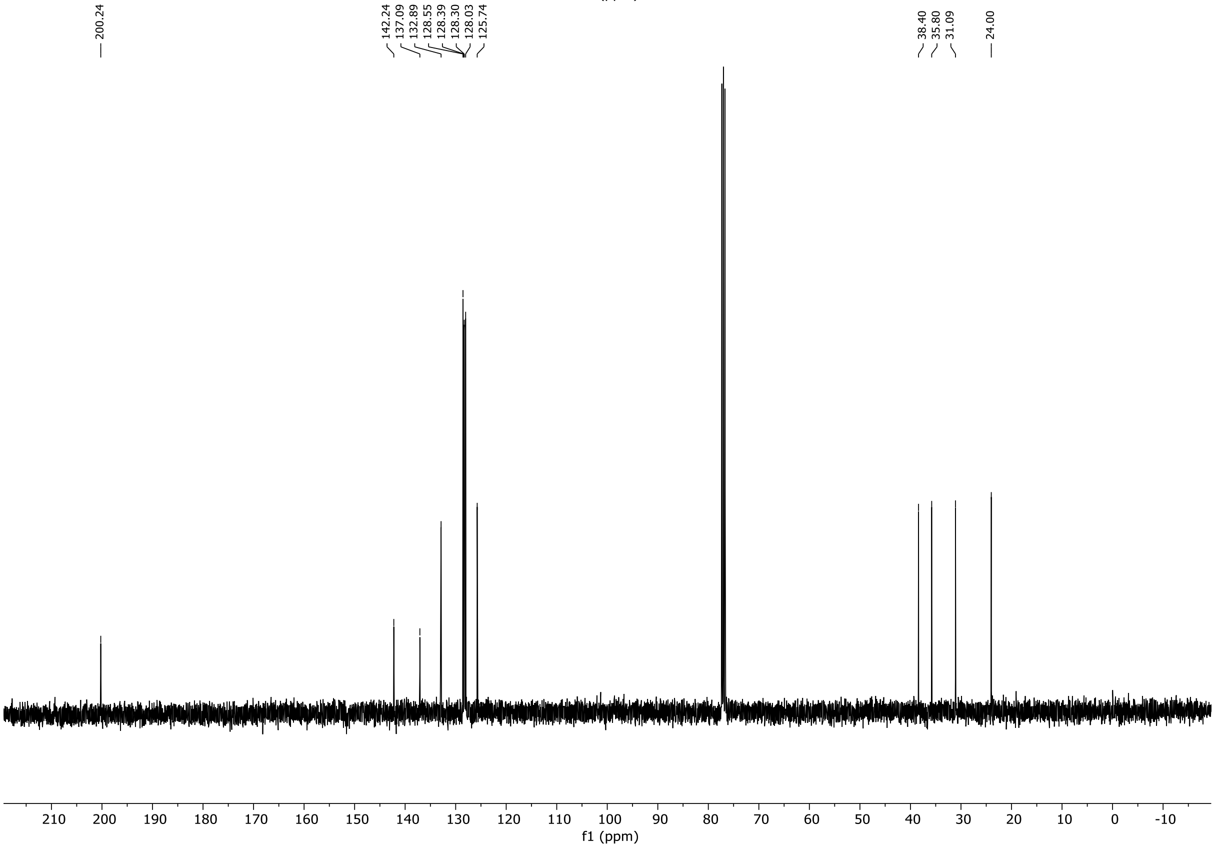

5-(4-methoxyphenyl)-1-phenylpentan-1-one (5ab) ^{1}H NMR (500 MHz, $CDCl_3$) and ^{13}C NMR (126 MHz, $CDCl_3$)

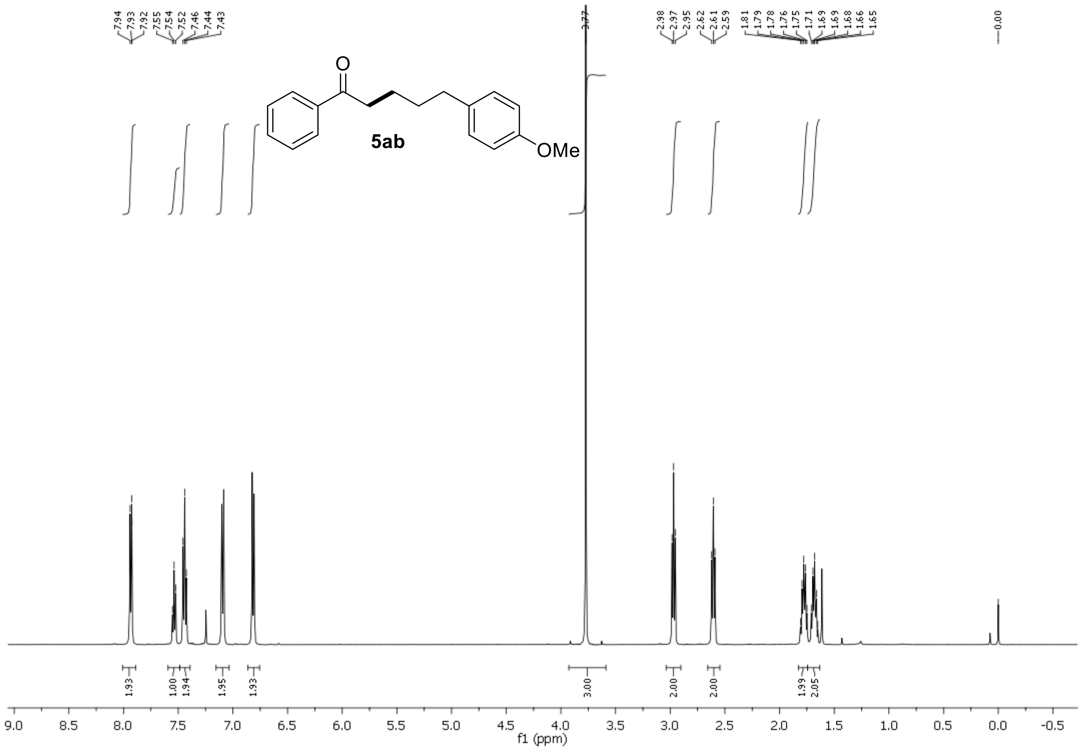

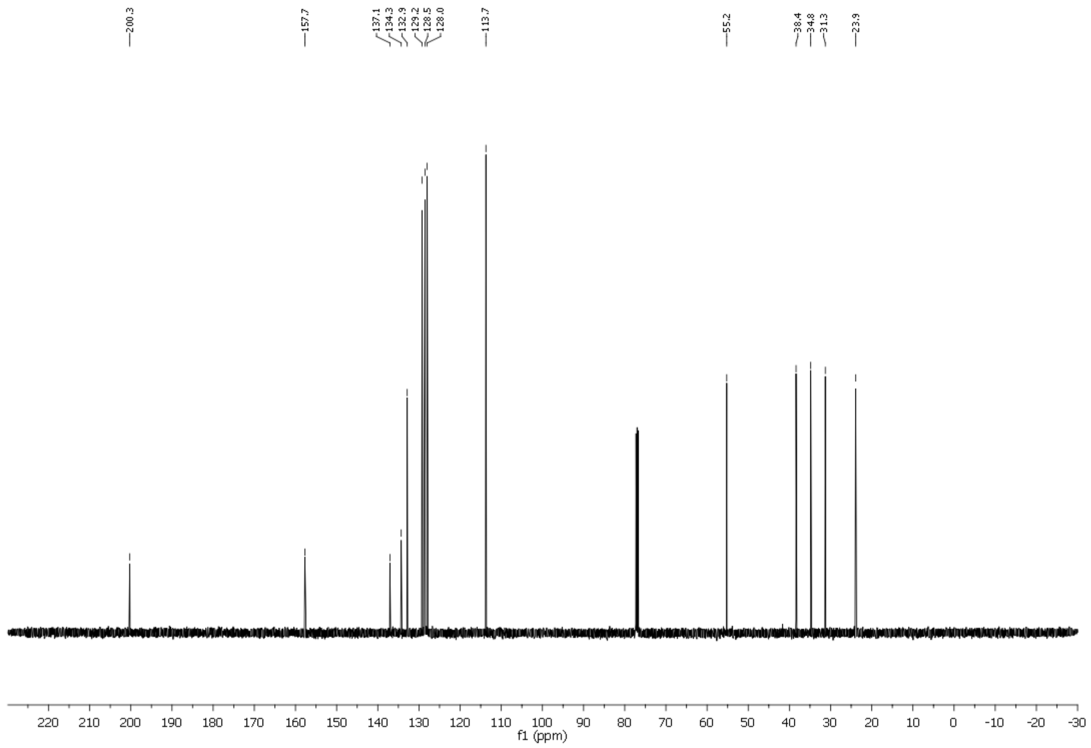

5-(4-bromophenyl)-1-phenylpentan-1-one (5ac) ^{1}H NMR (500 MHz, $CDCl_3$) and ^{13}C NMR (126 MHz, $CDCl_3$)

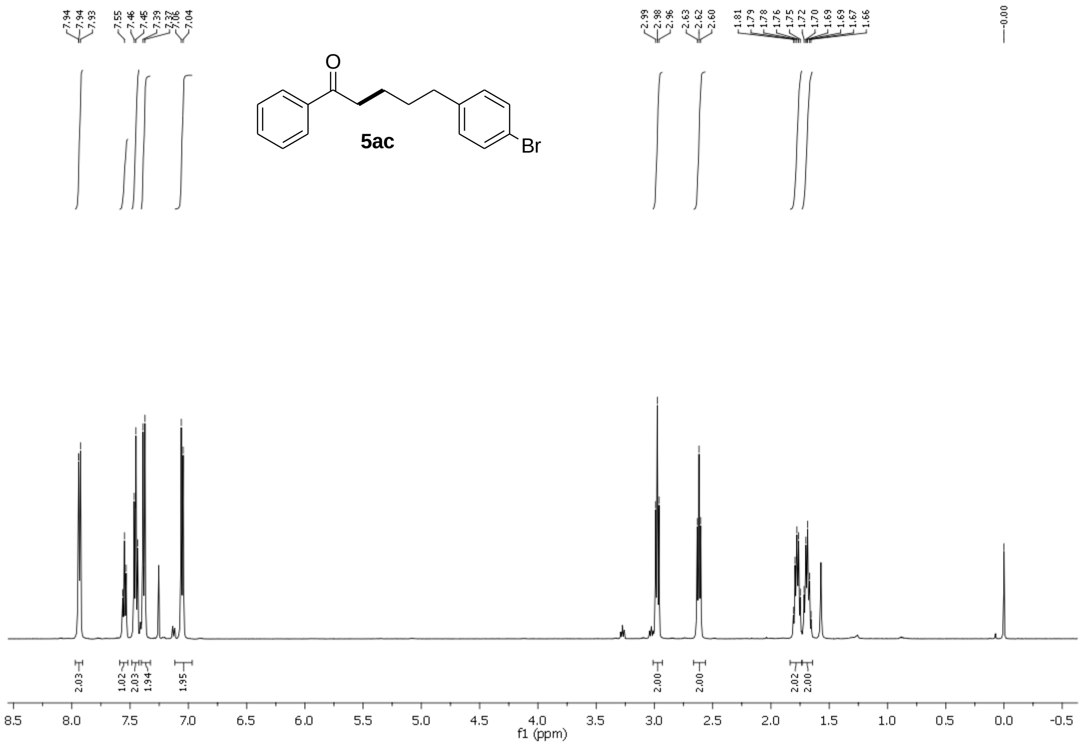

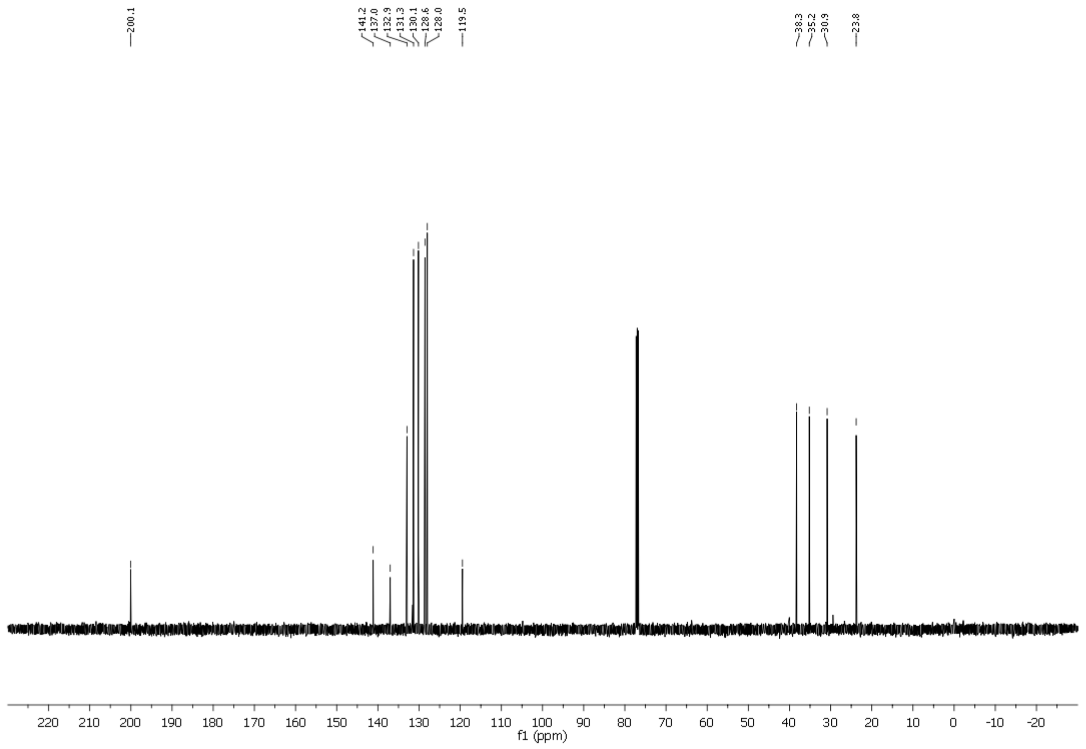

5-(4-tert-butylphenyl)-1-phenylpentan-1-one (5ad) ^{1}H NMR (500 MHz, $CDCl_3$) and ^{13}C NMR (126 MHz, $CDCl_3$)

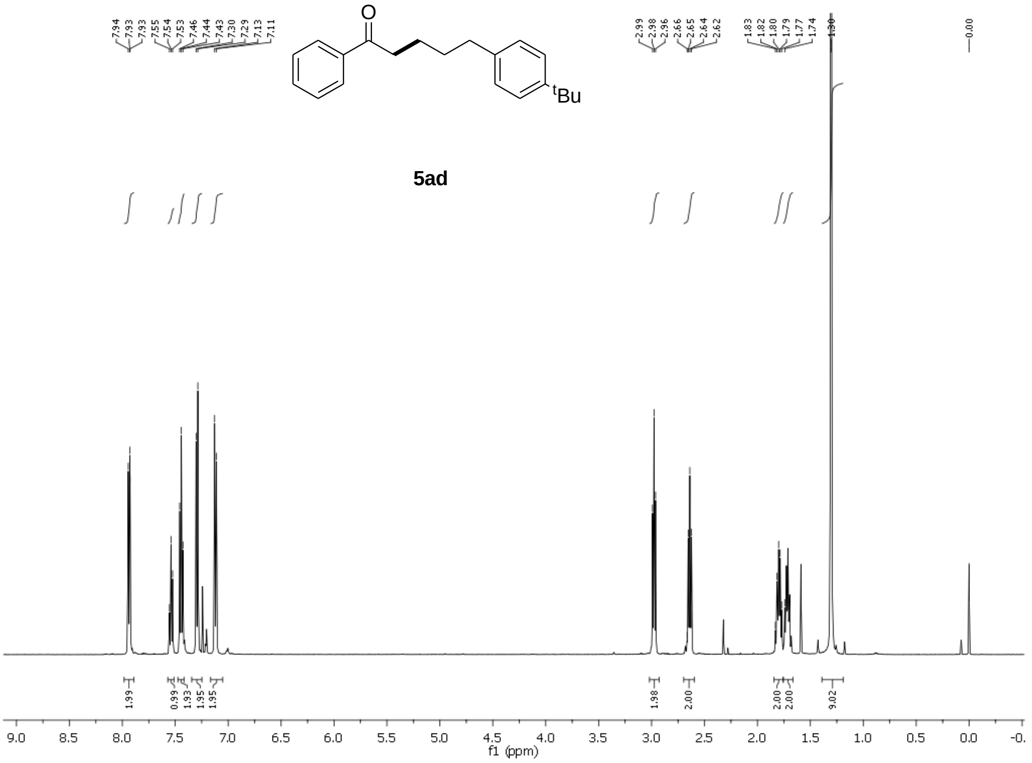

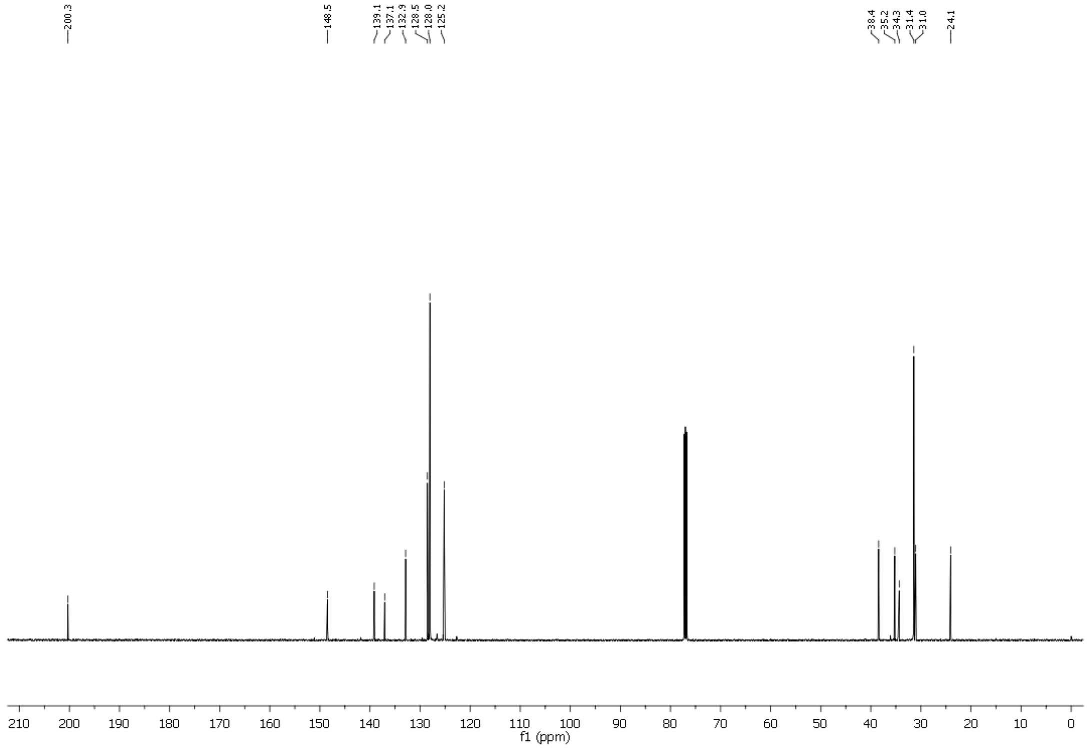

methyl 4-(5-oxo-5-phenylpentyl)benzoate (5ae) ^{1}H NMR (500 MHz, $CDCl_3$) and ^{13}C NMR (126 MHz, $CDCl_3$)

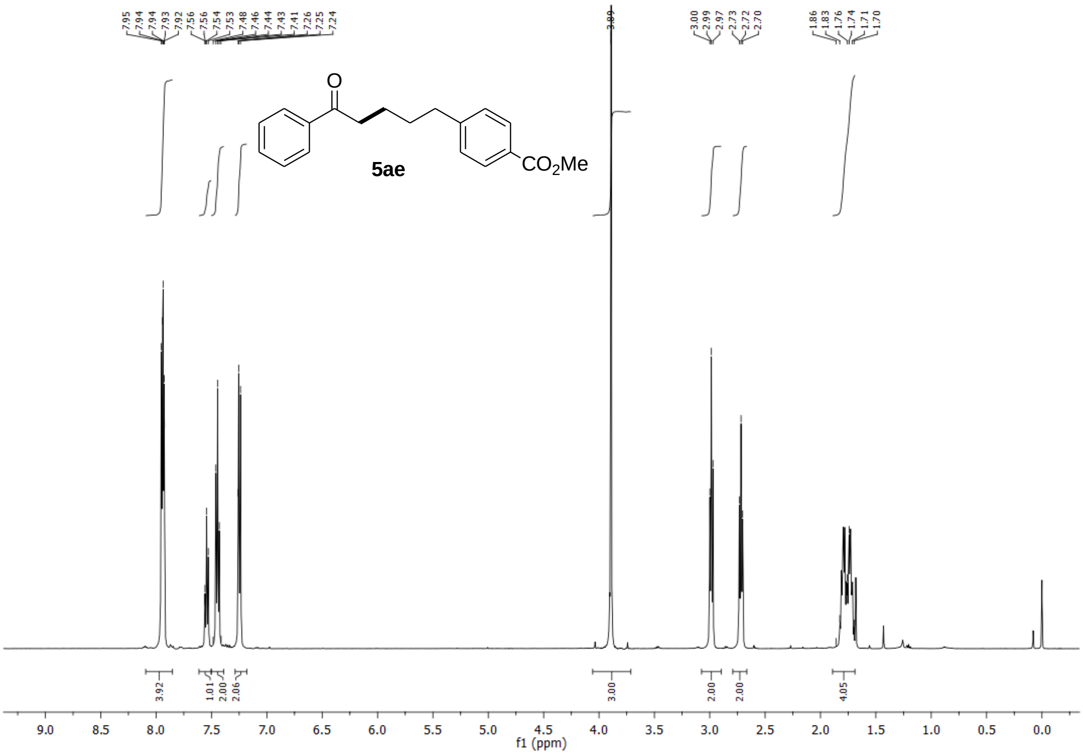

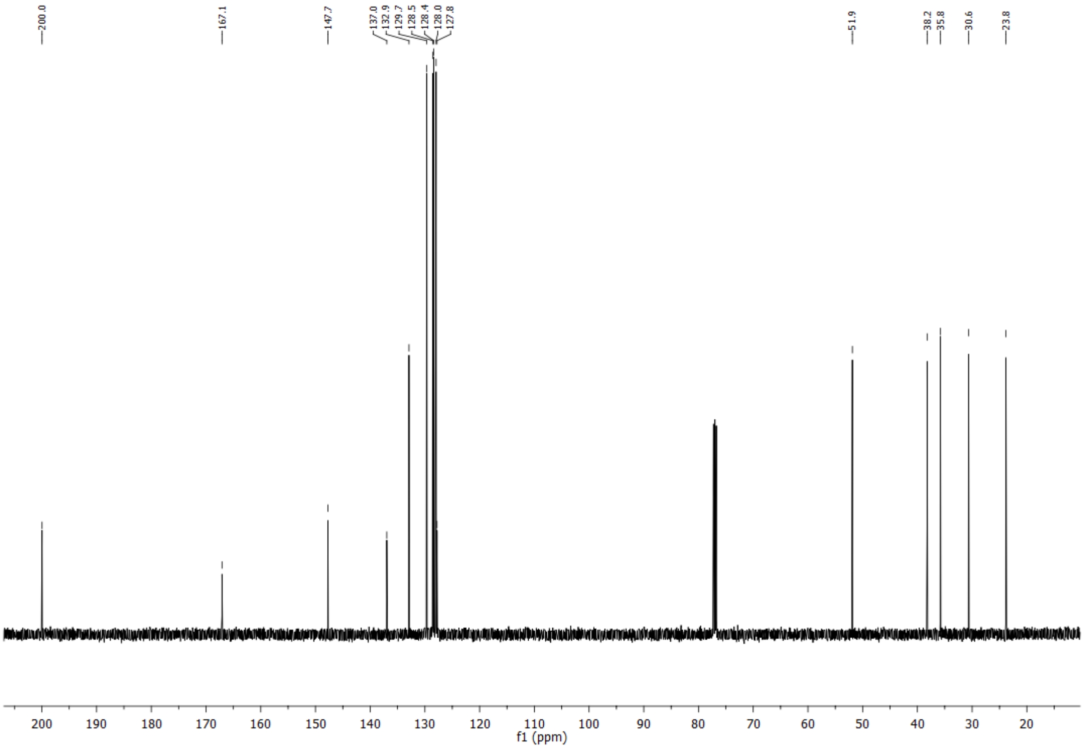

1,4-diphenylbutan-1-one (5af) ^{1}H NMR (500 MHz, $CDCl_3$) and ^{13}C NMR (126 MHz, $CDCl_3$)

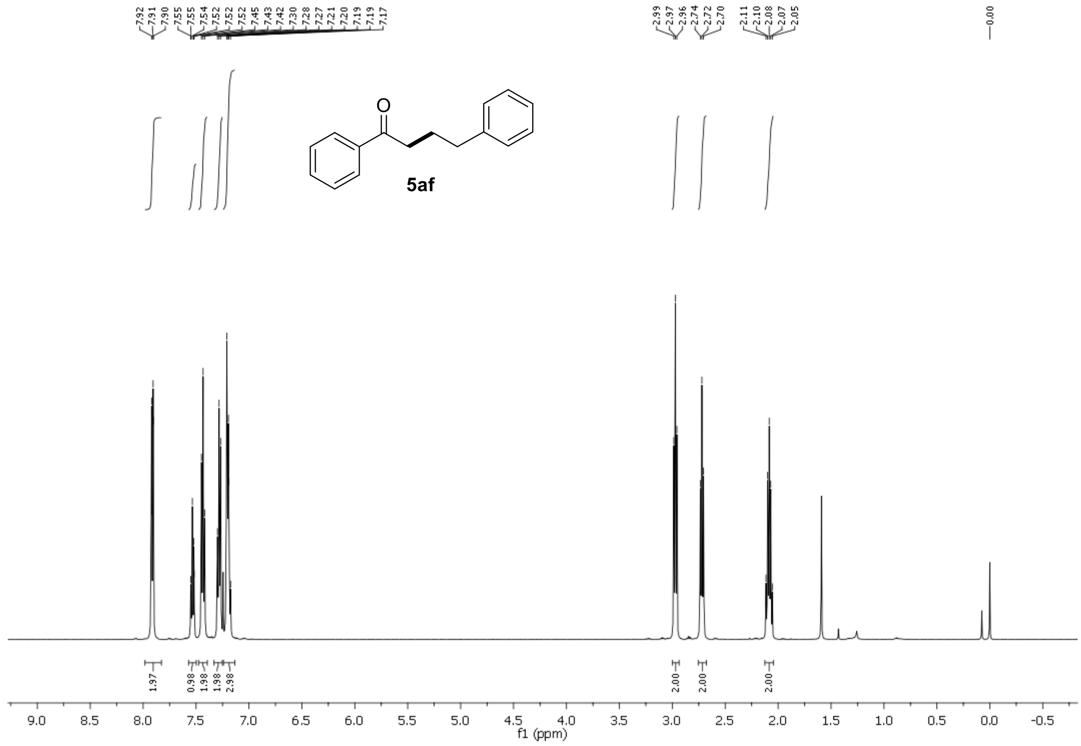

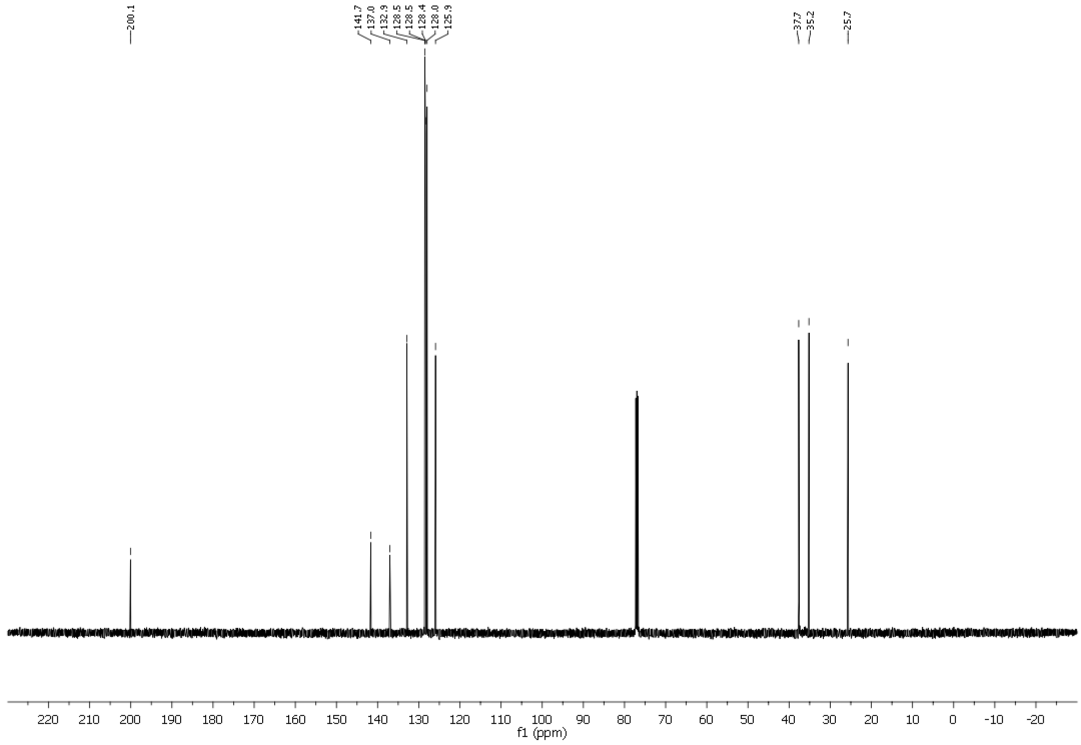

4-ethyl-1-phenyloctan-1-one (5ag) ^{1}H NMR (500 MHz, $CDCl_3$) and ^{13}C NMR (126 MHz, $CDCl_3$)

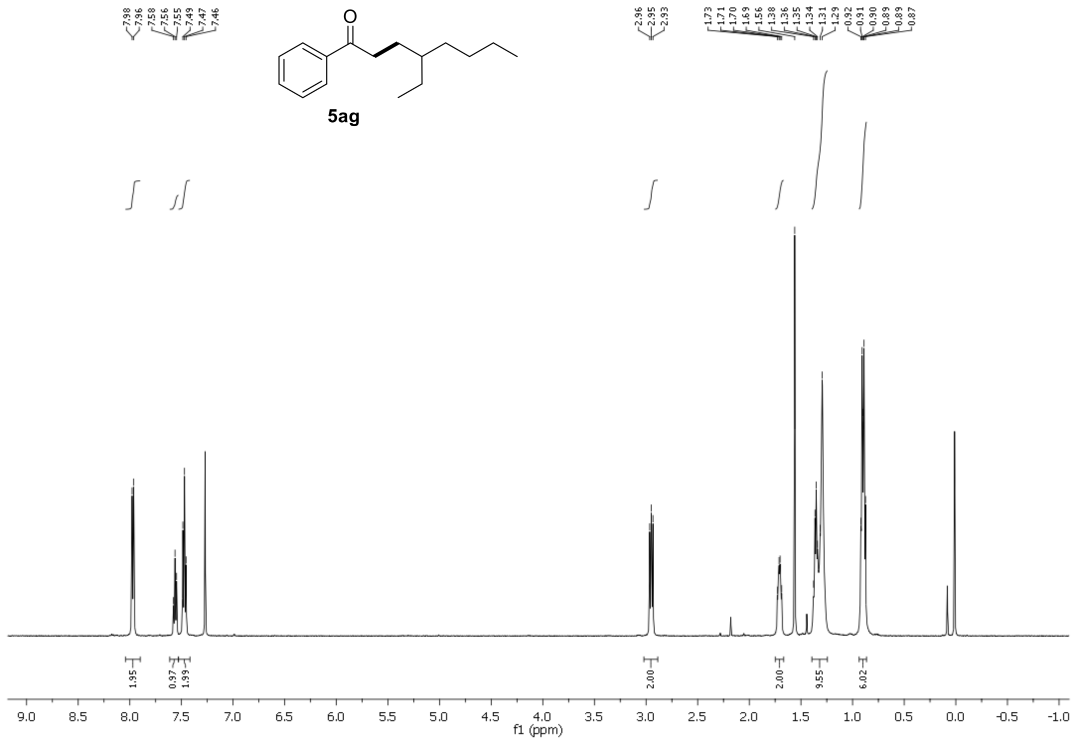

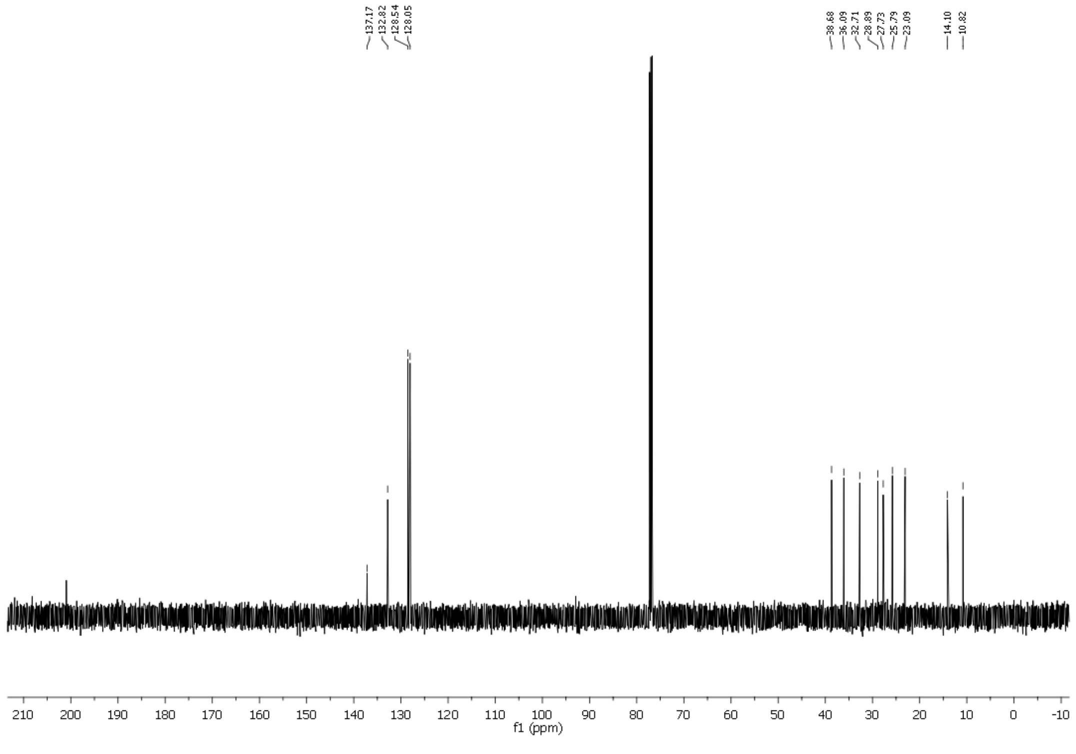

1-phenyl-tetradecan-1-one (5ah) ^{1}H NMR (500 MHz, $CDCl_3$) and ^{13}C NMR (126 MHz, $CDCl_3$)

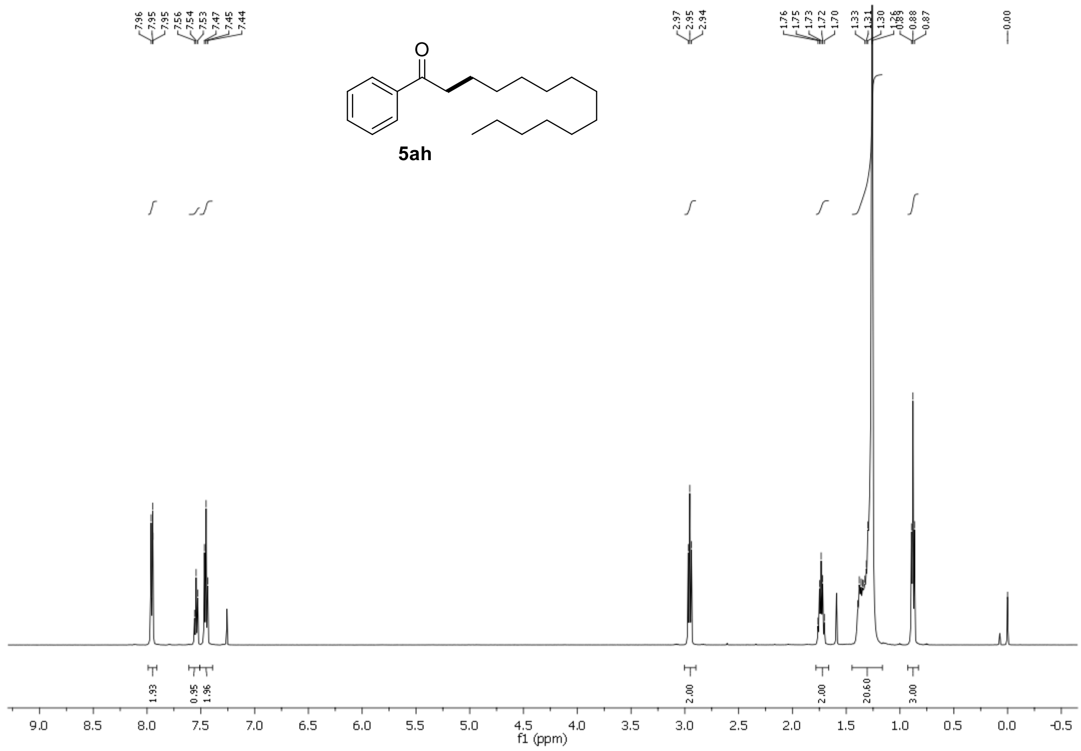

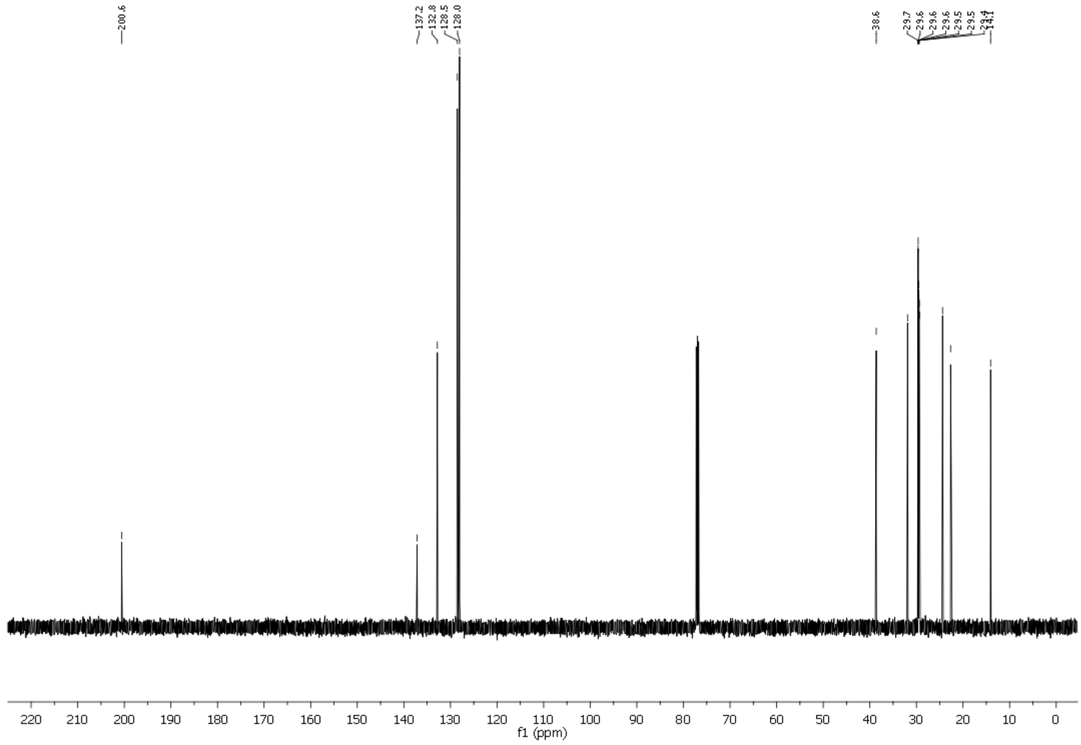

1-phenylhept-6-en-1-one (5ai) ^{1}H NMR (500 MHz, $CDCl_3$) and ^{13}C NMR (126 MHz, $CDCl_3$)

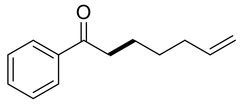

5ai

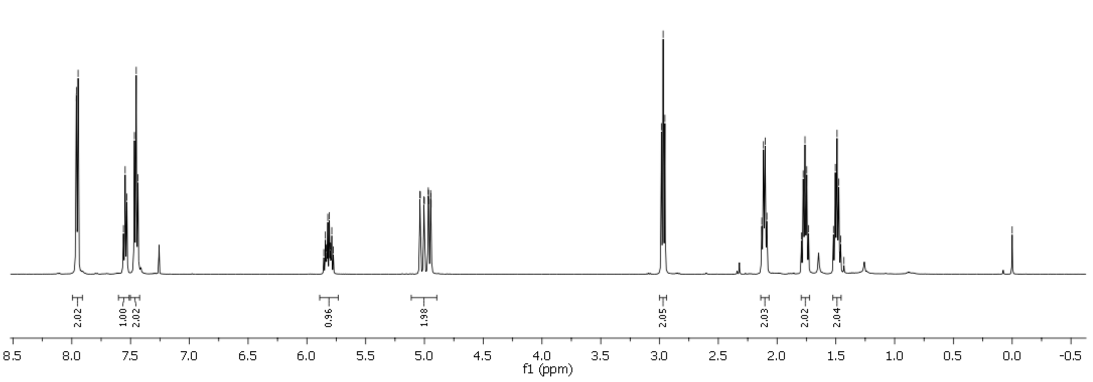

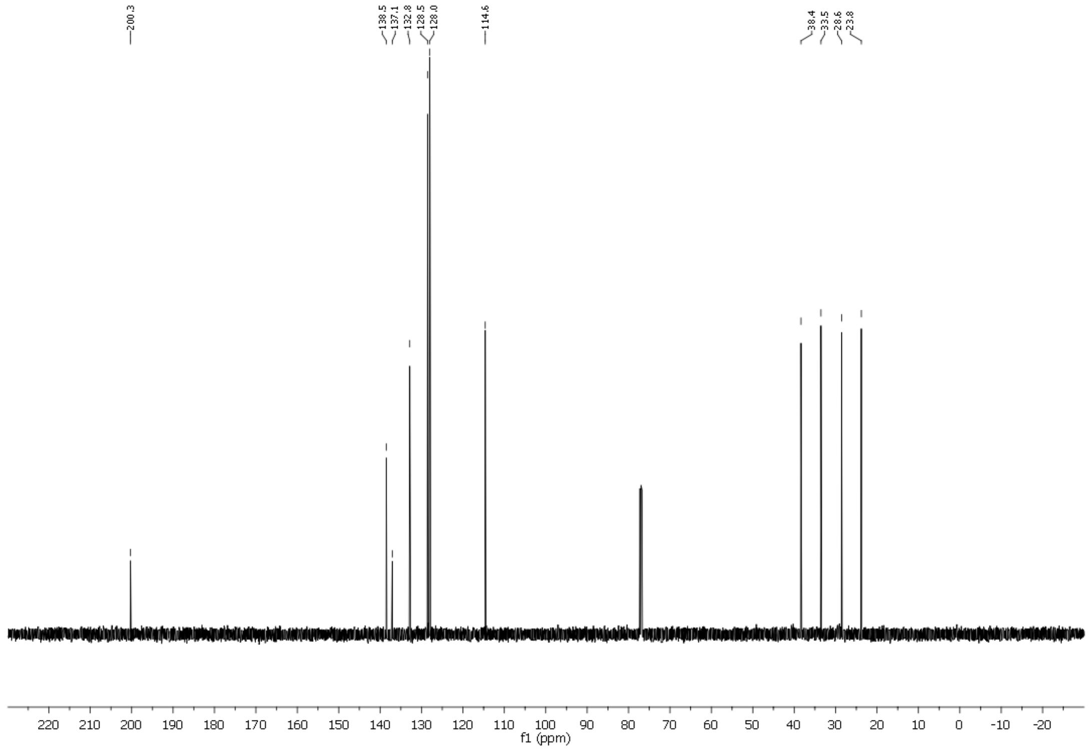

6-oxo-6-phenylhexanenitrile (5aj) ^{1}H NMR (500 MHz, $CDCl_3$) and ^{13}C NMR (126 MHz, $CDCl_3$)

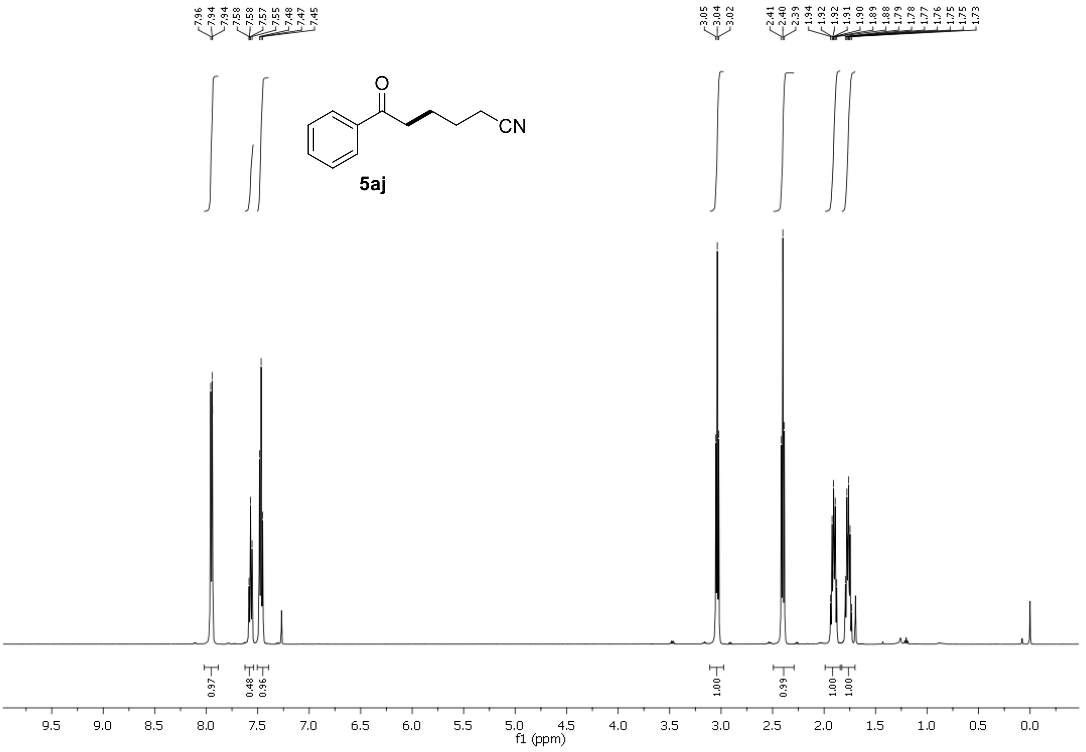

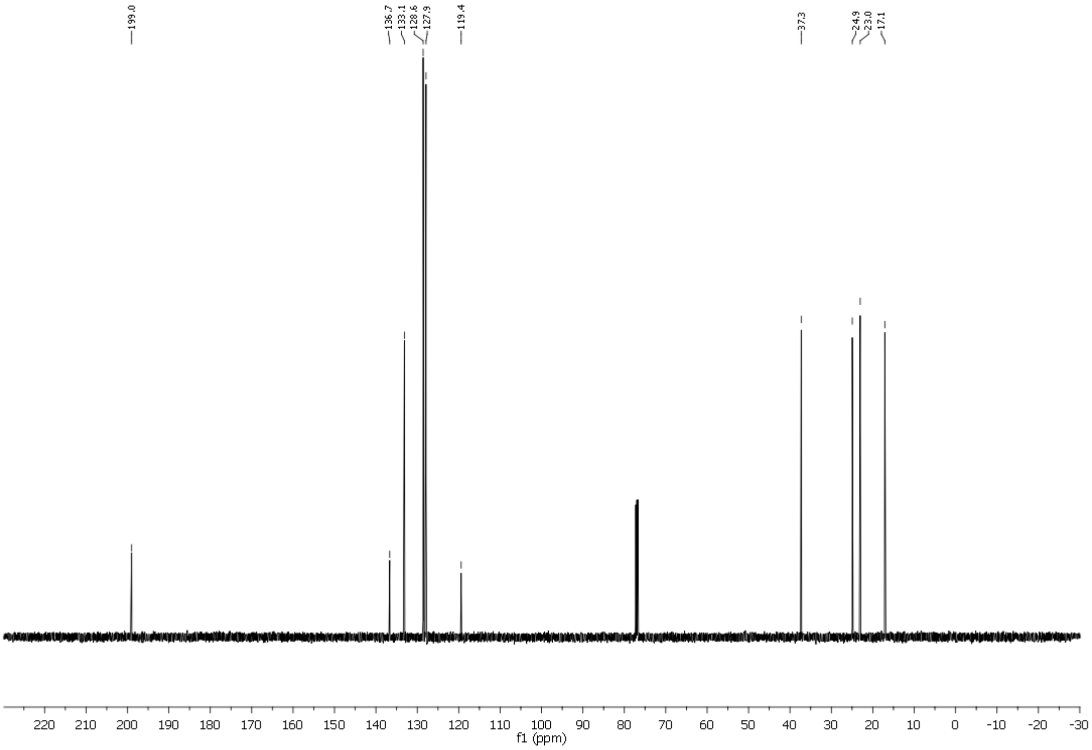

ethyl 6-oxo-6-phenylhexanoate (5ak) ^{1}H NMR (500 MHz, $CDCl_3$) and ^{13}C NMR (126 MHz, $CDCl_3$)

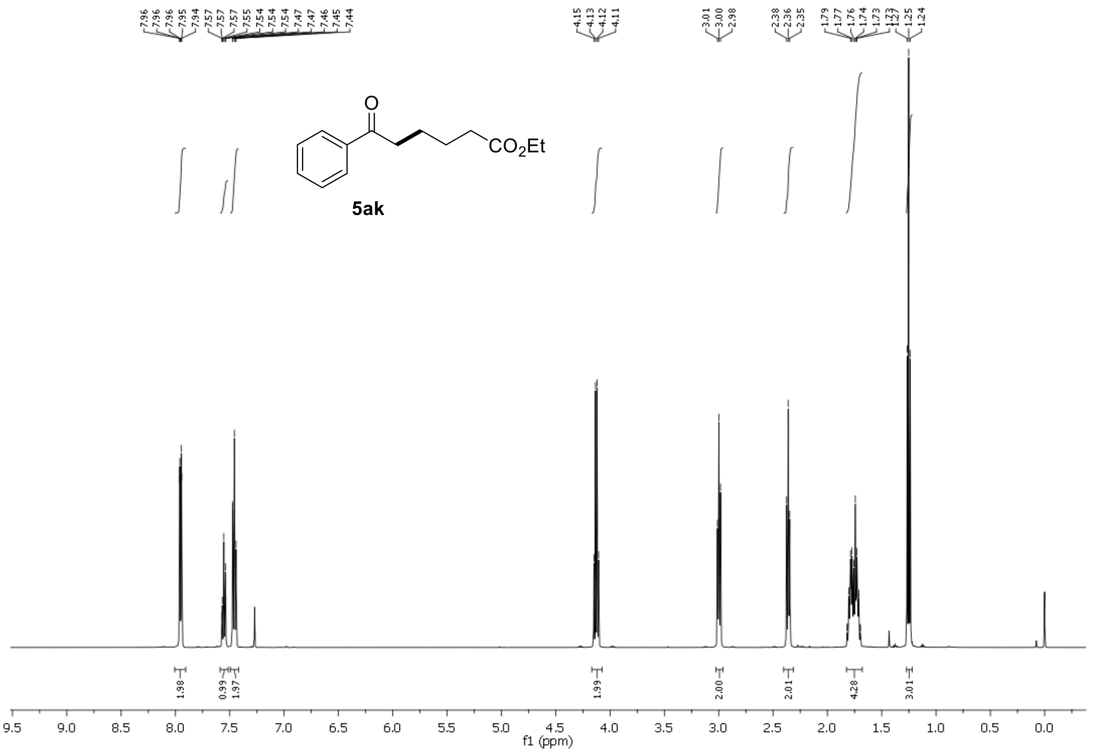

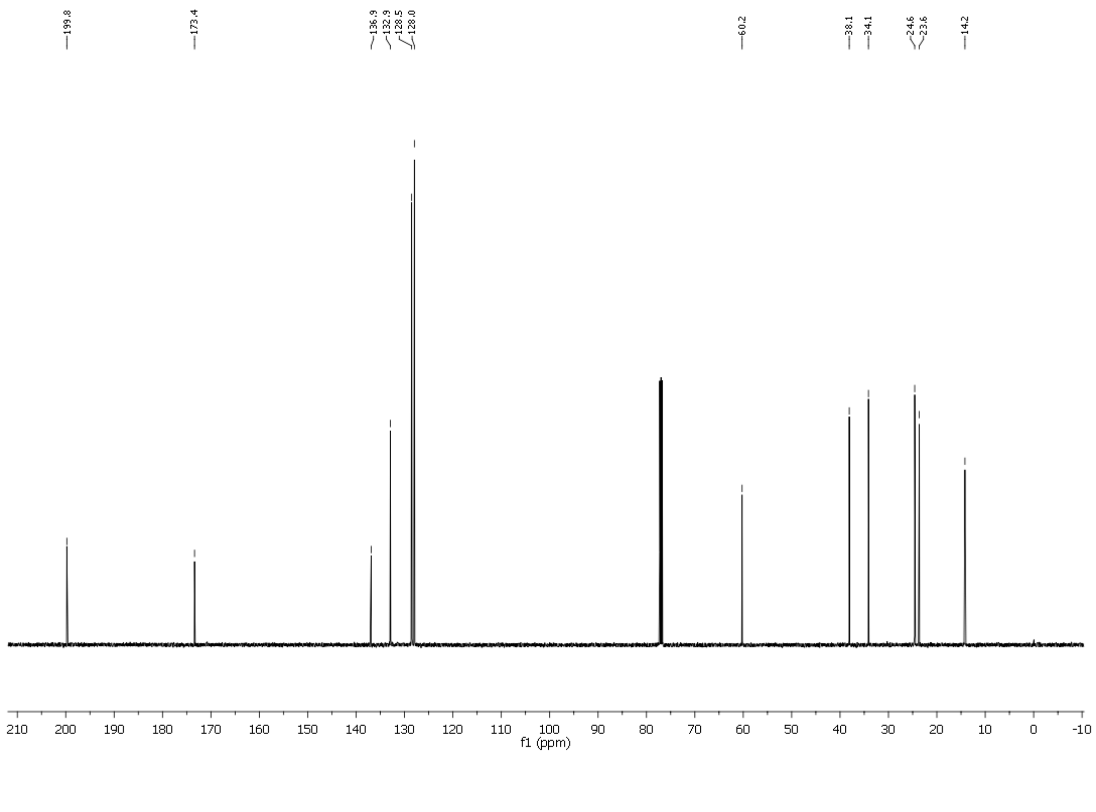

2-(5-oxo-5-phenylpentyl)isoindole-1,3-dione (5al) ^{1}H NMR (500 MHz, $CDCl_3$) and ^{13}C NMR (126 MHz, $CDCl_3$)

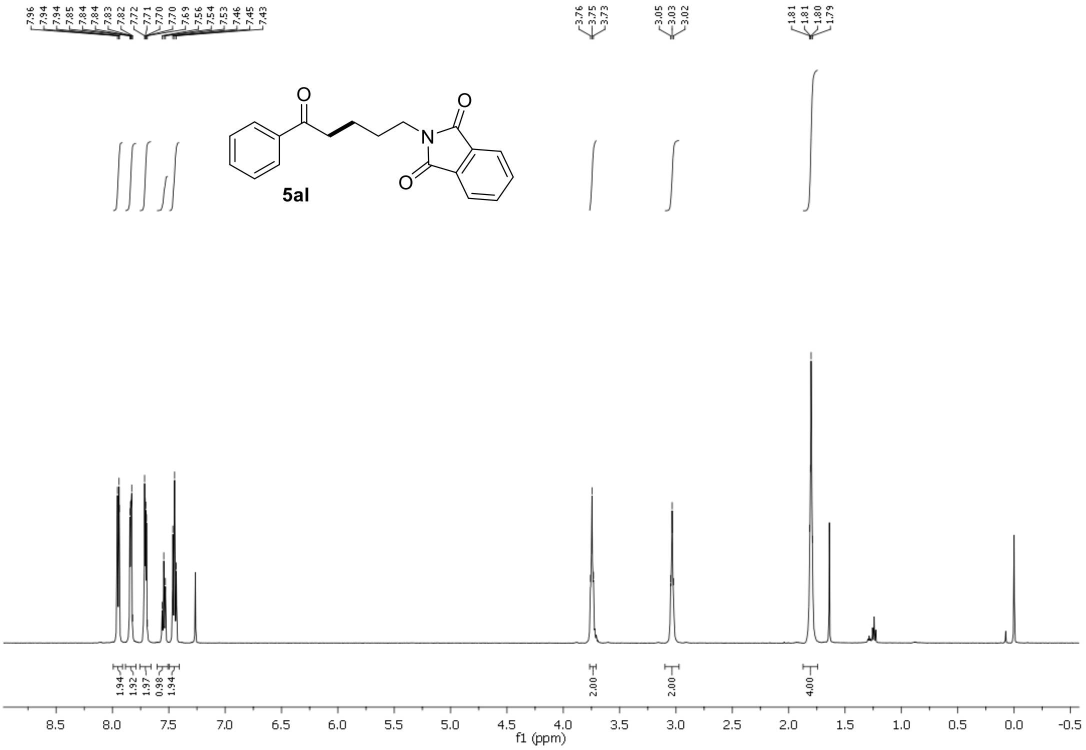

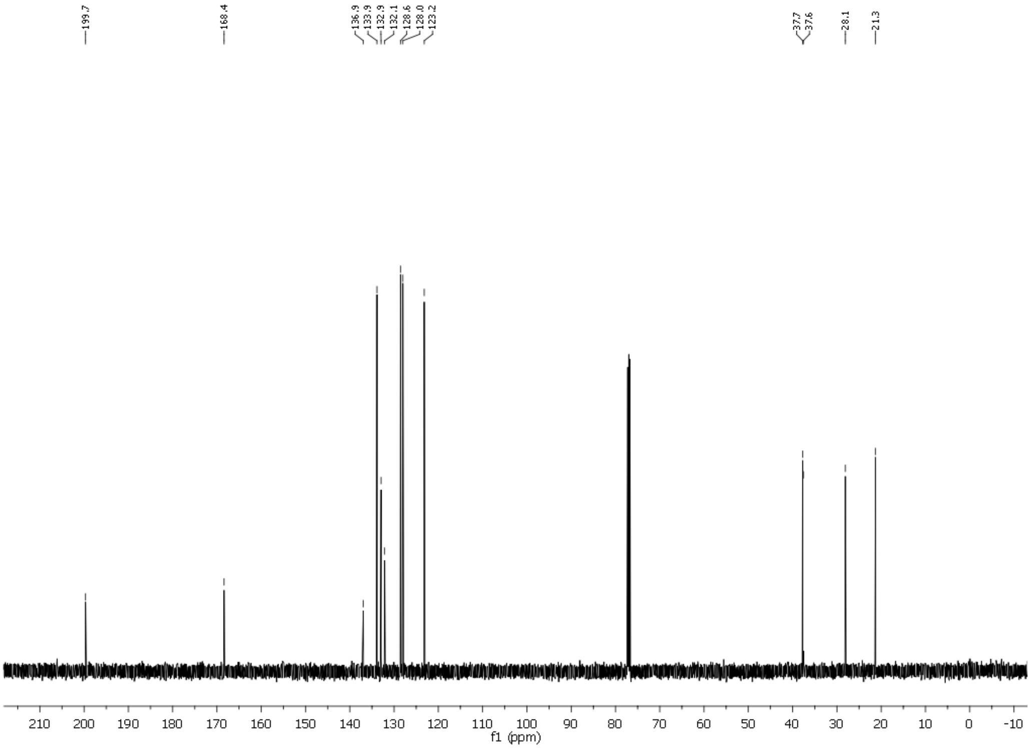

1-phenyl-3-(1-tosylpyrrolidin-3-yl)propan-1-one (5am) ^{1}H NMR (500 MHz, $CDCl_3$) and ^{13}C NMR (126 MHz, $CDCl_3$)

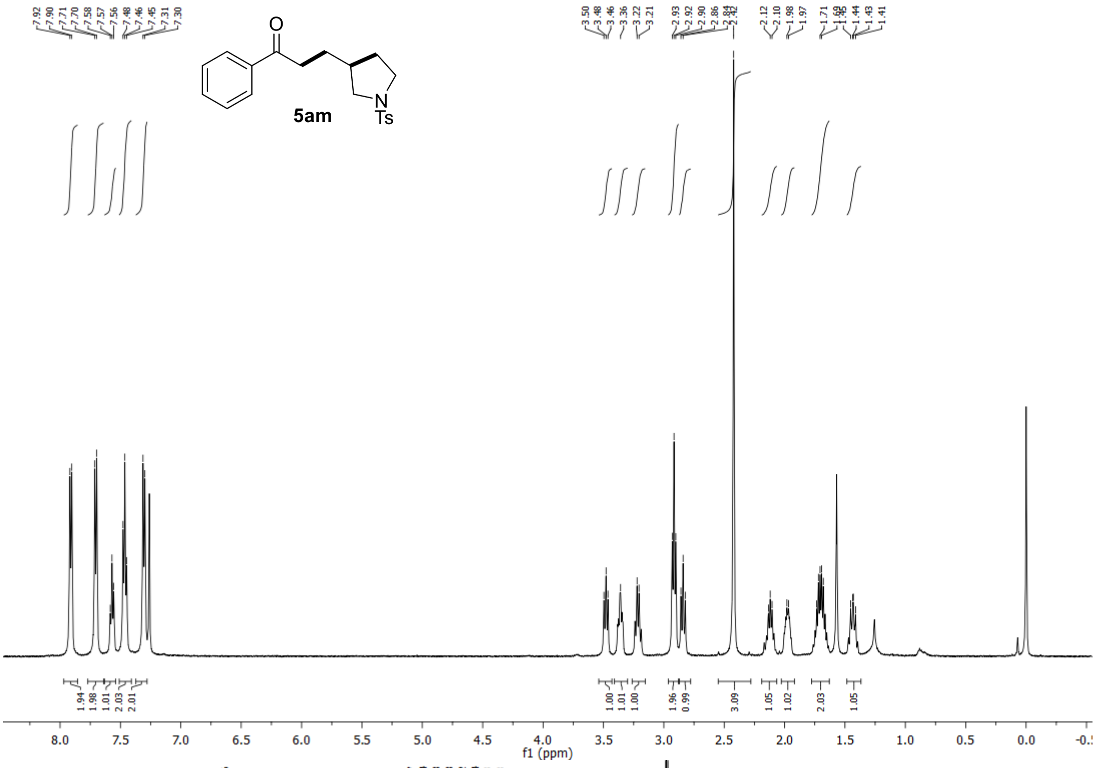

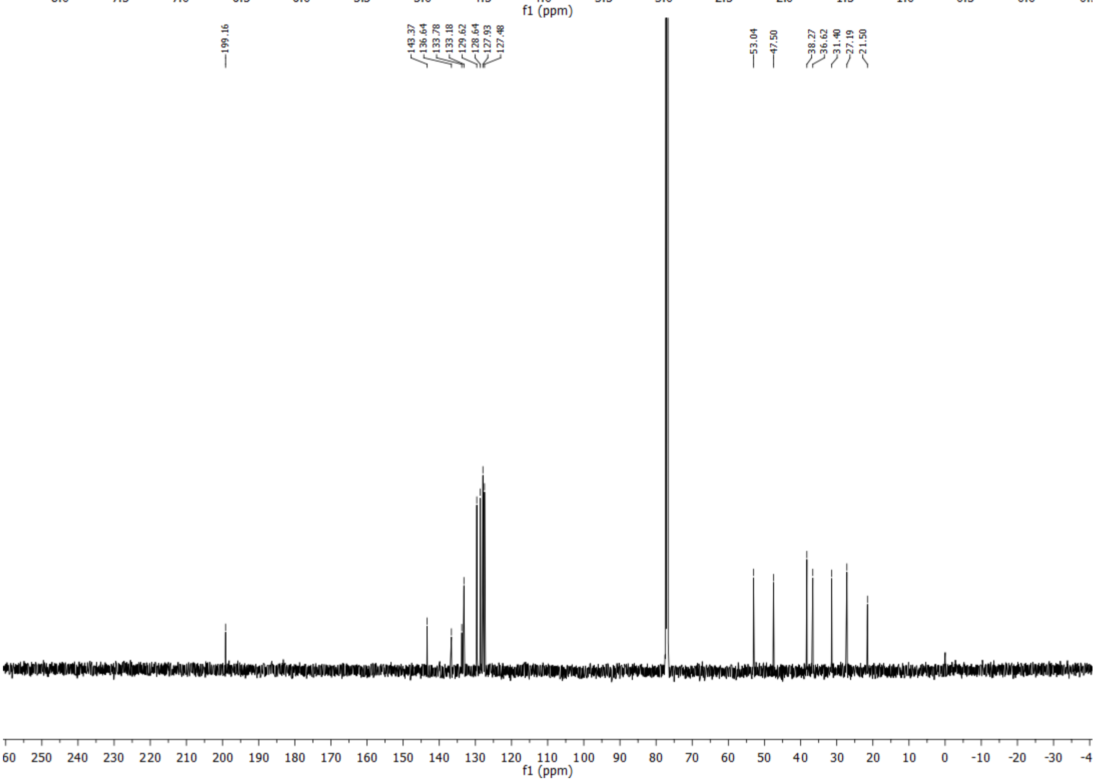

4-(benzyloxy)-1-phenylbutan-1-one (5an) ^{1}H NMR (500 MHz, $CDCl_3$) and ^{13}C NMR (126 MHz, $CDCl_3$)

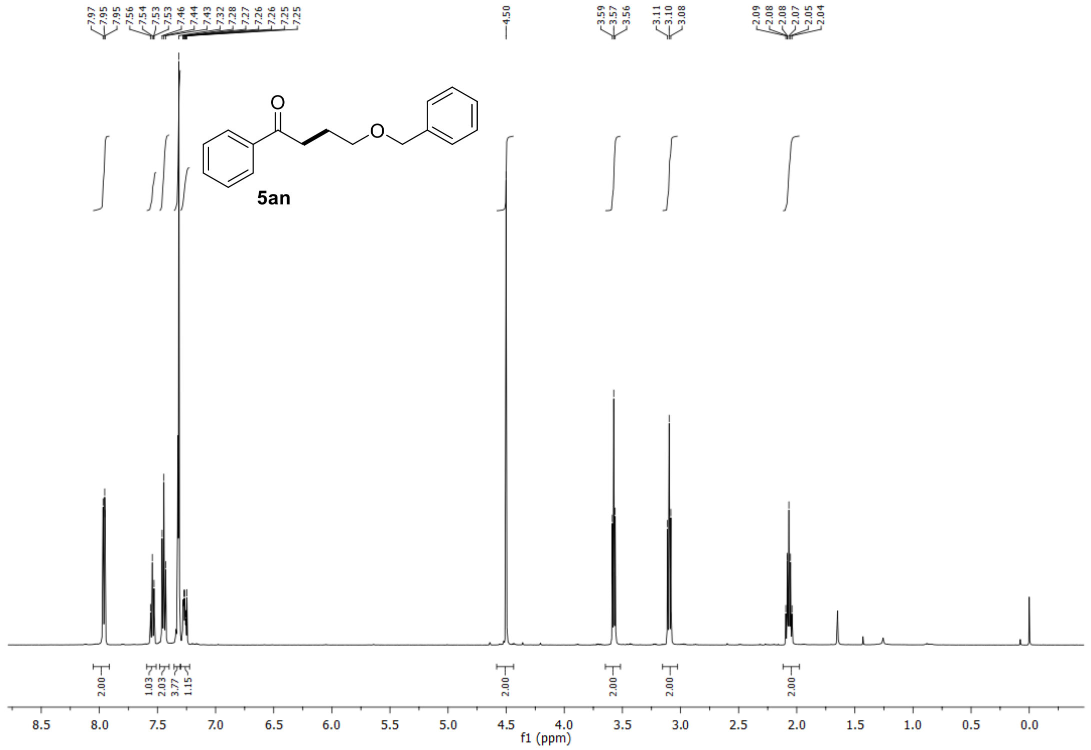

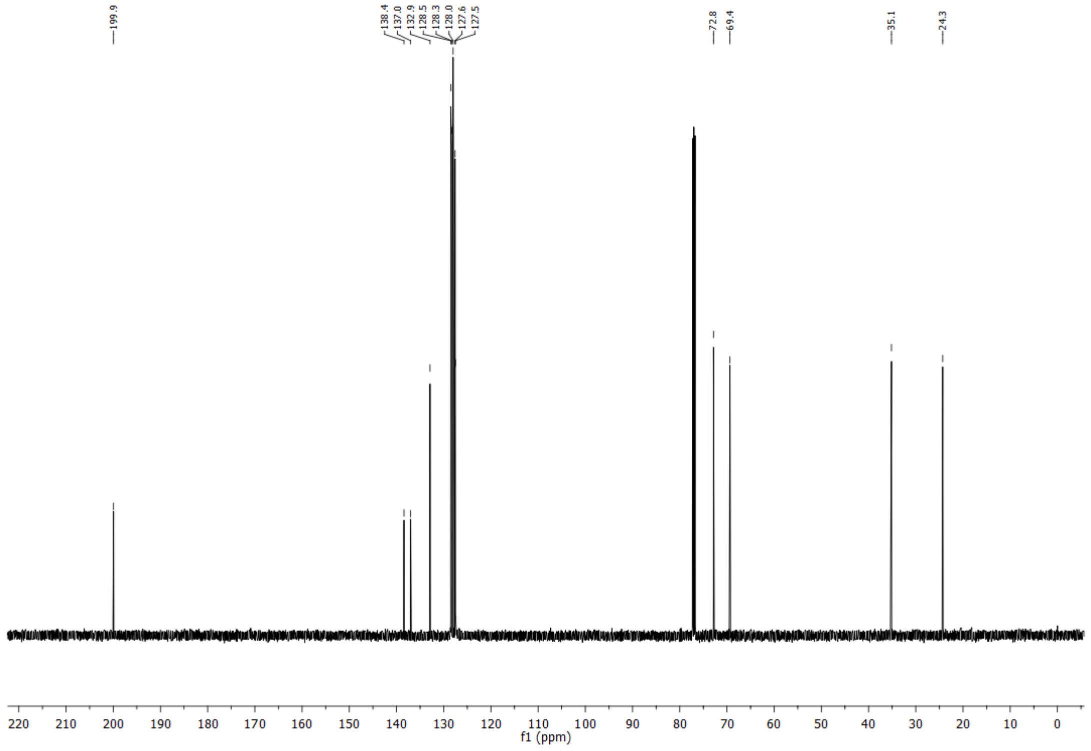

10-hydroxydecanophenone (5ao) ^{1}H NMR (500 MHz, $CDCl_3$) and ^{13}C NMR (126 MHz, $CDCl_3$)

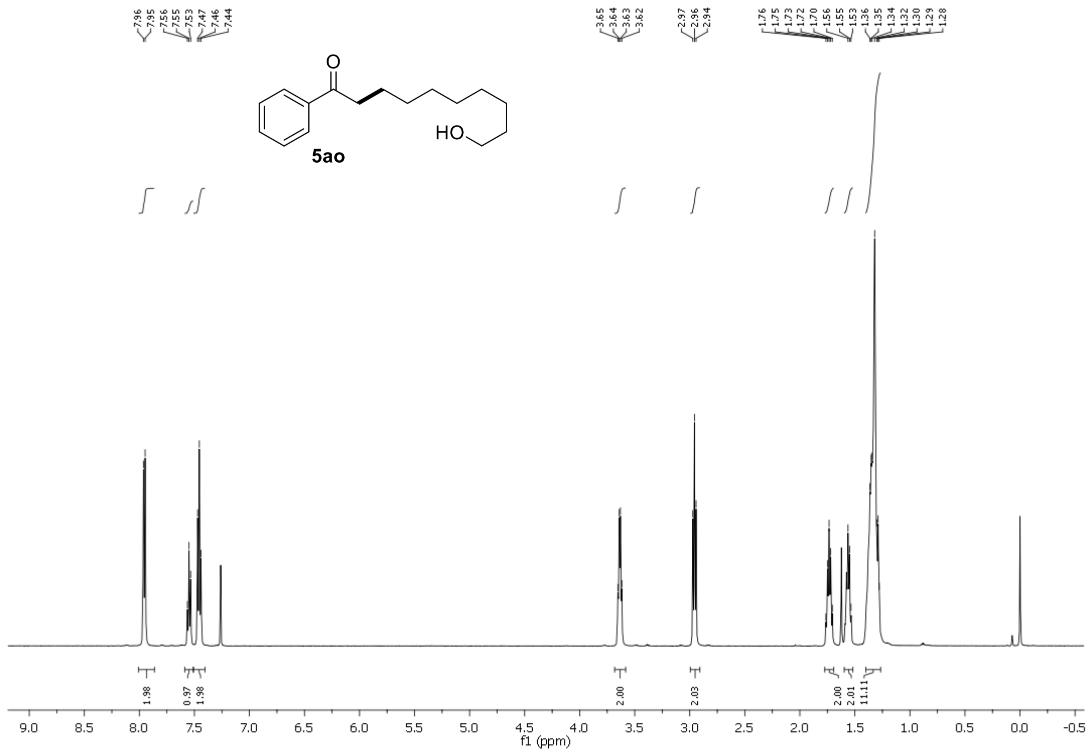

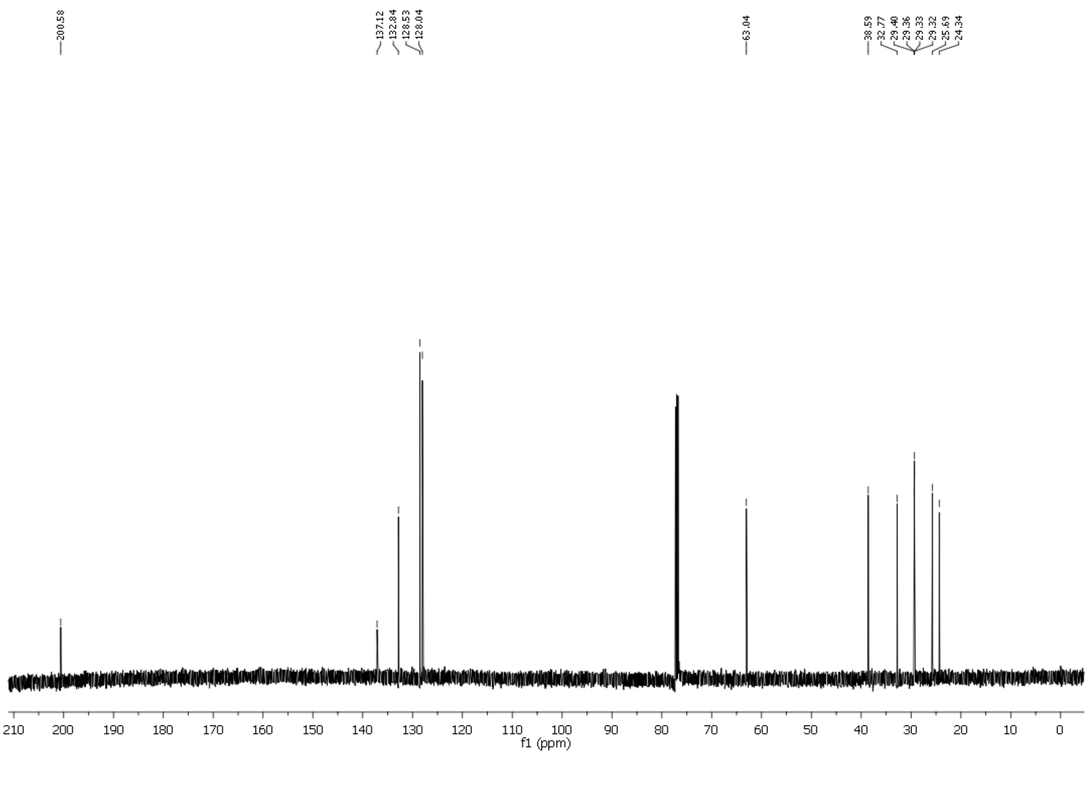

4-(2-(2-methoxyethoxy)ethoxy)-1-phenylbutan-1-one (5ap) ^{1}H NMR (500 MHz, $CDCl_3$) and ^{13}C NMR (126 MHz, $CDCl_3$)

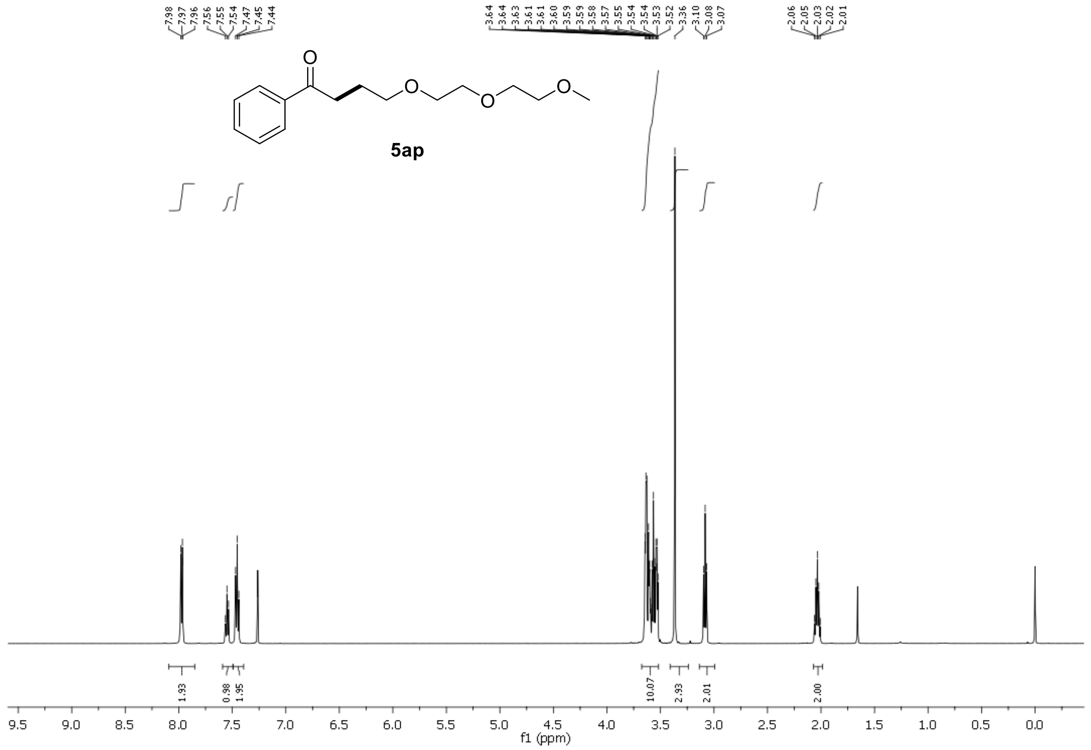

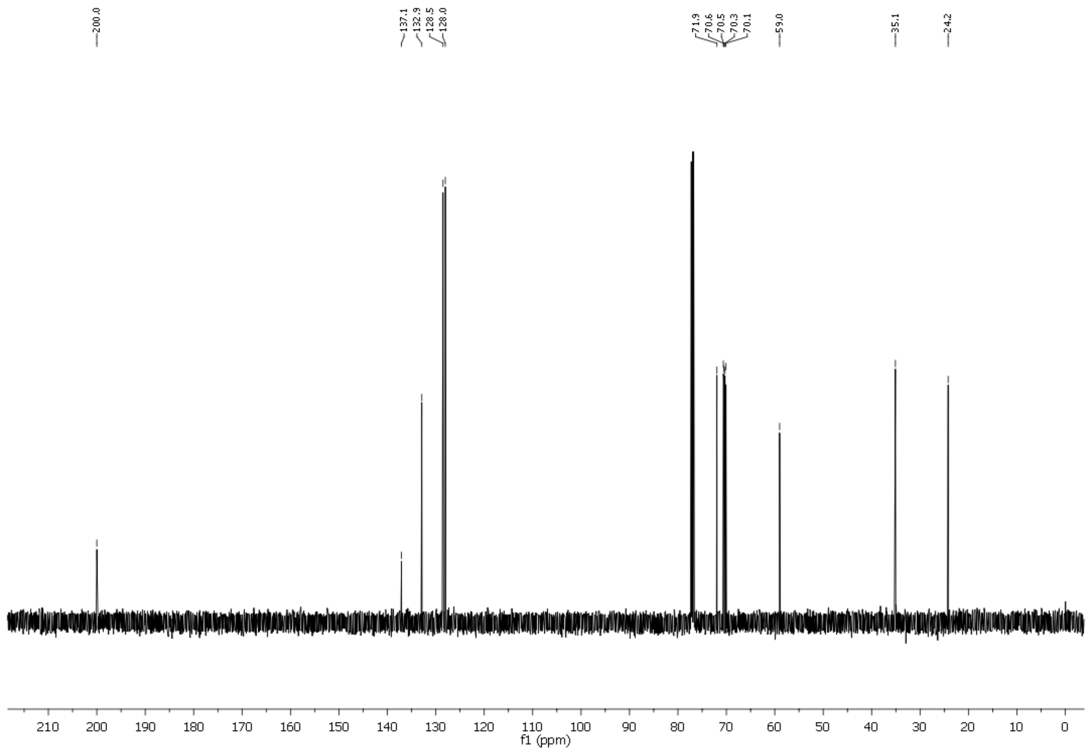

1-(4-(tert-butyl)phenyl)-5-phenylpentan-1-one (5oa) ^{1}H NMR (500 MHz, $CDCl_3$) and ^{13}C NMR (126 MHz, $CDCl_3$)

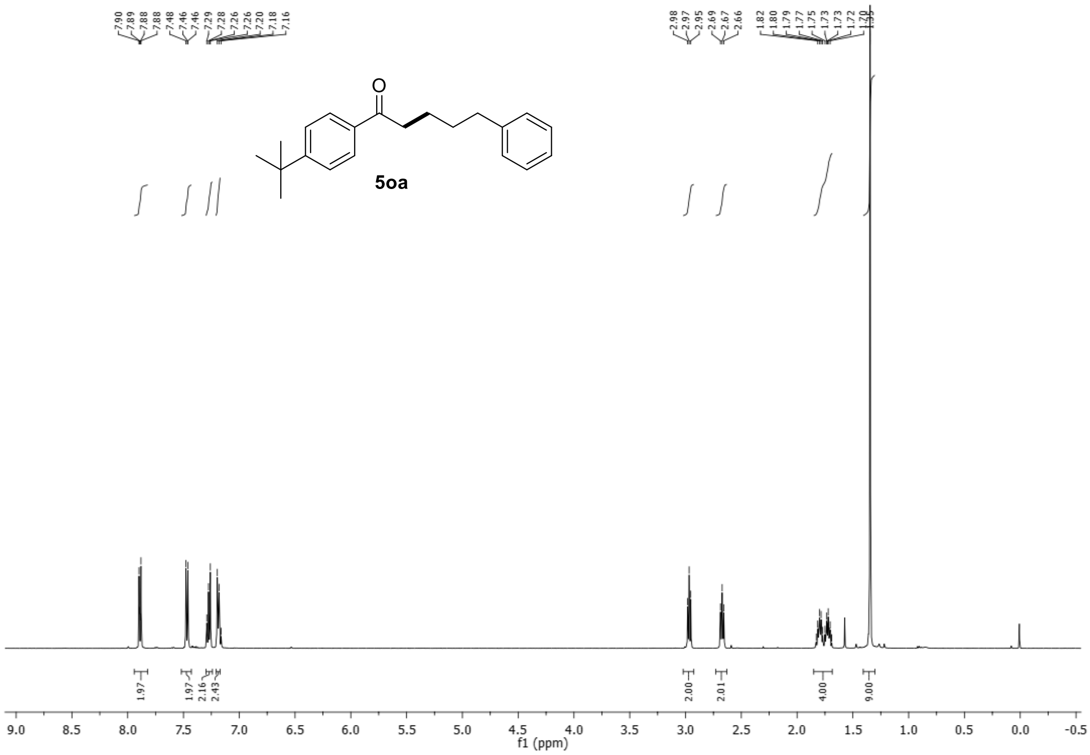

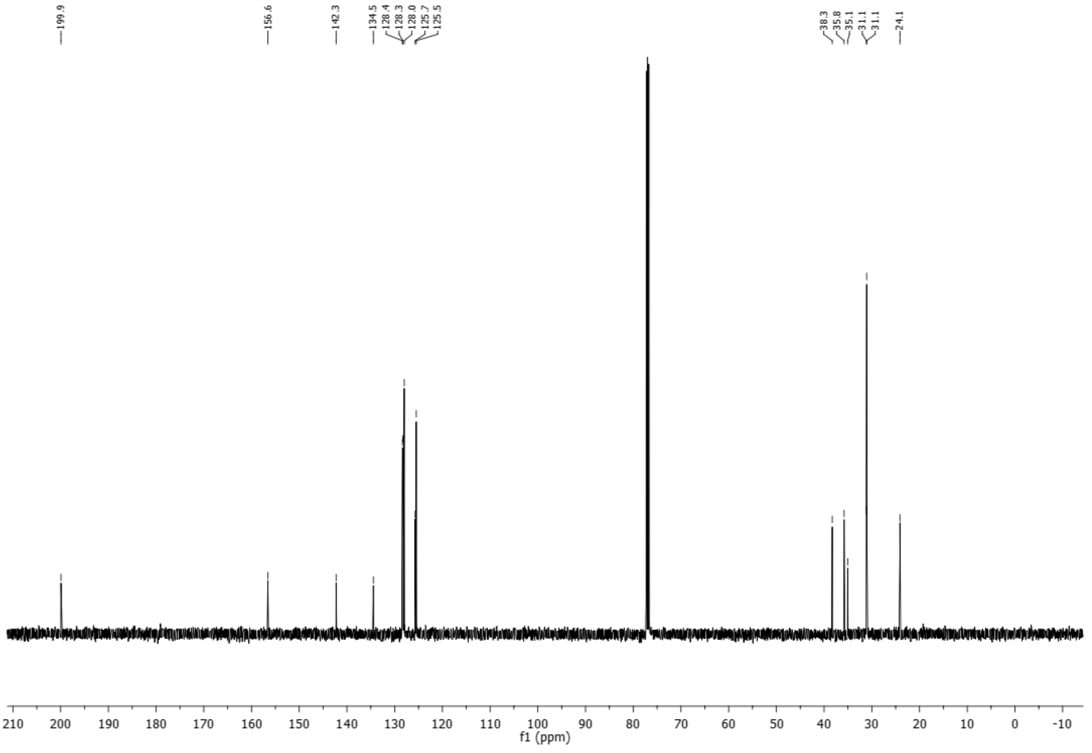

1-(naphthalen-2-yl)-5-phenylpentan-1-one (5pa) ^{1}H NMR (400 MHz, CDCl$_3$) and ^{13}C NMR (100 MHz, CDCl$_3$)

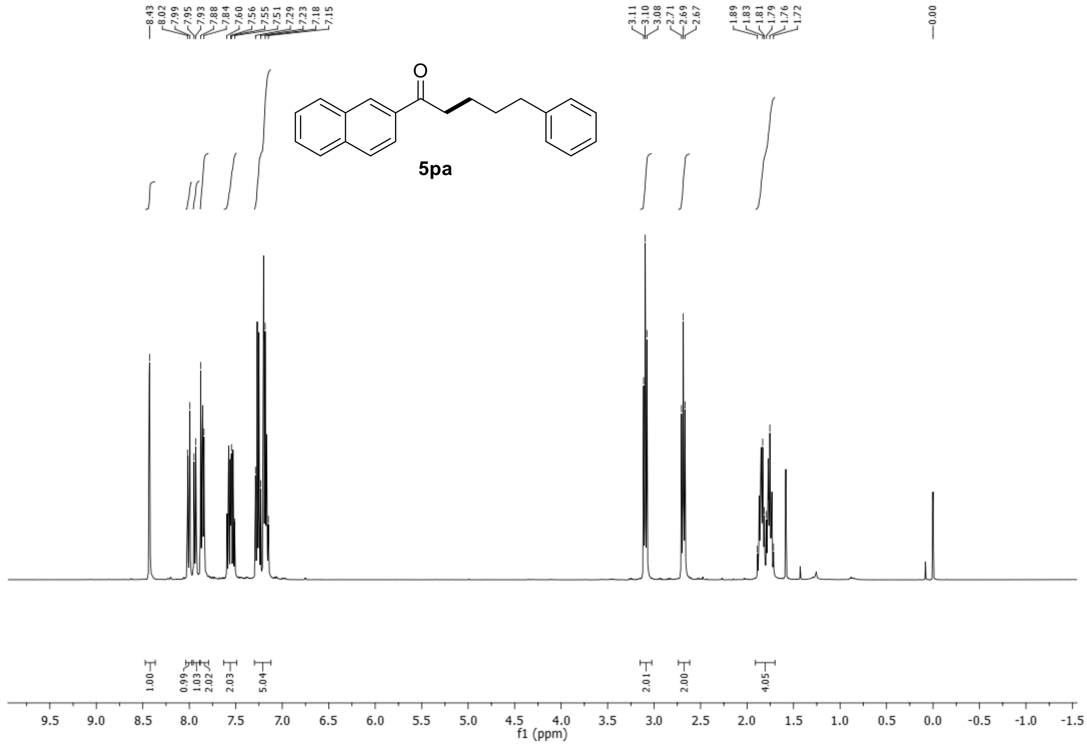

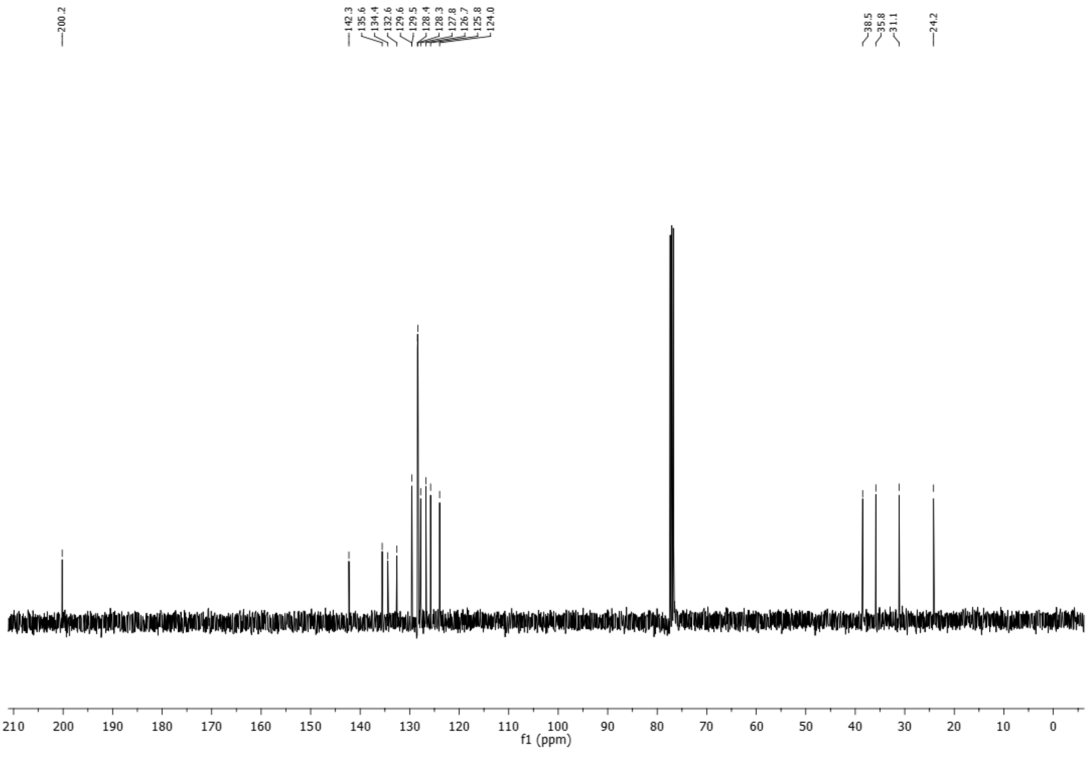

1-([1,1'-biphenyl]-4-yl)-5-phenylpentan-1-one (5qa) ^{1}H NMR (500 MHz, $CDCl_3$) and ^{13}C NMR (126 MHz, $CDCl_3$)

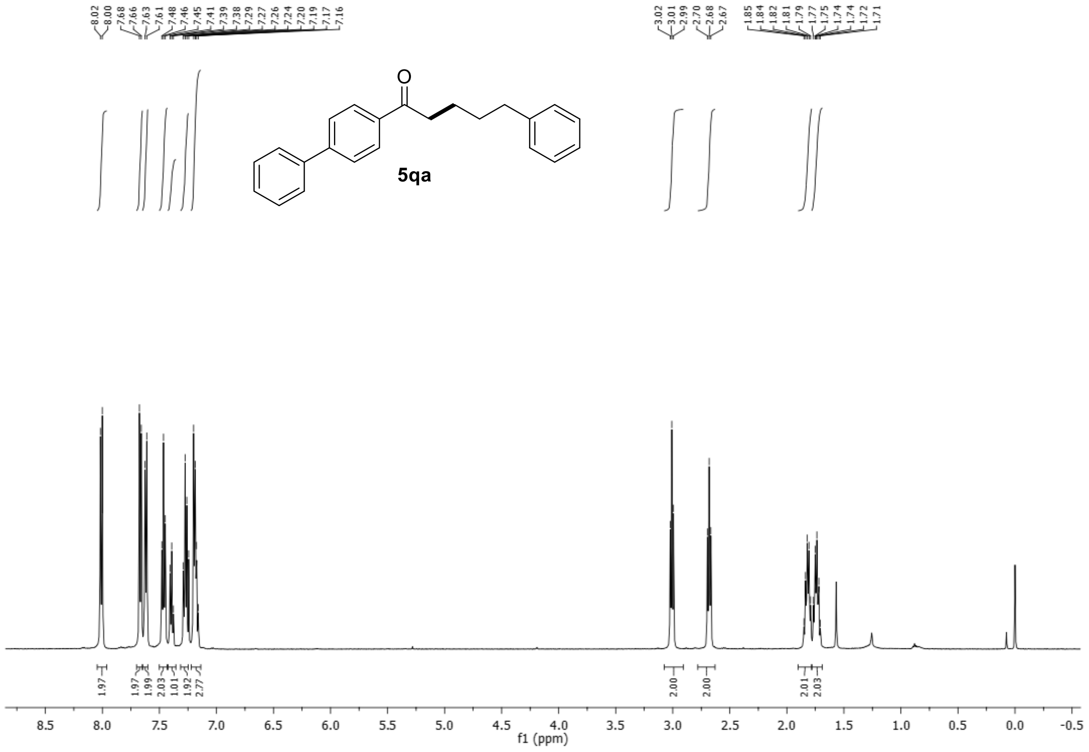

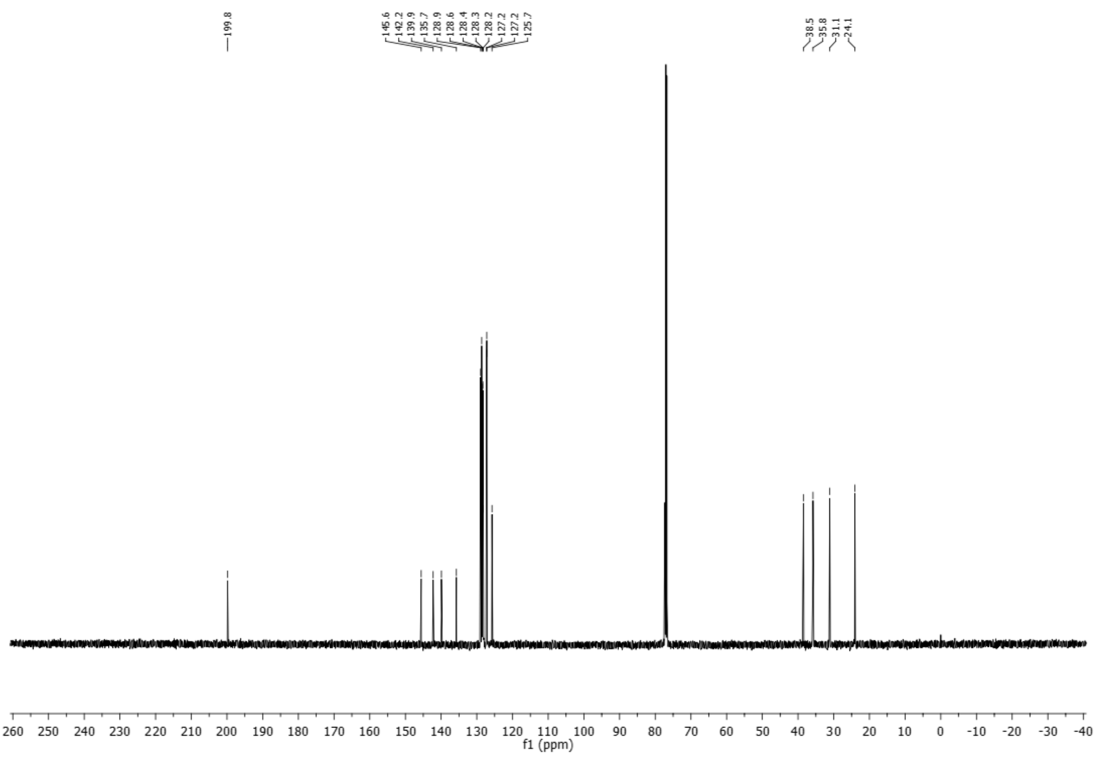

1-(4-chlorophenyl)-5-phenylpentan-1-one (5ra) ^{1}H NMR (400 MHz, $CDCl_3$) and ^{13}C NMR (126 MHz, $CDCl_3$)

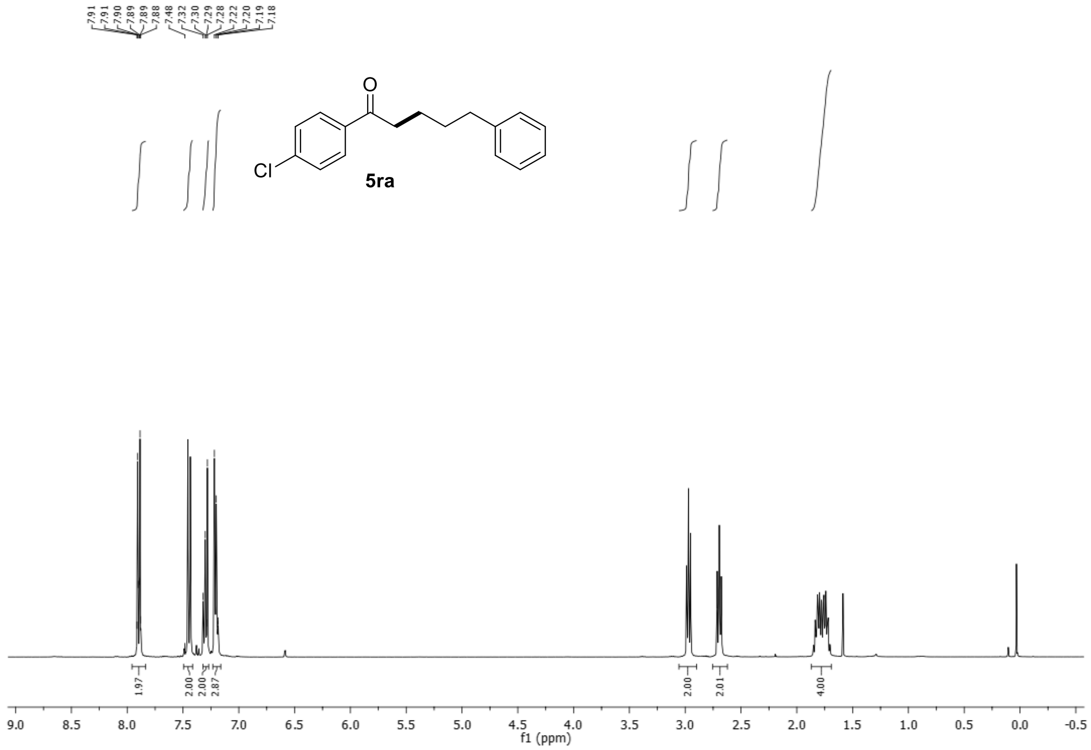

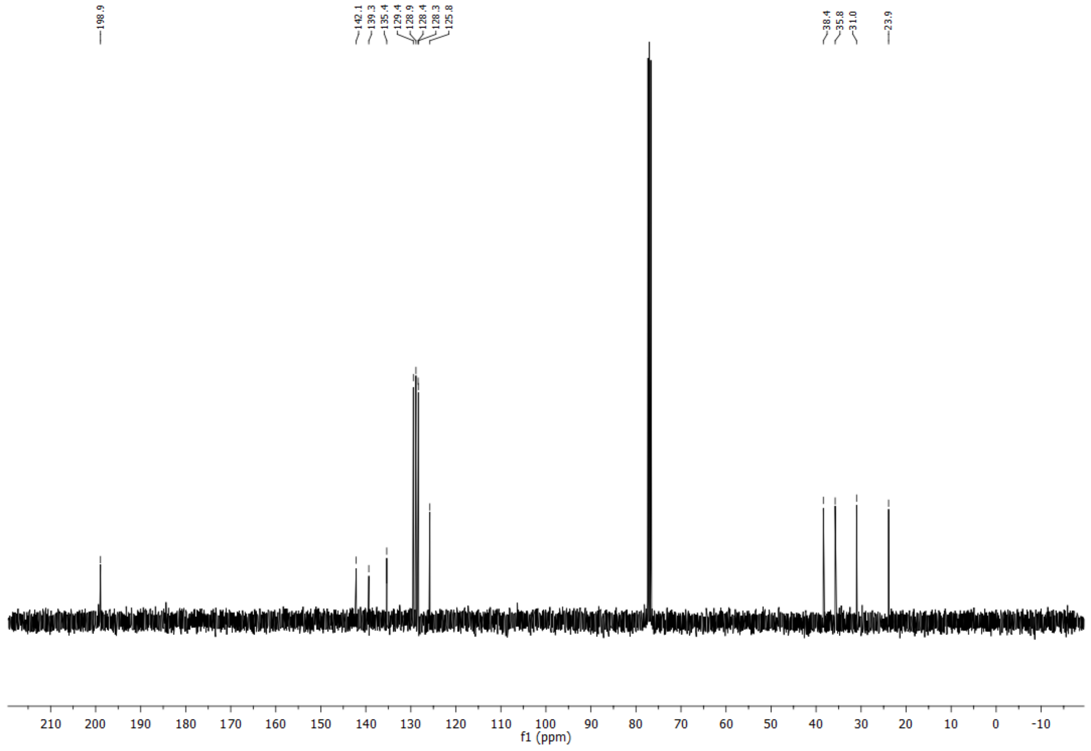

1-(4-methoxyphenyl)-5-phenylpentan-1-one (5sa) ^{1}H NMR (500 MHz, $CDCl_3$) and ^{13}C NMR (126 MHz, $CDCl_3$)

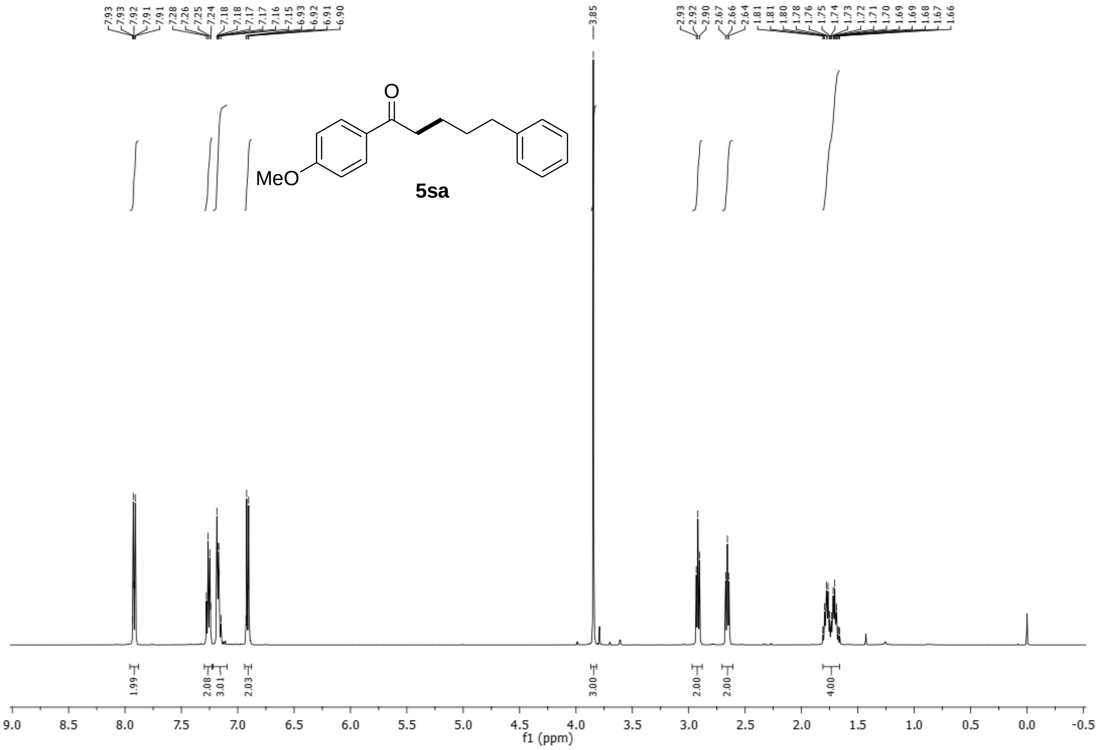

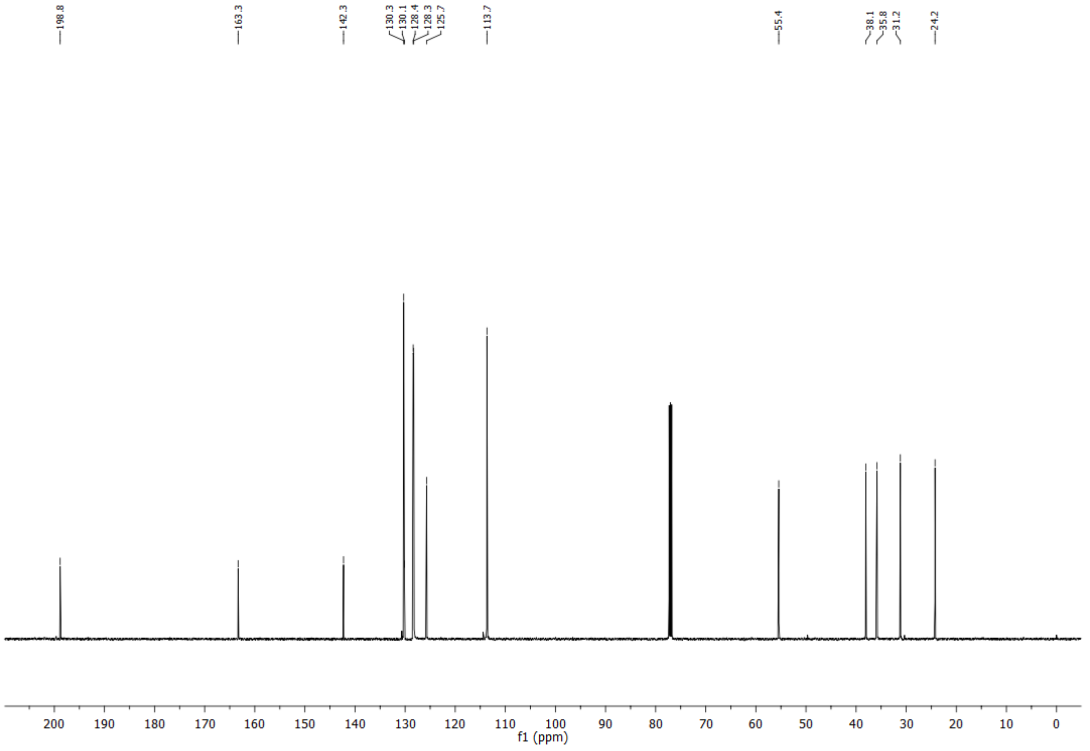

1-(3-methoxyphenyl)-5-phenylpentan-1-one (5sb) ^{1}H NMR (500 MHz, $CDCl_3$) and ^{13}C NMR (126 MHz, $CDCl_3$)

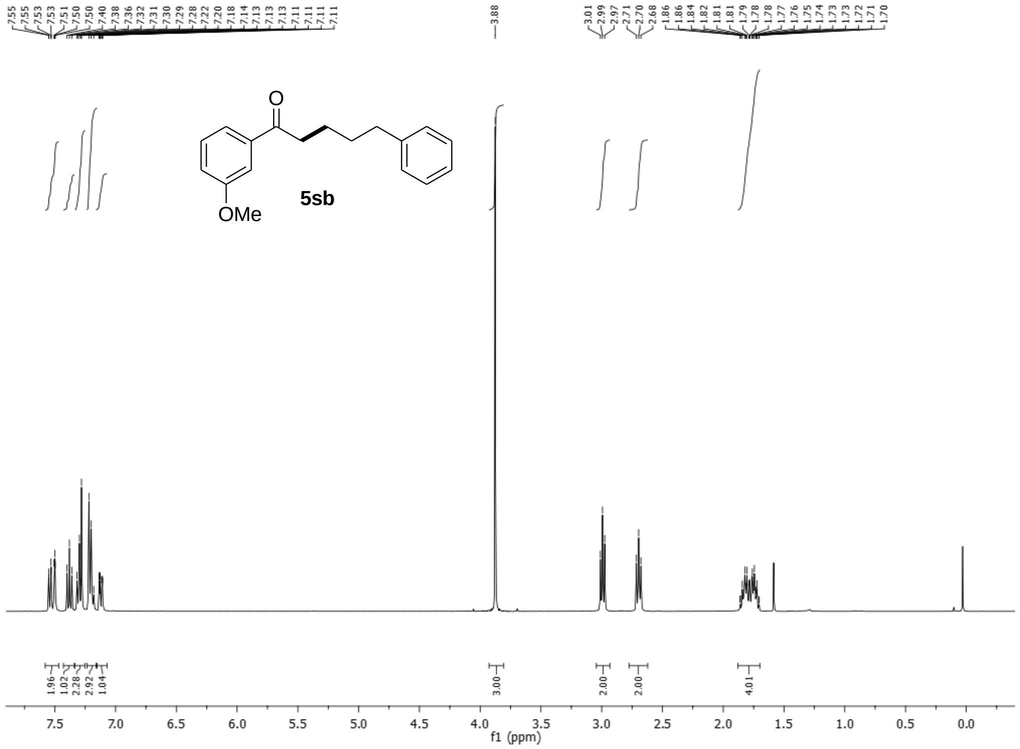

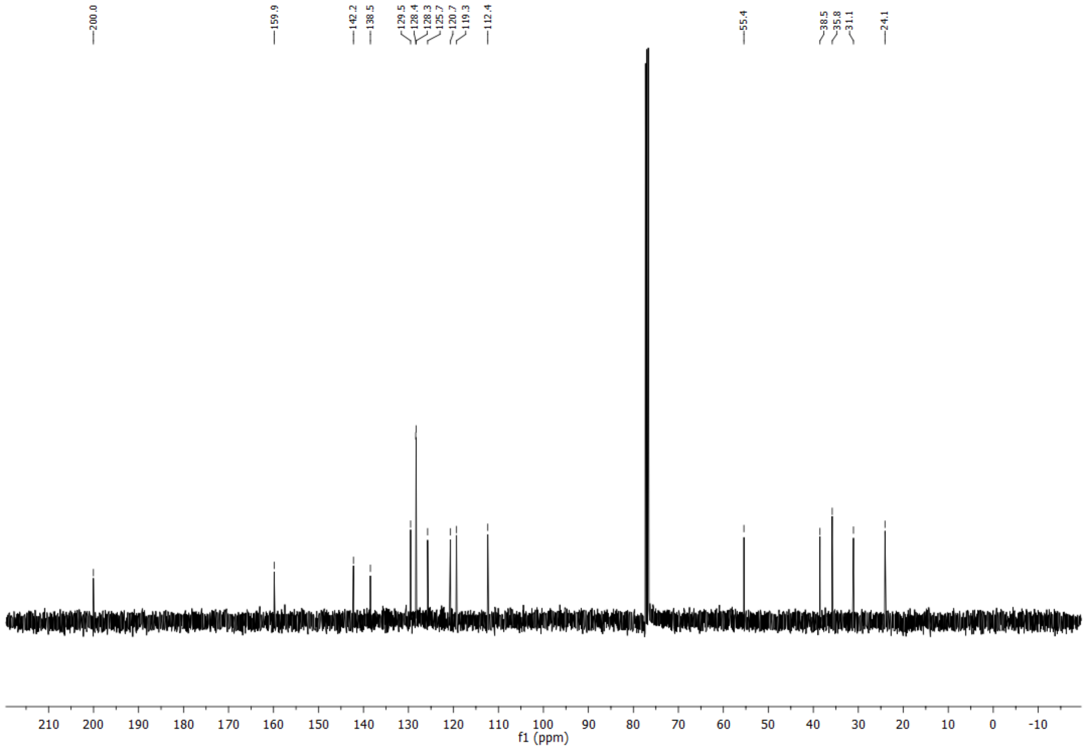

1-cyclohexenyl-5-phenylpentan-1-one (5ta) ^{1}H NMR (400 MHz, $CDCl_3$) and ^{13}C NMR (100 MHz, $CDCl_3$)

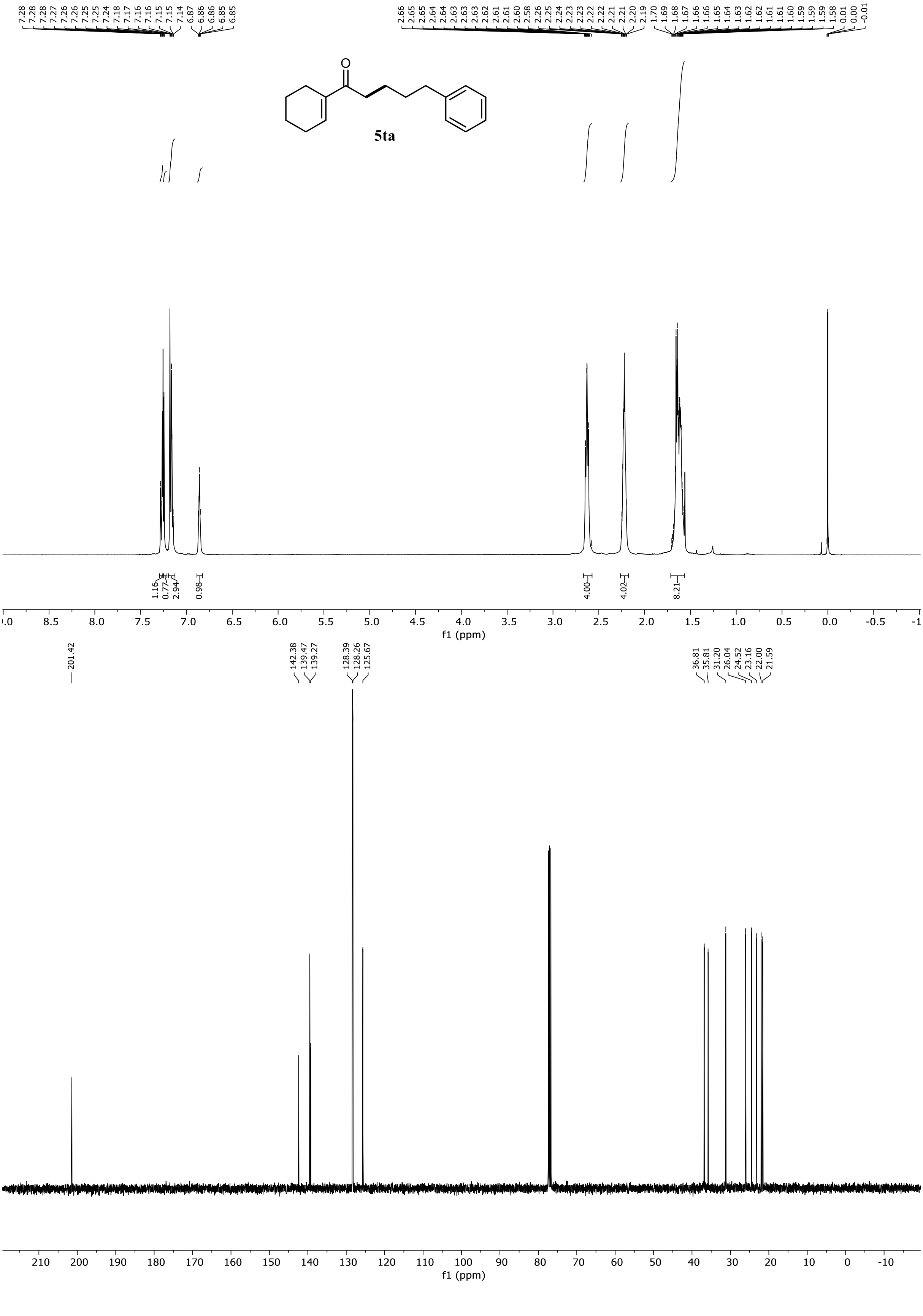

1-cyclohexenyl-5-(4-(tert-butyl)phenyl)pentan-1-one (5tb) ^{1}H NMR (400 MHz, $CDCl_3$) and ^{13}C NMR (100 MHz, $CDCl_3$)

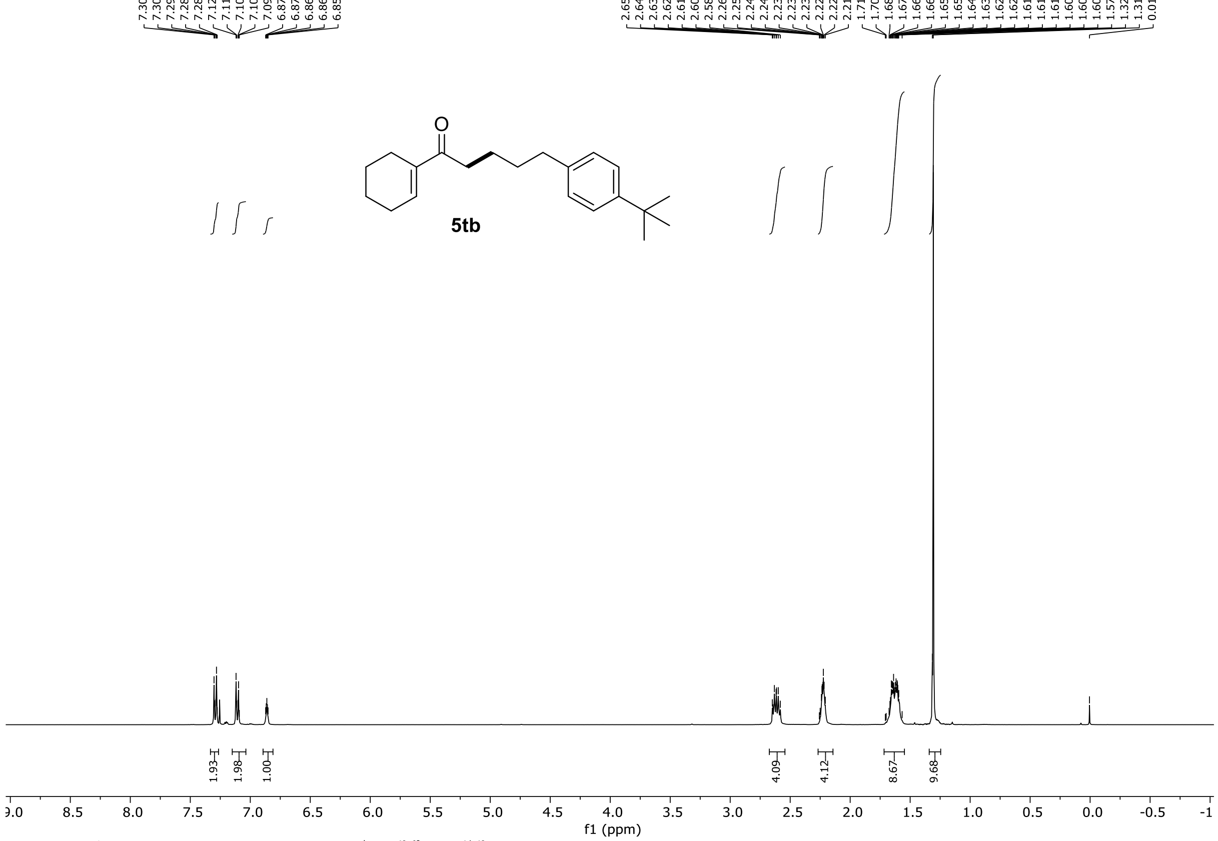

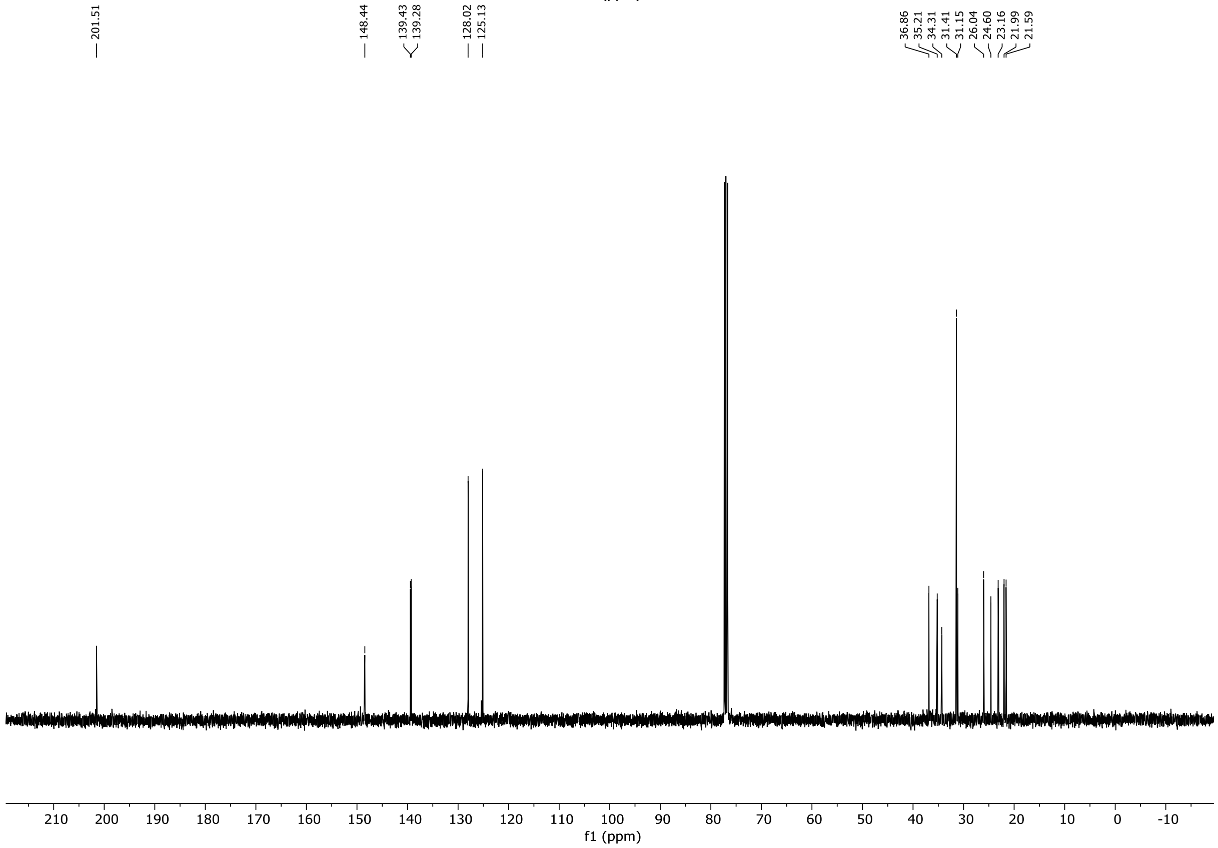

methyl 5-oxo-9-phenyl-nonanoate (5ua) ^{1}H NMR (400 MHz, $CDCl_3$) and ^{13}C NMR (100 MHz, $CDCl_3$)

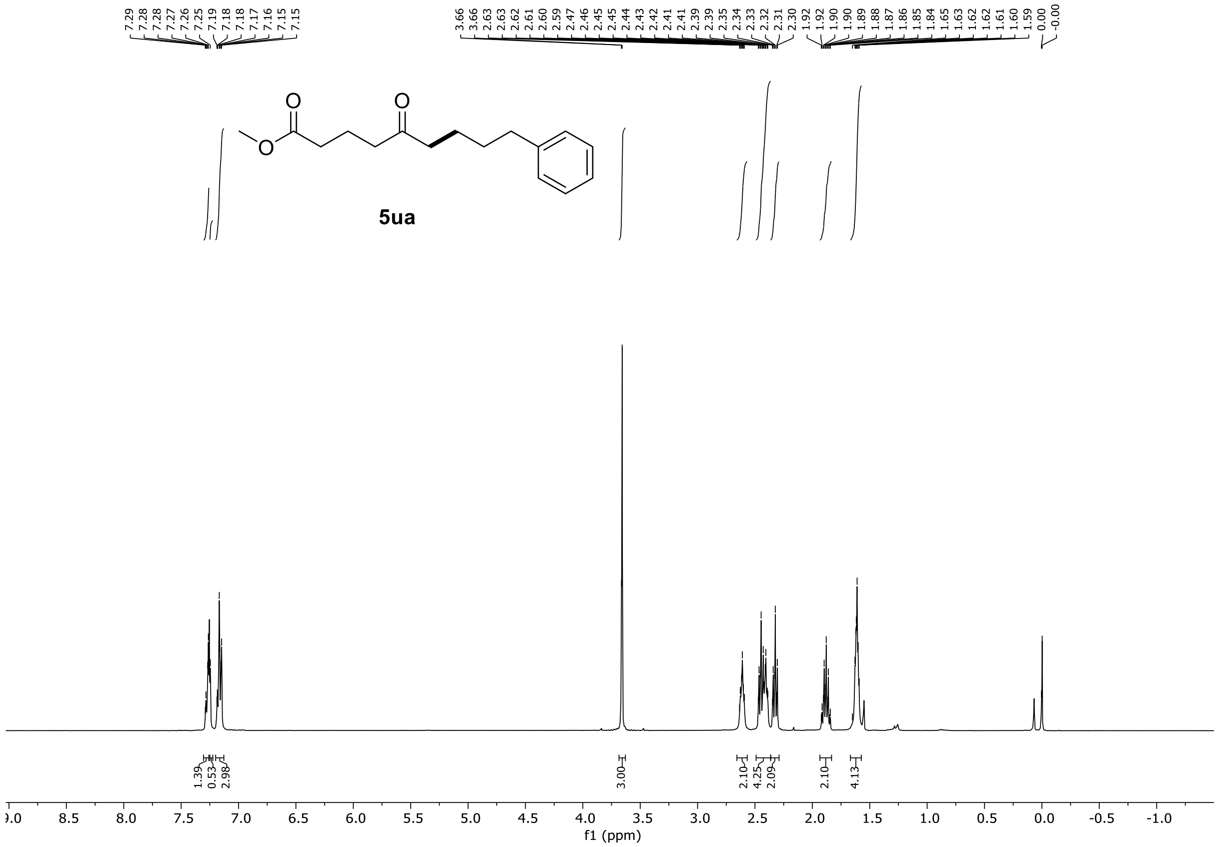

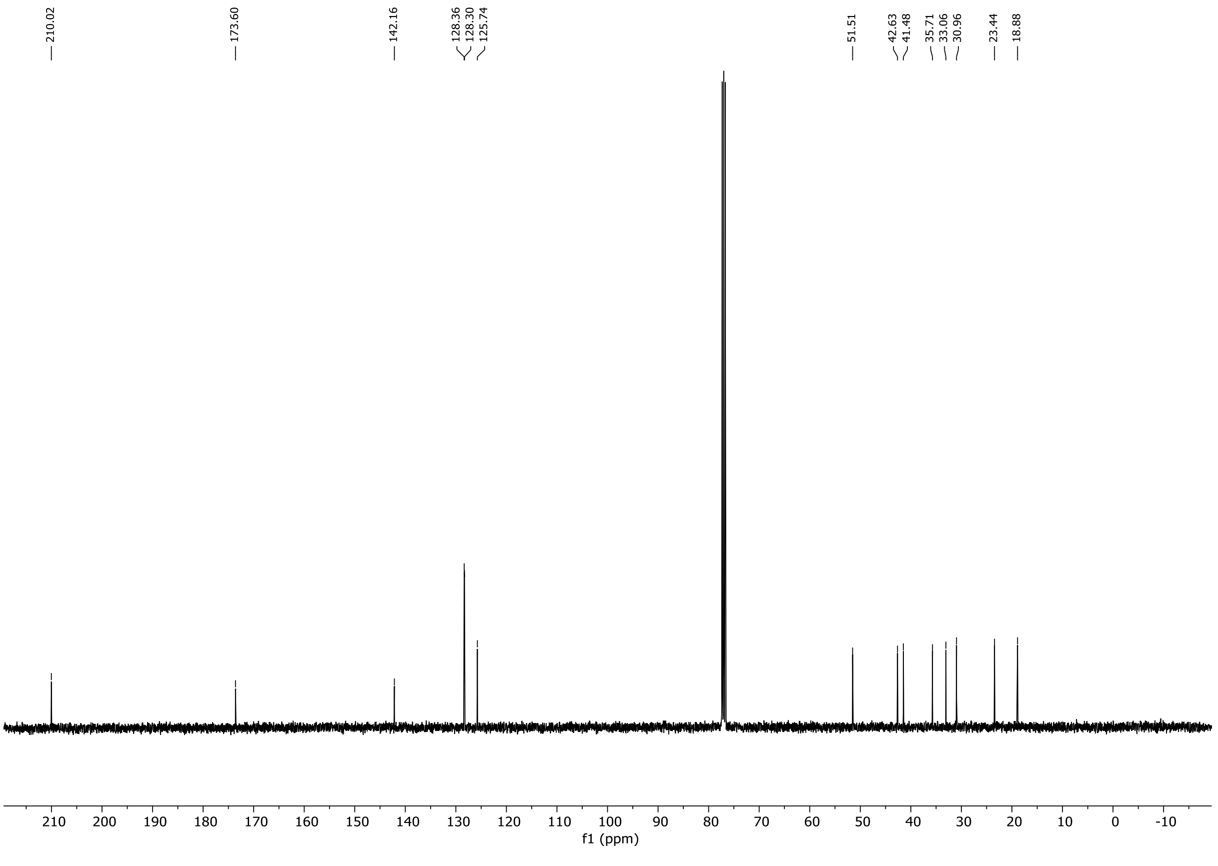

Supplement: Supplementary file 1 — ol1c03321_si_001.pdf [file ol1c03321_si_001.pdf]
